# On the thermodynamics of DNA methylation process

**DOI:** 10.1038/s41598-023-35166-9

**Published:** 2023-06-01

**Authors:** Robersy Sanchez, Sally A. Mackenzie

**Affiliations:** 1 Department of Biology, The Pennsylvania State University, 361 Frear North Bldg, University Park, PA 16802 USA; 2 Departments of Biology and Plant Science, The Pennsylvania State University, 362 Frear North Bldg, University Park, PA 16802 USA

**Keywords:** Epigenetics, DNA methylation, Applied mathematics

## Abstract

DNA methylation is an epigenetic mechanism that plays important roles in various biological processes including transcriptional and post-transcriptional regulation, genomic imprinting, aging, and stress response to environmental changes and disease. Consistent with thermodynamic principles acting within living systems and the application of maximum entropy principle, we propose a theoretical framework to understand and decode the DNA methylation process. A central tenet of this argument is that the probability density function of DNA methylation information-divergence summarizes the statistical biophysics underlying spontaneous methylation background and implicitly bears on the channel capacity of molecular machines conforming to Shannon’s capacity theorem. On this theoretical basis, contributions from the molecular machine (enzyme) logical operations to Gibb entropy (*S*) and Helmholtz free energy (*F*) are intrinsic. Application to the estimations of *S* on datasets from *Arabidopsis thaliana* suggests that, as a thermodynamic state variable, individual methylome entropy is completely determined by the current state of the system, which in biological terms translates to a correspondence between estimated entropy values and observable phenotypic state. In patients with different types of cancer, results suggest that a significant information loss occurs in the transition from differentiated (healthy) tissues to cancer cells. This type of analysis may have important implications for early-stage diagnostics. The analysis of entropy fluctuations on experimental datasets revealed existence of restrictions on the magnitude of genome-wide methylation changes originating by organismal response to environmental changes. Only dysfunctional stages observed in the Arabidopsis mutant *met1* and in cancer cells do not conform to these rules.

## Introduction

Cytosine DNA methylation is a well-characterized epigenetic modification^1,2^ that plays important roles in various biological processes, including X-chromosome inactivation, genomic imprinting, transposon suppression, transcriptional regulation, and the aging process^3–6^. Additionally, DNA methylation acts to preserve DNA stability^7,8^, which implies that the most frequent methylation changes serve to preserve thermodynamic stability of DNA molecules. These methylation changes comprise the background activity that is distinguished from targeted differentially methylated positions (DMPs) directed by methylation regulatory machinery^9^.

When evaluating samples from a single species under various experimental conditions, it is not difficult to find evidence by data analysis and simulation of differential methylation activity in control populations^9^. These DMPs are presumed to derive from fluctuations inherent to any stochastic process, a property summarized by the fluctuation theorem^10,11^. Regardless of a constant environment, statistically significant methylation changes occur in a control population with probability greater than zero, implying that stochasticity of the methylation process derives from the inherent stochasticity of biochemical systems^12–16^. Spontaneous natural methylation variation (“noise”) is expected within multicellular organisms, while methylation regulatory machinery (“signal”) directs organismal adaptation to environmental fluctuation^15,16^ and during development.

Models for the probability distribution of methylation variation (noise plus signal), expressed as information divergences of methylation levels, were derived for a constrained scenario^17^. Background methylation variation could be described in terms of a generalized gamma probability distribution or a member of a generalized gamma distribution family. However, such modeling^17^ only works as a transfer function where model parameters remain undefined, which is useful for practical applications in modeling the system's output for each possible input but not for understanding thermodynamics of the methylation process.

A formal derivation of the generalized gamma model for the cytosine DNA methylation process considers continuous action of thermodynamics on biological processes and the consequent application of Jaynes’ Maximum Entropy Principle (MEP^18^), an information-theoretical account of the Second Law^19^. Statistical physical assumptions are set on the channel capacity of molecular machines^20,21^, which is closely related to Shannon’s channel capacity. Biological molecular machines are assumed with energy scales comparable to the thermal energy $k_{B} T$ at ambient temperature with sensitivity to thermal fluctuation^22,23^.

The proposed modeling provides a physical interpretation for parameters not previously undertaken. We show that spontaneous variation in cytosine DNA methylation arises by continuous action in compliance with thermodynamic principles. Contributions from molecular machine logical operations to Gibb entropy (*S*) and Helmholtz free energy (*F*) are included in corresponding classical equations, which we subsequently apply to the estimation of *S* and *F* in experimental datasets of a plant epigenetics model system in *Arabidopsis thaliana* and in patients with different types of cancer. Data emerging from this type of analysis provide important insight into an organism’s biological state and, perhaps, to new approaches for early diagnostics.

## Results

**Figure 1:**
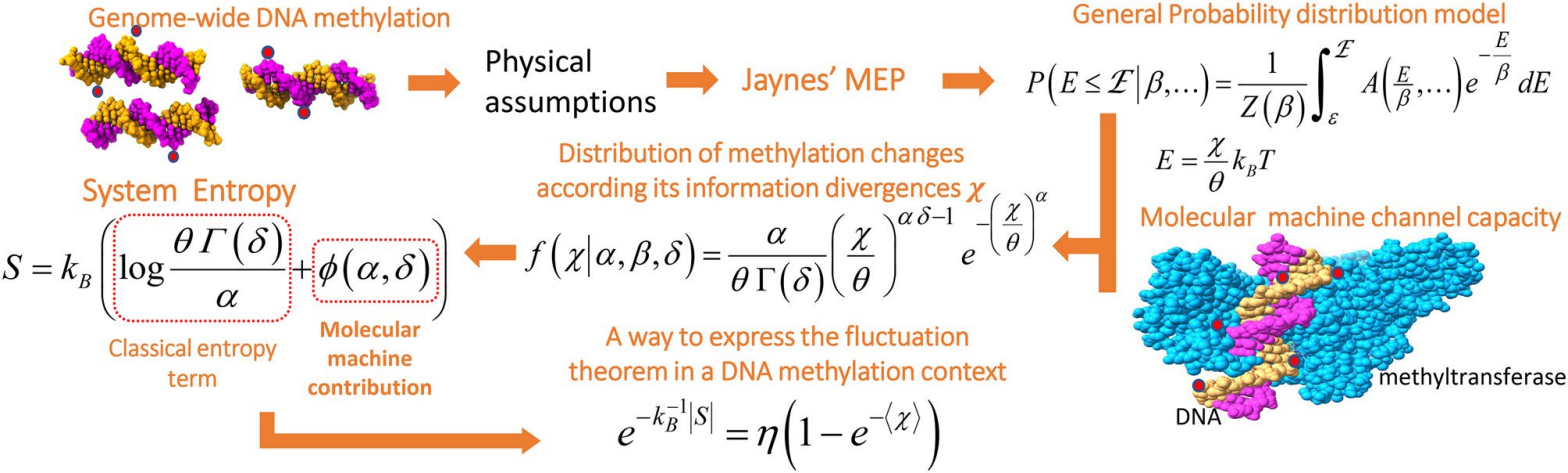
Flow chart of the analytical steps followed in the study of methylation process thermodynamics. **a,** Application of Jaynes’ Maximum Entropy Principle (MEP^18^) leads to Boltzmann distribution as most probable for the methylation system^18,24^. Criteria derived from molecular machine channel capacity and further maximum likelihood estimations lead to the theoretical derivation of a generalized gamma distribution model as best to describe genome-wide methylation changes observable in an individual dataset. This model is expressed in terms of information divergence of methylation changes $\chi:$ $E = \chi k_{B} T\theta^{ - 1}$. The state of the methylation system is described by generalized gamma probability density function, from which analytical expression for methylation system entropy is derived. Analysis of experimental datasets from Arabidopsis and human cancer allow expression of the fluctuation theorem in a DNA methylation context.

The flow chart presented in Fig. 1 summarizes the relevant analytical steps followed in our study. In biochemical terms, methylation changes to DNA molecules are biochemical reactions accomplished by two types of enzymes: methyltransferases and demethylases. These enzymes, as molecular machines, accomplish methylation changes through several logical operations that require, according to Landauer’s principle ^24,25^, a minimum energy dissipation $\varepsilon = k_{B} T\ln 2$ per bit of information per machine operation^26^. At human body temperature, 310.15 K, $\varepsilon =1.784\mathrm{\ J} \times {\mathrm{\ mol}}^{-1}$. Thus, any methylation change involves an associated energy dissipation $E \ge k_{B} T\ln 2$ per bit of information per machine operation, where $k_{B}$ and *T* stand for Boltzmann constant and the absolute temperature, respectively.

The application of MEP and constraints from molecular machine channel capacity describe the methylation process as a probability distribution $f\left( E \right)$ of the energy dissipated *E* (Fig. 1). With this approach, estimations of the individual methylation system entropy and Helmholtz free energy are feasible, and biological implications of the theory can be assessed in appropriate whole-genome methylome datasets.

### Statistical-physical modeling of the methylation background process

The most probable distribution of methylation states for a DNA molecule, driven by spontaneous/random fluctuations, can be obtained by maximizing the thermodynamic entropy under general system constraints: *i*) $\sum\nolimits_{i} {\pi_{i} } = 1$ and *ii*) $\sum\nolimits_{i} {\pi_{i} E_{i} } = \left\langle E \right\rangle$, where $\pi_{i}$ is the (discrete) probability to observe dissipation of the energy value $E_{i}$, and $\left\langle E \right\rangle$ is the mathematical expectation of $E$. Under these assumptions, Jaynes’ MEP leads to Boltzmann distribution as the most probable distribution of the system^18,27^. Assuming that the energies $E_{i}$ dissipated to reach the states *i* of the system are essentially a continuum, with some density $A\left( {\tfrac{E}{\beta }, \ldots } \right)$ of methylation changes and energies dissipated *E*, the probability to observe genome-wide energy dissipation between 0 and *E* can be estimated^28^ as:

1 $$P\left( {E \le \left. {\mathcal{E}} \right|\,\beta , \ldots } \right) = \frac{1}{Z\left( \beta \right)}\int_{\varepsilon }^{\mathcal{E}} {A\left( {E,\beta , \ldots } \right)e^{{ - \,\frac{E}{\beta }}} } \,dE$$

where $Z\left( {\beta ,...} \right) = \int_{\varepsilon }^{\infty } {A\left( {E,\beta , \ldots } \right)\,e^{{ - \,\frac{E}{\beta }}} } \,dE$ stands for the partition function of the system and $\beta = k_{B} T$ is a scaling constant. That is, the number of methylation changes per unit energy at *E* ($A\left( {E,\beta , \ldots } \right)dE$) is the number of methylation changes with energies dissipated per bit of information in the infinitesimal range *E* to *E* + *dE.* In Eq. (1), expression under the integral together with the partition function is, by definition ^28^, a probability density function denoted as:

2 $$f\left( {\left. E \right|\beta , \ldots } \right) = \frac{1}{{Z\left( {\beta , \ldots } \right)}}A\left( {E,\beta , \ldots } \right)e^{{ - \frac{E}{\beta }}}$$

Notice that for $A\left( {E,\beta , \ldots } \right) = 1$, the last equation reduces to the classical expression for Boltzmann distribution. Equation (2) is a general probabilistic model of the methylation background process conforming to an exponential decay law. According to Eq. (2), it is expected that for any case of $f\left( {\left. E \right|\beta , \ldots } \right)$, the probability to observe a methylation change will decline with the increment of energy dissipated per bit of information processed by molecular machines (methyltransferase and demethylase activity). In the following sections, we set out information-thermodynamic constraints on the molecular methylation machinery that permit a maximum likelihood estimation of function $f\left( {\left. E \right|\beta , \ldots } \right)$.

### The channel capacity of methylation machinery

A fundamental constraint to deriving a probability density function of DNA methylation changes involves physics of information in molecular machine operations. Machine capacity is closely related to Shannon’s channel capacity^29^ as the maximum amount of information that a molecular machine can gain per operation^20^. Following Schneider^20^, the machine capacity is bounded by: $C = d_{space} \log_{2} \frac{{P_{y} + N_{y} }}{{N_{y} }}$, where $P_{y}$ is the energy dissipated by a molecular machine,$\ N_{y}$ energy of the thermal noise, and $d_{space}$ the number of independently moving parts of a molecular machine involved in the operation^21^. Following Shannon^29^, the received signals have an energy average $E_{y} = P_{y} + N_{y}$. We shall denote by $E^{0} = N_{y}$ the energy dissipated with probability = 1 and $d_{space} = \nu - 1$ to arrive at $C_{v} = \left( {\nu - 1} \right)\log_{2} \frac{{E_{y} }}{{E^{0} }}$ ($\nu = \alpha \,\delta$, Supplementary Information (SI) section A ), which implies:

3 $$\left( {\nu - 1} \right)\log_{2} \frac{{E_{i} }}{{E^{0} }} \le C_{v}$$

### Probability density function of the methylation background changes

Equations 1 and 2 quantitatively summarize the statistical physics underlying methylation changes that are *not* induced by the methylation regulatory machinery. Application of thermodynamic principles to chromatin dynamics tends to maximize Boltzmann entropy, leading to the most probable methylation density states. We sought to maximize the probability ${\varvec{P}}\left( {N_{1} , \ldots ,N_{k} ,N,p_{1} , \ldots ,p_{k} } \right)$ that *N* distinguishable methylation events result in $N_{1} , \ldots ,N_{k}$ ($\sum\nolimits_{i}^{k} {N_{i} = N}$) outcomes in the intervals $\left[ {E_{0} ,E_{1} } \right), \cdots ,\left[ {E_{k - 1} ,E_{k} } \right)$ with probabilities $p_{1} , \ldots ,p_{k}$. Two basic assumptions were imposed on $p_{i}$, $N_{i}$ and $E_{i}$:

1. probabilities $p_{i}$ are proportional to a specific power of the energies $E_{i}$:
  4 $$p_{i} = \left( {\frac{{E_{i} }}{{E^{0} }}} \right)^{\nu - 1}$$
2. for each choice of $\alpha$ the following sum is a positive constant:
  5 $$\sum\nolimits_{i = 1}^{k} {N_{i} E_{i}^{\alpha } } = E_{const}$$

where $E_{i} > 0$; $N_{i}$’s are assumed to be large numbers.

The first assumption derives from the interpretation of channel capacity of molecular machines given by Eq. (3) ^20 ^as $\log_{2} p_{i} \le C_{v}$. The second assumption implies that parameter α carries information about the molecular machine, since $\nu = \alpha \,\delta$ (SI A). A maximum likelihood estimation of function $f\left( {\left. E \right|\beta , \ldots } \right)$, on a thermodynamic basis, adapts the Lienhard and Meyer approach^30^ to the specific scenario of DNA methylation (provided in the SI A). The above assumptions (not given in^30^) lead to the generalized gamma probability density function:

6 $$f\left( {\left. E \right|\alpha ,\beta ,\delta } \right) = \frac{\alpha }{\beta \;\Gamma \left( \delta \right)}\left( {\frac{E}{\beta }\,} \right)^{\alpha \,\delta - 1} e^{{ - \left( {\frac{E}{\beta }} \right)^{\alpha } }}$$

where $\alpha > 0,\beta > 0,\,\delta > 0$, and $E > 0$. Consistent with Eq. (2), the analytical expression for the partition function derives from Eq. (6):

7 $$Z\left( \beta \right) = \int_{0}^{\infty } {\left( {\frac{E}{\beta }} \right)^{\alpha \,\delta - 1} \,e^{{ - \left( {\frac{E}{\beta }} \right)^{\alpha } }} } dE = \frac{\beta \,\Gamma \left( \delta \right)}{\alpha },$$

Hence, the density $A\left( {E,\beta , \ldots } \right)$ can be expressed as:

8 $$A\left( {\left. E \right|\alpha ,\beta ,\delta } \right) = \left( {\frac{E}{\beta }} \right)^{\alpha \,\delta - 1} \,e^{{ - \left( {\frac{E}{\beta }} \right)^{\alpha - 1} }}$$

An information-theoretic divergence $\chi \left( {p,q} \right)$ of methylation levels *p* and *q* will follow a distribution derived from Eq. (1) (generalized gamma, gamma, or Weibull distribution model) provided that it is proportional to the energy *E*. In this case, the energy dissipated *E* is per bit of information associated to the corresponding methylation changes. In general, for an information-theoretic divergence measure of methylation levels $\chi \left( {p,q} \right)$, we can follow the same analytical steps used to derive Eq. (6) (see SI A), which leads to a probability density function for the information divergence $\chi \left( {p,q} \right)$:

9 $$f\left( {\left. \chi \right|\alpha ,\theta ,\psi } \right) = \frac{\alpha }{\theta \;\Gamma \left( \delta \right)}\left( {\frac{\chi }{\theta }\,} \right)^{\alpha \,\delta - 1} e^{{ - \left( {\frac{\chi }{\theta }} \right)^{\alpha } }}$$

Assuming that $\frac{E}{{k_{B} T}} = \frac{\chi }{\theta }$ ($\chi$ in bit units), the energy dissipated can be estimated as:

10 $$E = \frac{\chi }{\theta }k_{B} T$$

According to Landauer’s principle, a molecular machine working under ideal conditions dissipates the minimum energy $E = \chi k_{B} T\ln 2$, with $\theta = {1 \mathord{\left/ {\vphantom {1 {\ln 2}}} \right. \kern-0pt} {\ln 2}}$ in ideal conditions. A more general distribution that includes the location parameter $\mu$ is given as:

11 $$f\left( {\left. \chi \right|\alpha ,\theta ,\mu ,\delta } \right) = \frac{\alpha }{\theta \;\Gamma \left( \delta \right)}\left( {\frac{\chi - \mu }{\theta }\,} \right)^{\alpha \,\delta - 1} e^{{ - \left( {\frac{\chi - \mu }{\theta }} \right)^{\alpha } }}$$

which has mean:

11a $$\nu = \frac{{\mu \Gamma \left( \delta \right) + \theta \,\Gamma \left( {\delta + \frac{1}{\alpha }} \right)}}{\Gamma \left( \delta \right)}$$

and variance:

11b $$\sigma = \frac{{\mu^{2} \Gamma \left( \delta \right) + 2\mu \theta \,\Gamma \left( {\frac{1}{\alpha } + \delta } \right) + \theta^{2} \Gamma \left( {\frac{2}{\alpha } + \delta } \right)}}{\Gamma \left( \delta \right)}$$

$\chi \left( {p,q} \right)$ can be expressed in terms of the Hellinger divergence given by Sanchez et al. ^9^ or in terms of *J*-divergence^31^. The most frequent members of a general gamma distribution family found by goodness-of-fit tests for processed bisulfite sequence datasets from different species are Weibull ($\delta = 1$) and Gamma ($\alpha = 1$) distributions^9,32^, obtained as particular cases from the generalized gamma probability density function.

### A connection with Shannon’s communication theory

As suggested in past reports^17,33^, genome-wide patterning of cytosine DNA methylation can occur at specific landmarks, statistically alluding to the existence of a methylation language/code^33,34^, where methylation messages are created within the framework of a communication system. In terms of Shannon’s communication theory, a communication system can be described by the conditional probability (density) $P_{x} \left( y \right)$, so that if message *x* is produced by the source, the recovered message at the receiving point will be *y* ^29^. Shannon defined the rate $R_{1}$ of generating information for a given quality $v_{1} = \iint {\rho \left( {x,y} \right)}{\kern 1pt} P\left( {x,y} \right)dx{\kern 1pt} dy$ of reproduction to be $R = \mathop {Min}\limits_{{P_{x} \left( y \right)}} \iint {P\left( {x,y} \right)\log \frac{{P\left( {x,y} \right)}}{P\left( x \right)P\left( y \right)}}dx{\kern 1pt} dy$ at fixed $v_{1}$ and variable $P_{x} \left( y \right)$.

In Shannon’s analysis, the conditional probability $P_{y} \left( x \right)$ that minimizes the rate $R$ is given by the expression $P_{y} \left( x \right) = B\left( x \right)e^{{ - \lambda \rho \left( {x,y} \right)}}$, where $B\left( x \right)$ is chosen to satisfy $\int {B\left( x \right)e^{{ - \lambda {\kern 1pt} \rho \left( {x,y} \right)}} dx} = 1$ ^29^, and $\rho \left( {x,y} \right)$ is a distance function. In this analysis, function $\rho \left( {x,y} \right)$ behaves as a “distance” between *x* and *y* to measure the unlikelihood, based on a fidelity criterion, to receive *y* with transmission of *x*. In function $B\left( x \right)$, the transmitted message $x$ can be expressed at each cytosine site in terms of observed methylation levels in a treatment or a patient group. Methylation levels are estimated as: ${{nC_{i}^{m} } \mathord{\left/ {\vphantom {{nC_{i}^{m} } {\left( {nC^{m} + nC_{i} } \right)}}} \right. \kern-0pt} {\left( {nC^{m} + nC_{i} } \right)}}$, where $nC_{i}^{m}$ and $nC_{i}$ are the number of times the cytosine is methylated and unmethylated at site $i$, respectively. The received message *y* can be specified as reference methylation levels, which could be the centroid of a group control or estimation from an independent subset of control samples from a control population. The function $\rho \left( {x,y} \right)$ can be expressed in terms of a symmetric information divergence $\chi \left( {x,y} \right)$ between the methylation levels *x* and *y*. For a fixed reference *y*, the equality $\chi \left( {x,y} \right) = \chi \left( x \right)$ makes it possible to choose $B\left( x \right)$ as:

12 $$B\left( x \right) = \chi ^{\prime}\left( x \right)\frac{\alpha }{\theta \;\Gamma \left( \delta \right)}\left( {\frac{\chi \left( x \right)}{\theta }\,} \right)^{\alpha \,\delta - 1} e^{{ - \left( {\frac{\chi \left( x \right)}{\theta }} \right)^{\alpha - 1} }}$$

where $d\chi = \chi ^{\prime}\left( x \right)dx$ and $\lambda = {1 \mathord{\left/ {\vphantom {1 \theta }} \right. \kern-0pt} \theta }$. The conditional probability ${\mathbb{P}}_{y} \left( x \right)$, if the recovered message at the receiving point is $y$ and the original message produced by the source is $x$, can be reinterpreted (after change of variables) as:

13 $${\mathbb{P}}_{y} \left( {\chi \left| {\alpha ,\delta ,\theta } \right.} \right) = \int_{0}^{\chi } {\frac{\alpha }{\theta \;\Gamma \left( \delta \right)}\left( {\frac{\chi }{\theta }\,} \right)^{\alpha \,\delta - 1} e^{{ - \left( {\frac{\chi }{\theta }} \right)^{\alpha } }} d\chi }$$

This equation indicates the probability that, if the recovered message at the receiving point is $y$, then the information divergence between $y$ and the original message $x$ produced by the source is $\chi$. These applications of Shannon’s reasoning lead to the following:

#### Theorem 1

If an organismal methylation system conforms to a communication system, then optimal methylation messaging is described by Eqs. (13), (9).

### The Gibb entropy of the system

The Gibb entropy of a system resulting from methylation changes is defined by the integral:

14 $$\Delta S = - k_{B} \int_{0}^{\infty } {f\left( {\left. E \right|\alpha ,\beta ,\delta } \right)\ln f\left( {\left. E \right|\alpha ,\beta ,\delta } \right)dE}$$

(or simply *S*, since S(0) = 0) which yields the known analytical expression (SI B):

15 $$S = k_{B} \left( {\ln \frac{\beta \,\Gamma \left( \delta \right)}{\alpha } + \psi \left( \delta \right)\left( {\frac{1}{\alpha } - \delta } \right) + \delta } \right)$$

where $\psi \left( \delta \right) = \frac{d\ln \Gamma \left( \delta \right)}{{d\delta }}$ stands for the digamma function. After considering Eq. (6), we can write:

15a $$S = \underbrace {{k_{B} \ln \frac{\beta \,\Gamma \left( \delta \right)}{\alpha }}}_{\substack{ {\text{Classical entropy }} \\ {\text{term}} } } + \underbrace {{k_{B} \psi \left( \delta \right)\left( {\frac{1}{\alpha } - \delta } \right) + k_{B} \delta }}_{\substack{ {\text{Molecular machine }} \\ {\text{moving parts contribution}} } }$$

Thus, entropy of an individual methylation system is split into a classical term and contribution from molecular machine activity:

16 $$S = S_{classic} + S_{machine}$$

A rough estimation of Gibb entropy for different organismal tissues/cells can be based on the information divergence $\chi_{i}$ after expressing energy $E_{i}$ in terms of $\chi_{i}$ according to Eq. (9):

17 $$S = k_{B} \left( {\ln \frac{\theta \,\Gamma \left( \delta \right)}{\alpha } + \phi \left( {\alpha ,\delta } \right)} \right)$$

where the term $\phi \left( {\alpha ,\delta } \right) = \psi \left( \delta \right)\left( {\frac{1}{\alpha } - \delta } \right) + \delta$ is a function of a model parameter associated to the number of independent activities of the molecular machine ($\nu = \alpha {\kern 1pt} \delta$).

Since $\log_{2} x = {{\ln x} \mathord{\left/ {\vphantom {{\ln x} {\ln 2}}} \right. \kern-0pt} {\ln 2}}$, Eq. (17) can be written as:

17a $$S = k_{B} \ln 2\left( {\log_{2} \frac{\theta \,\Gamma \left( \delta \right)}{\alpha } + \frac{{\phi \left( {\alpha ,\delta } \right)}}{\ln 2}} \right)$$

The terms in brackets from Eq. (17) and (17a) (at constant temperature) correspond to Shannon entropy *H*, which depends only on the distribution parameters in this case, numerical values that can be estimated from experimental data for each individual. Thus, the Shannon entropy *H* can be written as:

18 $$H = \log_{2} \frac{\theta \,\Gamma \left( \delta \right)}{\alpha } + \frac{{\phi \left( {\alpha ,\delta } \right)}}{\ln 2}$$

and

18a $$S = k_{B} \ln 2H$$

Following Schneider^26^, a decrease in methylome entropy:

19 $$\Delta S = S_{after} - S_{before}$$

requires a corresponding decrease in the uncertainty of genome-wide methylation changes:

20 $$\Delta H = H_{after} - H_{before}$$

Following a decrease in this uncertainty, the methylome gains information $I_{m}$:

21 $$I_{m} \equiv - \Delta H$$

That is,

21a $$\Delta {\text{H}}=-\frac{\Delta {\text{S}}}{{\text{k}}_{\text{B }}{\text{ln2}}}$$

Or expressed in Joule per Kelvin:

21b $$I_{m} \equiv - k_{B} \ln 2\Delta H$$

Information-theoretical entropy and thermodynamic entropy yield identical outcomes, up to the product of Boltzmann’s constant by ln 2, even though they are independent functions^19^.

### Thermodynamic potential of methylation changes

Assuming that a balance exists between methylation and demethylation processes along each DNA molecule, the overall mass (number of molecules *N*) and volume (*V*) of the DNA molecule remain constant. This assumption holds in most experimental datasets since, for large genomic regions, the sum of the difference in methylation level is close to zero. Under this condition, and assuming a constant temperature (*T*), methylation changes and the micro-environment around them can be treated as a closed system to mass transport but not energy transfer. In statistical physics, this system is referred to as a NVT system, with the thermodynamic variables *N*, *V*, and *T* held fixed. Helmholtz free energy $\left( F \right)$ represents the driving force for NVT systems, the thermodynamic potential that measures “useful” work obtainable from a closed system at a constant temperature and volume.

Helmholtz free energy can be estimated from its definition: $F = U - TS$. Assuming that the molecular machine operations do not change the internal energy *U* of the system, we have: $\Delta F = - T\Delta S$, i.e.:

22 $$\Delta F = - \beta \left( {\log \frac{\beta \,\Gamma \left( \delta \right)}{\alpha } + \psi \left( \delta \right)\left( {\frac{1}{\alpha } - \delta } \right) + \delta } \right)$$

The same result derives from the Gibbs free energy definition: $G = H - TS$. Considering that the molecular machine operations do not change the system pressure ($\Delta H = 0$): $\Delta G = - T\Delta S$. Equation (22) roughly estimates how much Helmholtz free energy would be involved in methylation. Rough estimations based on the information divergence $\chi$ can use the approach:

23 $$\Delta F = - \beta \left( {\ln \frac{\theta \,\Gamma \left( \delta \right)}{\alpha } + \phi \left( {\alpha ,\delta } \right)} \right)$$

where $\beta = k_{B} T$. Considering Eq. (16,) Helmholtz free energy can be split into the classical term and contribution of molecular machine activities:

24 $$\Delta F = - \beta S_{classic} - \beta S_{machine} = \Delta F_{classic} + \Delta F_{machine}$$

According to Eq. (7): $\Delta F_{classic} = k_{B} T\ln Z = k_{B} T\ln \frac{\theta \,\Gamma \left( \delta \right)}{\alpha }$. The particular cases of $S_{G}$ and $F\left( \beta \right)$ for Weibull and Gamma distributions are obtained with parameter values $\delta = 1$ and $\alpha = 1$, respectively. Substitution of Eq. (17a) in Eq. (23) yields:

25 $$\Delta F = - \beta \ln 2H$$

At constant temperature,$\ \Delta F$ decreases with the increment of Shannon entropy of the system. The variation of Helmholtz free energy $\Delta \Delta F = \Delta F_{after} - \Delta F_{before}$ between two system states (*before* and *after*) can be expressed as:

26 $$\Delta \Delta F = \Delta F_{after} - \Delta F_{before}$$

After considering Eqs. (20), (21), and (25), an energetically favorable process is:

26a $$\Delta \Delta F = T{\kern 1pt} I_{m}$$

where a loss of information ($I_{m} < 0$) will be associated with a loss of free energy $\Delta \Delta F < 0$.

### Biological implications of these observations

The theoretical framework presented can be summarized into two biologically intuitive hypotheses:

1. The entropy of methylation variation, measured with respect to some reference, coincides with observable phenotypic change. Thus, entropy provides a highly sensitive measure of organismal epigenetic state.
2. Disruption of methylation machinery will generate large fluctuations in the methylation signal outside of the expected range of fluctuations for normal/healthy tissues.

The first hypothesis rests on the premise that entropy is a thermodynamic state variable of the system, which means that its value is completely determined by current state of the system and not by how the system reached that state. The second hypothesis presumes that methylation machinery participates in organismal adaptation to environmental changes, and this process requires a non-equilibrium feedback control. To adapt to environmental change, organisms must rely on molecular mechanisms to sense changes and trigger regulatory adaptative responses^35^.

To test our hypotheses, we analyzed *Arabidopsis thaliana* and human methylome datasets. Functions for Gibbs entropy and Helmholtz free energy estimations, as given by Eqs. (17) and (22), respectively, are currently included in MethylIT R package (see Supporting Information). Entropy was estimated in *Arabidopsis thaliana* Col-0 ecotypes (wild type controls, WT), the methyltransferase mutant *met1*^36^, and first- and third-generation heritable epigenetic memory states (*nm1*, *mm1*, and *mm3)* that derive as epigenetically modified progeny from a parental line following suppression of *MSH1* expression^37^.

In plants, CG methylation is maintained by *METHYLTRANSFERSE1* (*MET1*), and mutations that disrupt its activity induce genome-wide hypomethylation in CG context. Consequently, we expect to observe a significant loss of information in datasets from *met1* plants relative to wild type. In the case of *msh1* memory state, heritable epigenetic stress memory is observed following segregation of an *MSH1*-RNAi transgene, yielding ca. 20% of transgene-null progeny with a heritable memory phenotype of delayed maturation and sustained stress response (*mm1, mm3*), and the remainder appearing phenotypically unchanged and designated “non-memory” (*nm1*). The *msh1* memory system was described previously^37^, and both memory (*mm1)* and non-memory (*nm1*) full-sib types display evidence of genome-wide cytosine methylation repatterning relative to wild type. Here, we include analysis of first-generation (*mm1*) and third-generation (*mm3*) samples from the same *msh1* memory lineage and predict these variants to display lesser incremental effect on entropy variation than *met1*. Results shown in Table 1 confirm these predicted outcomes.

**Table 1:** Gibb entropy^1^ estimated in several Arabidopsis mutants and corresponding Col-0 controls (WT).

Treatment	Gibb entropy by individual chromosome					^2^$I_{m}$	^3^$\Delta \Delta F$
	1	2	3	4	5		
WT3-1	−12.095	−13.092	−12.854	−12.875	−12.398		
WT3-2	−12.239	−13.202	−12.827	−12.955	−12.447		
WT3-3	−12.582	−13.611	−13.312	−13.403	−12.872		
WT3-4	−12.190	−13.289	−12.884	−13.008	−12.534		
WT3-5	−13.010	−14.074	−13.806	−13.831	−13.333		
nm1_1	−10.517	−11.671	−11.43	−11.447	−10.970	−0.612 **†^4^	−189.8**†
nm1_2	−10.344	−11.461	−11.193	−11.205	−10.758		
nm1_3	−13.424	−14.234	−14.126	−14.175	−13.761		
nm1_4	−10.332	−11.428	−11.16	−11.192	−10.74		
nm1_5	−14.458	−14.972	−15.002	−14.804	−14.614		
mm1_1	−12.452	−13.385	−13.153	−13.134	−12.807	−1.140***	−353.63***
mm1_2	−13.170	−14.111	−13.934	−13.978	−13.579		
mm1_3	−10.485	−11.578	−11.391	−11.369	−10.947		
mm1_4	−10.087	−11.177	−10.972	−10.982	−10.485		
mm1_5	−9.969	−11.104	−10.818	−10.852	−10.298		
mm3_1	−9.504	−10.593	−10.366	−10.370	−9.850	−2.627***	−814.79***
mm3_2	−9.617	−10.691	−10.537	−10.528	−10.014		
mm3_3	−9.392	−10.475	−10.269	−10.264	−9.839		
mm3_4	−10.336	−11.407	−11.292	−11.310	−10.825		
mm3_5	−9.688	−10.736	−10.531	−10.526	−10.083		
WT_met1__1	−3.751	−4.061	−3.958	−3.738	−3.700		
WT_met1__2	−5.876	−6.242	−6.164	−5.959	−5.811		
WT_met1__3	−5.869	−6.216	−6.070	−5.896	−5.727		
WT_met1__4	−5.994	−6.347	−6.178	−5.995	−5.889		
met1_1	2.183	2.129	2.065	1.980	2.085	−7.185***	−2228.45***
met1_2	1.199	1.126	1.072	1.004	1.108		
met1_3	2.032	1.993	1.923	1.848	1.946		

^1^Entropy values were estimated using Eq. (17) and *J*-divergence ^31^. The values are given in $\mathrm{J}\times {\mathrm{K}}^{-1}\times {\mathrm{mol}}^{-1}$, after replacing Boltzmann constant by the Gas constant. ^2^ Loss of Information $I_{m}$ is given by Eq. (20a). ^3^Helmholtz free energy $\Delta \Delta F$ values were estimated using Eq. (26a) and *J*-divergence ^31^. The values are given in $\mathrm{J}\times {\mathrm{mol}}^{-1}$. ^4^Symbols ‘**’ and ‘***’ indicate highly statistically significant differences at p-value < 0.01 and p-value < 10^–16^ between mutant or memory state, respectively. Symbol † indicates Wilcoxon paired test, otherwise testing was conducted applying linear mixed model.

The effect of an *msh1* suppression line on genome-wide methylation changes in epigenetic memory and non-memory progeny, generations 1 and 3, was reflected in a discrete increment of entropy and, consequently, loss of information: $\Delta S = S_{control} - S_{mutant} < 0$^26^. This observation is further evidence of epigenetic effects that give rise to the memory state^37^. Loss of information in the *met1* mutant was much greater than in *msh1* memory, consistent with the profound effects of genome-wide CG demethylation; CG is the predominant genic methylation context in animals and plants.

Our results suggest that entropy can serve as a highly sensitive measure of the state of an organism. For example, we also observed significant differences in the entropy values for Col-0 wildtype controls WT3 and WT_*met1*_. Although these wildtype controls derive from the same Arabidopsis Col-0 accession, they differ in ontogeny. WT_*met1*_ plants were grown under continuous light for 2 weeks in half-strength Gamborg's B5 media, while WT3 plants were grown to maturity on standard peat mix in pots maintained at 12-h daylength and sampled at bolting stage. We consider these differences in plant stage and growth conditions to account for the marked entropy differences observed.

In human cancer studies, Gibb entropies for different cancer cells and the corresponding healthy tissue/cell controls are presented in Table 2. Outcomes suggest that Gibb entropy increases for all cancer cells relative to their corresponding normal tissue. Since information divergences were computed with respect to the same reference individual, the observed entropy values suggest that breast metastasis cells underwent the most aggressive loss of information (assuming that experimental errors were not sufficient to affect the estimated values). The relationship between Gibb entropy and Helmholtz free energy predicts results shown in Table 3. After the methylation reprogramming that transforms differentiated healthy cells to a cancer state, the information potential of cancer cells appears to decrease dramatically relative to healthy cells. These data reflect an important, previously undocumented, means of assessing the state of a biological system. The overall results support our hypothesis that entropy estimation is a highly sensitive measure of organismal epigenetic state.

**Table 2:** Gibb entropy^1^ estimated in human cancer cells and corresponding normal tissue.

Tissue2	Chromosome											
	1	2	3	4	5	6	7	8	9	10	11	12
Brain	−16.34	−16.49	−16.65	−16.79	−16.48	−16.53	−16.51	−16.38	−16.13	−16.26	−16.02	−16.41
Glioma	−1.54	−1.52	−2.29	−2.66	−1.26	−2.15	3.60	−0.82	−2.01	0.47	0.05	−1.38
Breast	−14.13	−14.64	−14.77	−15.05	−14.62	−14.62	−14.56	−14.37	−14.08	−14.20	−13.88	−14.33
Breast Cancer	−1.04	−0.09	0.50	1.94	2.89	−0.77	2.39	1.63	−1.27	2.39	2.19	1.25
Breast Metastasis	2.61	4.75	4.65	6.79	6.07	3.56	4.73	6.68	3.24	5.65	5.22	3.42
Colon	−14.50	−14.68	−14.77	−14.48	−14.46	−14.87	−14.78	−14.52	−14.38	−14.71	−14.18	−14.74
Colon Cancer	−9.93	−10.46	−10.87	−9.57	−9.47	−10.65	−10.09	−9.27	−10.33	−10.39	−9.70	−10.52
Colon Metastasis	−6.65	−6.16	−6.40	−5.53	−4.80	−6.22	−5.18	−4.23	−6.44	−5.93	−5.21	−6.52
Lung	−16.51	−16.78	−16.86	−17.19	−16.90	−16.82	−16.73	−16.80	−16.55	−16.62	−16.55	−16.65
Lung Cancer	−8.38	−9.62	−9.90	−7.90	−6.79	−6.84	−8.03	−6.22	−8.52	−6.39	−6.31	−6.99
Adenocarcinoma	0.19	0.56	1.43	4.47	3.70	0.12	1.30	3.61	0.30	1.37	0.86	0.58
Squamous Cancer	2.92	5.30	4.77	6.07	6.25	5.67	5.10	7.12	3.86	5.66	5.90	3.71
hesc_1	1.99	1.96	1.95	1.80	1.86	1.95	1.98	1.90	1.99	1.96	1.91	1.97
hesc_2	1.66	1.62	1.61	1.41	1.49	1.61	1.65	1.53	1.64	1.60	1.54	1.64
hesc_3	1.73	1.70	1.69	1.53	1.60	1.69	1.72	1.64	1.73	1.70	1.65	1.72

	13	14	15	16	17	18	19	20	21	22	X	
Brain	−16.56	−16.12	−16.39	−15.87	−15.96	−16.42	−16.10	−15.72	−15.99	−15.64	−17.69	
Glioma	−1.84	−1.45	−1.92	−0.65	−2.33	−1.69	3.93	0.10	−0.23	−0.97	0.94	
Breast	−15.05	−14.24	−14.49	−13.42	−13.61	−14.58	−13.10	−13.47	−13.47	−12.91	−15.32	
Breast Cancer	3.12	0.29	0.05	2.12	−4.08	2.20	−3.64	0.28	1.06	−1.47	5.68	
Breast Metastasis	6.29	4.67	3.00	3.55	2.14	6.90	2.61	4.86	5.77	2.88	7.50	
Colon	−14.56	−14.58	−15.03	−14.50	−14.42	−14.60	−14.11	−13.94	−14.32	−13.97	−15.25	
Colon Cancer	−8.89	−10.10	−10.52	−9.61	−10.71	−8.68	−9.87	−8.38	−8.80	−10.04	−8.93	
Colon Metastasis	−4.50	−6.11	−6.53	−5.74	−7.78	−5.41	−6.15	−4.43	−4.44	−7.69	−2.41	
Lung	−17.00	−16.66	−16.74	−16.10	−15.97	−16.90	−16.10	−16.07	−16.24	−15.62	−16.60	
Lung Cancer	−9.00	−8.80	−9.00	−7.55	−7.81	−9.59	−3.28	−5.39	−6.21	−9.85	−3.90	
Adenocarcinoma	4.92	0.45	5.27	0.95	−1.69	4.41	−0.77	0.62	1.08	−1.24	4.54	
Squamous Cancer	4.68	4.37	4.18	3.26	−0.42	6.82	0.76	5.80	3.65	1.06	7.84	
hesc_1	1.88	1.95	1.98	2.10	2.08	1.90	2.09	1.97	1.99	2.15	0.52	
hesc_2	1.51	1.61	1.65	1.76	1.79	1.53	1.80	1.59	1.62	1.84	0.05	
hesc_3	1.61	1.69	1.72	1.84	1.83	1.63	1.85	1.70	1.73	1.90	0.21	

^1^Energy values were estimated using Eq. (16) and *J*-divergence ^31^. The values are given in $\mathrm{J}\times {\mathrm{K}}^{-1}\times {\mathrm{mol}}^{-1}$.

^2^HESC: human embryonic stem cell values are provided as reference for an undifferentiated tissue.

**Table 3:** Helmholtz free energy^1^ estimates in cancer cells and corresponding normal tissue.

Tissue^2^	Chromosome											
	1	2	3	4	5	6	7	8	9	10	11	12
Brain	5.066	5.115	5.165	5.206	5.110	5.128	5.120	5.081	5.003	5.044	4.967	5.089
Glioma	0.479	0.472	0.710	0.824	0.390	0.666	−1.115	0.255	0.622	−0.147	−0.016	0.427
Breast	4.382	4.541	4.581	4.668	4.535	4.535	4.515	4.458	4.366	4.403	4.304	4.446
Breast Cancer	0.321	0.028	−0.155	−0.603	−0.896	0.240	−0.741	−0.506	0.394	−0.741	−0.679	−0.389
Breast Metastasis	−0.811	−1.472	−1.442	−2.105	−1.882	−1.103	−1.466	−2.072	−1.004	−1.751	−1.619	−1.062
Colon	4.498	4.554	4.581	4.490	4.484	4.611	4.585	4.504	4.460	4.563	4.397	4.570
Colon Cancer	3.081	3.243	3.371	2.970	2.937	3.302	3.129	2.876	3.203	3.224	3.009	3.263
Colon Metastasis	2.063	1.912	1.984	1.715	1.489	1.929	1.606	1.312	1.998	1.838	1.616	2.021
Lung	5.121	5.206	5.231	5.331	5.241	5.216	5.190	5.211	5.134	5.156	5.133	5.164
Lung Cancer	2.598	2.985	3.071	2.451	2.104	2.121	2.489	1.930	2.643	1.982	1.957	2.169
Adenocarcinoma	−0.058	−0.174	−0.444	−1.387	−1.148	−0.037	−0.404	−1.119	−0.094	−0.425	−0.267	−0.181
Squamous Cancer	−0.906	−1.644	−1.480	−1.882	−1.937	−1.759	−1.582	−2.208	−1.196	−1.754	−1.831	−1.150
hesc_1	−0.617	−0.608	−0.604	−0.557	−0.578	−0.604	−0.614	−0.591	−0.617	−0.607	−0.594	−0.612
hesc_2	−0.514	−0.503	−0.499	−0.437	−0.461	−0.500	−0.510	−0.474	−0.509	−0.496	−0.476	−0.509
hesc_3	−0.538	−0.529	−0.524	−0.473	−0.495	−0.525	−0.535	−0.507	−0.536	−0.526	−0.510	−0.533

	**13**	**14**	**15**	**16**	**17**	**18**	**19**	**20**	**21**	**22**	**X**	
Brain	5.135	4.999	5.084	4.922	4.950	5.094	4.992	4.877	4.959	4.851	5.486	
Glioma	0.571	0.448	0.595	0.203	0.721	0.524	−1.218	−0.031	0.071	0.301	−0.292	
Breast	4.666	4.416	4.495	4.162	4.220	4.523	4.064	4.176	4.178	4.005	4.751	
Breast Cancer	−0.966	−0.090	−0.017	−0.659	1.266	−0.681	1.128	−0.086	−0.329	0.454	−1.762	
Breast Metastasis	−1.950	−1.449	−0.931	−1.100	−0.663	−2.139	−0.809	−1.507	−1.789	−0.894	−2.325	
Colon	4.516	4.521	4.661	4.498	4.471	4.527	4.376	4.322	4.442	4.331	4.730	
Colon Cancer	2.758	3.132	3.264	2.979	3.322	2.693	3.061	2.599	2.728	3.113	2.768	
Colon Metastasis	1.397	1.895	2.027	1.779	2.412	1.678	1.907	1.374	1.376	2.384	0.748	
Lung	5.273	5.166	5.192	4.994	4.953	5.240	4.995	4.984	5.038	4.845	5.148	
Lung Cancer	2.791	2.730	2.792	2.341	2.422	2.975	1.017	1.672	1.926	3.056	1.209	
Adenocarcinoma	−1.526	−0.141	−1.634	−0.296	0.523	−1.369	0.240	−0.192	−0.335	0.385	−1.409	
Squamous Cancer	−1.452	−1.356	−1.296	−1.010	0.129	−2.115	−0.236	−1.797	−1.132	−0.329	−2.431	
hesc_1	−0.582	−0.606	−0.614	−0.651	−0.644	−0.589	−0.649	−0.612	−0.618	−0.666	−0.162	
hesc_2	−0.468	−0.498	−0.511	−0.547	−0.554	−0.474	−0.557	−0.494	−0.503	−0.571	−0.015	
hesc_3	−0.500	−0.526	−0.535	−0.572	−0.569	−0.507	−0.573	−0.528	−0.536	−0.588	−0.066	

^1^Energy values are given in $\mathrm{kJ}\times {\mathrm{mol}}^{-1}$. ^2^HESC: human embryonic stem cell values are provided as reference for an undifferentiated tissue. ^2^Healthy tissues and the corresponding cancer stages are shown grouped into an alternanting background color (light-gray and white).

**Figure 2:**
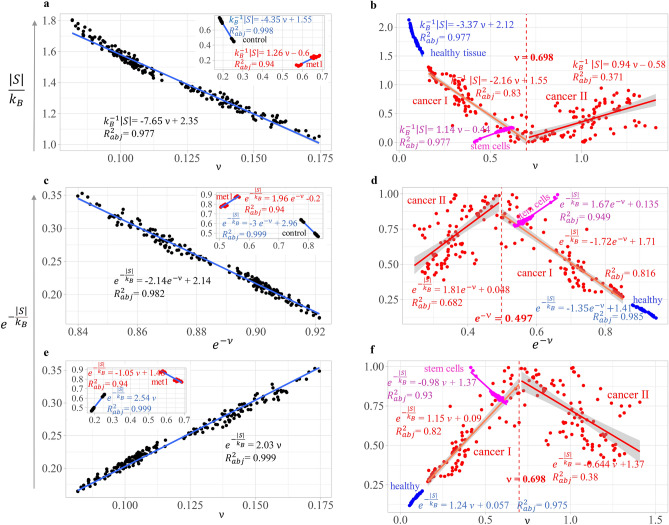
Evaluation of entropy fluctuations in experimental datasets from Arabidopsis and human patients with different types of cancer. Panels (**a**), (**c**), and (**e**) derive from Arabidopsis, panels (**b**), (**d**), and (**f**) from human data sets. The vertical dashed line in human panels indicates the cutpoint (estimated with K-means) splitting chromosomes (from all cancer types) into two groups (cancer 1 and cancer II). (**a**) and (**b**) regression analysis $-{k}_{B}^{-1}\left|S\right|$ versus the expected value (mean) $\nu =\langle \chi \rangle$ of the *J*-information-divergence $\chi$. (**c**) and (**d**), regression analysis ${e}^{-{k}_{B}^{-1}\left|S\right|}$ versus ${e}^{-\nu }$. (**e**) and (**f**), regression analysis ${e}^{-{k}_{B}^{-1}\left|S\right|}$ versus *ν*. Regression analyses in panel (**a**, **c**), and (**e**) were accomplished for datasets from Arabidopsis memory lines over six generations and the *met1* mutant (in the subplot). While regression analyses in panel (**b**, **d**), and (**f**) were accomplished for human datasets from patients with different types of cancer and tissue controls. Regression analyses support, up to experimental error, the regression model ${e}^{-{k}_{B}^{-1}\left|S\right|}=-\eta {e}^{-\upnu }+\eta$ or, equivalently, ${e}^{-{k}_{B}^{-1}\left|S\right|}=\eta \left(1-{e}^{-\upnu }\right)$. Only dysfunctional situations, such as the Arabidopsis *met1* mutant, human breast cancer, human metastasis (in red), or undifferentiated embryonic stem cells (hesc, in magenta), fail to conform to the linear model.

To test our second hypothesis, we first addressed the inference that in differentiated healthy tissue, the physical work accomplished by the methylation machinery must lead to a decrease in genome-wide methylation uncertainty, reflected in the values of (dimensionless) entropy $k_{B}^{ - 1} S$. This inference is supported by regression analysis $k_{B}^{ - 1} \left| S \right|$ versus ν accomplished in Arabidopsis and human datasets (Fig. 2a,b). K-means algorithm was applied to clustering chromosomes from all cancer types into the two groups denoted in Fig. 2 as: ‘cancer I’ and ‘cancer II’. Figure 2b shows that a subset of chromosomes from all cancer types appears to transition from a trend relatively close to healthy state (with negative slope, ‘cancer I’) to a weakly positive linear trend (‘cancer II’) in the direction of human embryonic stem cells (HESCs). A positive linear trend was also found in the Arabidopsis *met1* mutant (Fig. 2a).

These results provide us with an empirical estimation of the entropy fluctuations through the regression analysis $e^{{ - k_{B}^{ - 1} \,\left| S \right|}}$ versus $e^{ - \nu }$ (Fig. 2c,d), which leads to the equation:

27 $$e^{{ - k_{B}^{ - 1} \left| S \right|}} = \eta \left( {1 - e^{ - \nu } } \right)$$

where *η* is a proportionality constant. Or equivalently:

27a $$e^{{ - k_{B}^{ - 1} \left| S \right|}} = \eta - \eta {\kern 1pt} e^{ - \nu }$$

As shown in Eq. (27a) a negative value for model parameter $\eta$ (negative slope) is indicative of nonequilibrium feedback control. In an epigenetic context, nonequilibrium feedback control refers to the control accomplished by epigenetic regulatory machinery such as methyltransferases and demethylases. Figure 2c,d show that only the Arabidopsis *met1* mutant, chromosomes of all cancer types, and embryonic stem cells showed a positive slope $\eta >0$.

Equation (27) can be written as the quotient:

27b $$\frac{{e^{{ - k_{B}^{ - 1} \left| S \right|}} }}{{1 - e^{ - \nu } }} = \eta$$

which is another way to express the fluctuation theorem in a DNA methylation context. The model parameter $\eta$ characterizes the efficacy of feedback control ^38^.

Interpretation of Eq. (27) implies validity, up to experimental error, of the model:

28 $$e^{{ - k_{B}^{ - 1} \left| S \right|}} \cong \eta \nu$$

derived from Eq. (27) after using the approach $e^{ - \nu } = 1 - \nu + O\left( {\nu^{2} } \right)$. Thus, in the experimental dataset we would also find the most general model:

28a $$e^{{ - k_{B}^{ - 1} \left| S \right|}} \cong \eta \nu + c$$

where $c\in O\left({\nu }^{2}\right)$, which, within the limits of numerical error, approximates a constant not necessarily statistically significant. As shown in Fig. 2e,f, linear regression analysis confirms the statistical trend predicted by Eqs. (28) and (28a). With the exception of extreme conditions found in Arabidopsis mutant *met1* (red points, Fig. 2a,c,e subplots), cancer chromosomes from group II and stem cells (magenta points), the remainder of the data support Eqs. (27) and (28).

**Figure 3:**
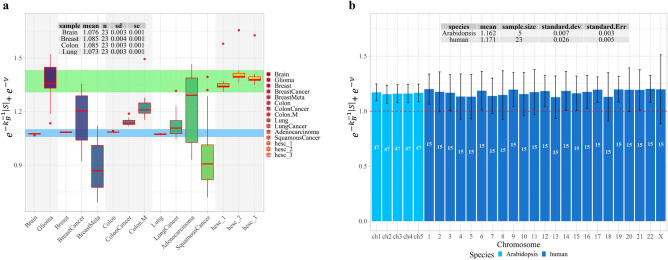
Fluctuations in the sum of Boltzmann’s factors $e^{{ - k_{B}^{ - 1} \left| S \right|}} + e^{ - \nu }$. (**a**), boxplot with sum of Boltzmann’s factors in human datasets. Healthy tissues and the corresponding cancer stages are shown grouped into an alternating background color (light-gray and white), e.g., lung adenocarcinoma and lung squamous cell cancer are grouped together with healthy lung tissue and typical lung cancer. The graphic shows that all cancer stages experience fluctuations from the expected range for all healthy tissues (light-blue band). Cancer cells fail to conform to $\langle {e}^{-{k}_{B}\left|S\right|}+{e}^{-\nu }\rangle =\eta$. The pluripotent embryonic stem cells, which can differentiate into other organ tissues, fluctuate at a range (light-green band) far from differentiated healthy tissues. Fluctuations close to stem cell range suggest the possibility of a cancer stem cell subpopulation at a given cancer development stage. (**b**) bar plot with estimations of the average of Boltzmann’s factors for entire sets of Arabidopsis and human samples. The number of individuals for each chromosome are given on each bar in white. The statistical summaries for the five Arabidopsis chromosomes and 23 human chromosomes are shown at the top. The error bars correspond to standard deviation estimates on each chromosome. Results indicate statistically nonsignificant differences for the means of Boltzmann’s factors sums estimated for Arabidopsis and human datasets, supporting Eq. (27). Data and R script to build this figure are given in the SI document.

Another way to arrive to Eq. (27a) is to consider the average of the sum of Boltzmann’s factors $e^{{ - k_{B}^{ - 1} \left| S \right|}}$ and $e^{ - \nu }$. Results suggest that the average sum of $e^{{ - k_{B}^{ - 1} \left| S \right|}} + e^{ - \nu }$ appears constant (Fig. 3). No statistical differences were found between the overall means of values from Arabidopsis (Fig. 3a) and humans (Fig. 3b), which leads us to postulate:

29 $$\left\langle {e^{{ - k_{B}^{ - 1} \left| S \right|}} + e^{ - \nu } } \right\rangle = \eta$$

where *η* has a value close to 1. Thus, we can write $\left\langle {e^{{ - k_{B}^{ - 1} \left| S \right|}} } \right\rangle = 1 - \left\langle {e^{ - \nu } } \right\rangle$ and, considering nonequilibrium feedback control^38^, $\left\langle {e^{{ - k_{B}^{ - 1} \left| S \right|}} } \right\rangle = \eta \left( {1 - \left\langle {e^{ - \nu } } \right\rangle } \right)$, which leads to Eq. (27). Small-range fluctuations are expected in normal healthy tissues, while notable fluctuation is expected in tissues/cells experiencing a disruption in methylation regulatory machinery. This last case is found in cancer cells shown in Fig. 3a, where the case of glioma departs substantially from healthy brain tissue and fluctuates at the level of stem cells. In biological terms, Eqs. (27–29) imply that the magnitude of genome-wide methylation changes originating in response to environmental change is restricted. Disease would presumably occur by large fluctuations outside the range of expected variation in healthy tissues.

## Discussion

We present a theoretical premise to account for DNA methylation variation behavior. Our results describe the information thermodynamics of cytosine methylation, extending well beyond the simple application of Eq. (9) as the null hypothesis required for methylation analysis. Results confirm that members of the generalized gamma probability distribution family, given by Eq. (6), quantitatively summarize the statistical physics underlying spontaneous methylation variation driven by random fluctuations. Parameters from Eq. (6) carry information about channel capacity of molecular machines^20,21^ that relates to Shannon’s capacity theorem.

Equation (9) can be interpreted as a conditional probability density distribution. The conditional probability interpretation of methylation (Eq. 13) assumes that the message remains constant in the control population and, under conditions of environmental variation or disease, changes in some subpopulation represented in treatment or patient datasets.

The conditional probability density ${\mathbb{P}}_{y} \left( \chi \right)$ indicates that if the recovered message at the receiving point is *y*, then ${\mathbb{P}}_{y} \left( \chi \right)$ will decline exponentially with the information divergence $\chi \left( {x,y} \right)$ between *y* and the message *x* produced by the source. Thus, if DNA methylation conforms to a communication system, then optimal coding of the methylation message is described in Eq. (9).

Methylation changes that support DNA thermal stability are expected to be present in highest frequency and with relatively small divergence values. Observed data from control populations show information divergence values $\chi \left(x,y\right)$ to be small, representing the housekeeping or background “noise” in the system. We expect that the probability $P\left(\chi \left(x,y\right)> {\chi }_{0.95}\right)$ to observe methylation background fluctuation with a value $\chi \left(x,y\right)$ greater than the 95% quantile ${\chi }_{0.95}$ is lesser than 0.05 ($P\left(\chi \left(x,y\right)> {\chi }_{0.95}\right)=1-P\left(\chi \left(x,y\right)\le {\chi }_{0.95}\right)$). In other words, Eq. (9) can be applied as null hypothesis in a signal detection-based approach to discriminate the methylation regulatory signal (expected with values $\chi \left(x,y\right)> {\chi }_{0.95}$) from methylation background^9,32^.

The methylation message is presumably encoded within the mechanical properties of the DNA molecule^1,2^. For example, flexibility or rigidity of the DNA double helix is required for regulating nucleosome folding and transcription factor (TF) binding to DNA sequence motifs^39,40^. Depending on DNA sequence context, the addition or removal of methyl groups to cytosine bases is predicted to alter these local physical properties^1,2^.

Gibb entropy and Helmholtz free energy, given by Eqs. (17) and (23), suggest a substantial distinction between classical statistical mechanics and statistical biophysics of the methylation process by considering the entropy contribution from the molecular machine (enzyme) through conformational changes, which is expressed in the term $\phi \left( {\alpha ,\delta } \right)$  from Eq. (17). Application of Eqs. (17) and (23) to experimental datasets can provide important biological insights. Results shown in Table 1 indicate that, as a thermodynamic state variable, the entropy given by Eq. (17) estimates the state of the methylation system consistent with phenotypic observations. The epigenetic memory lines in Arabidopsis produced an incremental effect on information loss observed from *nm1* to *mm3*. A much greater difference in energy (−2228.45 $\mathrm{J}\times {\mathrm{K}}^{-1}\times {\mathrm{mol}}^{-1}$) was observed between *met1* mutant and its corresponding experimental control, where the minus sign “-” indicates that the transformation was energetically favorable ($\Delta \Delta F < 0$) and that a loss of information ($I_{m} < 0$) occurred in this transformation (Eq. (26a)). Thus, the *met1* mutant, which undergoes a genome-wide loss in CG methylation^41^, provides a reference for extreme methylation change and information loss (Table 1).

Results presented in Tables 2 and 3 are biologically intuitive when considering the transformation of a pluripotent embryonic stem cell to a differentiated cell. From ovule to embryo to multicellular development involves continuous increase in order, translated to net gain of information^42,43^. We suggest that this phenomenon is reflected in methylome features.

Our data indicate that transformation of normal cells to cancer cells leads to an increase in entropy and, consequently, a loss of information $\Delta S = S_{healthy\;cells} - S_{cancer\;cells} < 0$ ^26^ (${I}_{m}<0$). Biological evidence similarly suggests that a loss of information from the original tissue occurs when cancer stem cells, a sub-population from within the tumor mass, derive from cancer cells^44,45^. Jointly, results from Tables 1 and 2 are in agreement with these known effects.

Fluctuation constraints revealed by Eqs. (27) to (29) are concerned with preserving the best coding and fidelity of the methylation message at receiver point, permitting sufficient variation of methylation signal to ensure organismal adaptation to environmental change. This concept is supported by the results obtained with the extreme scenarios shown for Arabidopsis mutant *met1*, cancer samples, and stem cells, where outcomes do not hold to models given in Eqs. (27) to (29). The *met1* mutation leads to an almost complete loss of CG gene-body methylation in Arabidopsis and a substantial ectopic CHG and CHH hypermethylation at genes and transposable elements^46^. The methylation reprogramming induced by cancer cells is also well documented^32,47^ and the massive loss of information is supported by the results shown in Table 2.

The case of embryonic stem cells is different from *met1* mutant and cancer cells. DNA methylation is not necessarily required in embryonic stem cells. Even when CG methylation is completely lost by combined knockout of three mammalian DNA methyltransferases Dnmt1, Dnmt3a, and Dnmt3b, there is a minimal change in phenotype in undifferentiated stem cells^48^.

The experimental finding of Eqs. (27) to (29), as applied to methylome datasets from human and Arabidopsis chromosomes, may be informative about the DNA methylation process and potential influence of methylation in system buffering. Equation (27) predicts limits in the system’s capacity to confront and minimize the effect of random entropy fluctuations. As suggested in Fig. 2, surpassing these limits could reflect system breakdown ^49–51^.

The connection with Shannon’s communication theory reveals a future avenue for application of discrete-state kinetics derived from a Markov model^29^ of the information source. A discrete-kinetic approach from the implicit Markov model of the source, and the evolution of such an epigenetic process, can be studied through the corresponding master equations that obey Chapman-Kolmogorov equations. Existence of epigenomic states is not only evident for the observable individual disease and heathy conditions, but also across the aging process^52^.

An intricate balance is expected for most epigenetic processes, which can be reversed^53^. That is, unlike DNA mutations, DNA methylation changes and consequent epigenetic alterations are, at least theoretically, reversible^6^. Thus, we can study the epigenomic process across organismal ontogeny as a stationary and ergodic Markov process.

As noted by Gorban^54^,^“^ “the only difference between the general first order (chemical) kinetics and master equation for the probability distribution is in the balance conditions: the sum of probabilities should be 1, whereas the sum of variables (concentrations) for the general first order kinetics may be any positive number*.”* From this perspective, the methylation regulatory signal, and associated epigenomic processes, reflects a system transitioning between possible stationary states in which an organism must constantly adapt to new environmental conditions. Development of this modeling is beyond the scope of our current study.

The primary goal of this study was to establish a theoretical basis for understanding DNA methylation behavior, but the practical outcomes of entropy estimates suggest that our results may have important implications for early diagnostics and assessing change in organismal state. Results suggest that information loss (entropy increments) and, consequently, DNA methylation reprogramming characterize cancer progression, suggesting that epigenetic mechanisms might be influential in cancer metastasis^55,56^. Our results also suggest that detection of early disease development stages on the basis of physical-informational chromosome states would be feasible.

## Materials and methods

### Biological experimental datasets

The Arabidopsis *thaliana* methylome datasets (with results reported in Table 1) from bisulfite sequencing of *msh1* memory and non-memory (normal looking) sibling plants with isogenic Col-0 wild-type control in Arabidopsis were downloaded from the Gene Expression Omnibus (GEO) Series GSE129303a and GSE118874.

The methylome datasets for *met1* mutant and corresponding wildtype were taken from the GEO Series GSE122394. The fastq files from Arabidopsis methylome *met1* mutant and corresponding wildtype datasets were downloaded from the European Nucleotide Archive (ENA, https://www.ebi.ac.uk/ena/browser/home). The raw read counts for *met1* methylated and non-methylated cytosines for further methylation analysis were obtained as follows: Raw sequencing reads were quality-controlled with FastQC (version 0.11.5), trimmed with TrimGalore! (version 0.4.1) and Cutadapt (version 1.15), then aligned to the TAIR10 reference genome using Bismark (version 0.19.0) with bowtie2 (version 2.3.3.1). The *deduplicate_bismark* function in Bismark with default parameters was used to remove duplicated reads and reads with coverage greater than 500 were removed to control PCR bias. Methylated Cs (COV files) were acquired from Bismark methylation extractor with default parameters.

The cancer and healthy tissues controls (Table 2) were downloaded from the GEO Series GSE52271. Blood B-cells CD19 (GSM1279518) was used as reference in the computation of information divergences *J*-divergences (*JD*). The Bi-seq dataset of Naive Human Embryonic Pluripotent Stem Cells have GEO accessions: GSM2041690, GSM2041691, and GSM2041692.

A more detailed description of these datasets is given in SI B.1.

### Computational tools and statistical analysis

The estimations of J-divergences, the best nonlinear fitted model to member of the generalized gamma distribution (Eqs. 9 and 11), Gibb entropy, and Helmholtz free energy were accomplished using functions from MethylIT R package (version 0.3.2.4): *gibb_entropy* and *helmholtz_free_energy*, respectively (https://genomaths.github.io/methylit/). The estimations of the Boltzmann's factors shown in Figs. 2 and 3 were accomplished using MethylIT function *boltzman_factor*. All R scripts for Tables 1, 2, 3 results are available as SI.

The group comparison shown in Table 1 was accomplished in the *lme4* R package (version 1.1–27.1) applying a linear mixed model with chromosome random effects with formula: $entropy=group+\left(1|chromosome\right)$.

## Supplementary Information

Supplementary Information.

## Data Availability

All the methylome datasets and software used in this work are publicly available at GitHub: https://github.com/genomaths/MethylIT (version 0.3.2.4). As specified in Material and Methods section (and in the SI), all methylome raw data used in the scripts has been downloaded from GEO or ENA databases. Intermediate datasets used in the downstream analysis to support the conclusions of this report are available on GitLab at Penn State at https://git.psu.edu/genomath/datasets. R script to accomplish all the computations are included within SI. So, readers can reproduce all the computations accomplished in this study.
