# Supplementary material for: On the thermodynamics of DNA methylation process
Source: Sci Rep. 2023 Jun 1;13:8914. doi: 10.1038/s41598-023-35166-9 (PMC10235097; doi:10.1038/s41598-023-35166-9)
Supplement: Supplementary file 1 — Supplementary Information. [file 41598_2023_35166_MOESM1_ESM.pdf]

Supplementary Information for

On the Thermodynamics of DNA Methylation Process

Robersy Sanchez[1*], Sally A. Mackenzie[2*]

[1]Departments of Biology, The Pennsylvania State University, University Park, PA 16802
ORCID: https://orcid.org/0000-0002-5246-1453

[2]Departments of Biology and Plant Science, The Pennsylvania State University, University Park, PA 16802.
ORCID: https://orcid.org/0000-0003-2077-5607

*Correspondence to:
RS email: rus547@psu.edu
SAM email: sam795@psu.edu

Table of Contents

On the Thermodynamics of DNA Methylation Process 1
A. Derivation of the probability density function of the methylation background 1
B. Properties of the Generalized Gamma (GG) Distribution 3
B.2 Moments of the Generalized Gamma Distribution 4
B.1 Gibb's Entropy 5
C. Material and Methods used to compute results presented in Table 1 6
C.1. Methylome data 6
C.1.1 Human dataset 6
C.1.2 Arabidopsis thaliana datatset 6
C.2. Methylation analysis 7
D. R Script for the Analysis of cancer data set 7
D.1 Reading data sets 7
D.2 Hellinger divergence 8
D.3 Nonlinear regression for JD 9
D.4 Gibb Entropy 12
D.5 Helmholtz free energy 14
E. R Script for the Analysis of Arabidopsis data set 15
E.1 Download files from GEO 15

E.2 Reading datasets 16
E.3 The reference sample 16
E.4 J-Divergence 16
E.5 Estimation of the best fitted probability distribution model 17
E.6 Thermodynamic state variables 17
F. R Script for the Analysis of Entropy fluctuations 19
F.1 Arabidopsis dataset 19
F.2 Plotting linear regression for Arabidopsis dataset 21
F.2.1 The graphic $\frac{|S|}{k_B}$ vs ν 22
F2.2 The graphic $e^{-\frac{|S|}{k_B}}$ vs ν 23
F2.3 The graphic $e^{-\frac{|S|}{k_B}}$ vs $e^{-\nu}$ 25
F.3 Cancer dataset 26
F.3.1 All tissues 26
F.3.2 Healthy tissue 27
F.3.3 Cancer tissue 27
F.4 Plotting linear regression for Cancer dataset 31
F.4.1 The graphic $\frac{|S|}{k_B}$ vs ν 31
F4.2 The graphic $e^{-\frac{|S|}{k_B}}$ vs ν 32
F4.3 The graphic $e^{-\frac{|S|}{k_B}}$ vs $e^{-\nu}$ 34
F.5 Bar and box plots 35
F5.1 Summarize by species 35
F5.2 The barplot 35
F5.3 The boxplot 36
G Group Differences in Arabidopsis Memory line based Entropy 37
G.1 Linear and generalized linear models for memory line 1rt generation 41
G.2 Linear and generalized linear models for non-memory line 42
G.3 Linear and generalized linear models for memory line 3rd generation 43
G.4 Linear and nonlinear models for met1 44
G.5 Linear model with fixed effects for met1 45
G.6 Result table 46

A. Derivation of the probability density function of the methylation background

We searched for PDFs from the generalized gamma (GG) distribution family given by Eq. 2 (main text). The following assumptions must hold:

a) Let N_i be the number of times that an amount of energy E in the interval $\left[E_{i-1}, E_i\right)$ is dissipated.

b) Let us consider a number k of such events: $N_1, N_2, ..., N_k$, where $\sum_i^k N_i = N$.

c) Let p_i be the probability that an amount of energy E is dissipated in the interval $\left[E_{i-1}, E_i\right)$, where $\sum_i^k p_i = 1$.

The number of ways a set $\left\{N_i \mid i = 0,..,k\right\}$ can be realized in a sequence of length N is given by the multinomial coefficient: $\frac{N!}{N_1! \times N_2! \times ... \times N_k!}$ and the probability of each sequence of events is: $p_1^{N_1} \times p_2^{N_2} \times ... \times p_k^{N_k}$. Hence the probability that N distinguishable methylations events result in N_1 outcomes with energy dissipated in the interval $\left[E_0, E_1\right)$, N_2 outcomes with energy dissipated in the interval $\left[E_1, E_2\right)$, … , and N_k outcomes in the interval $\left[E_{k-1}, E_k\right)$ is given by the multinomial distribution:

$$\boldsymbol{P}\left(N_1, \ldots, N_k, N, p_1, \ldots, p_k\right) = \frac{N!}{\prod_{i=1}^{k} N_i!} \prod_{i=1}^{k} p_i^{N_i} \tag{S1}$$

Thus, the *most probable* distribution of methylation states in the system, a DNA molecule, is determined by the set of values $\left\{N_i\right\}$ and $\left\{p_i\right\}$, and the constant N, which in the current case is the number of cytosine sites in the DNA molecule. The continuous action of the Second Law tends to maximize the Boltzmann entropy inside each cell, which, in turns, leads to the most probable density of methylation states. Hence, we will search for the values $\hat{N}_i$ of N_i that maximize the probability $\boldsymbol{P}$ for a fixed set of probability values $\left\{p_i\right\}$. In particular, requirements 1 (Eq. 4) and 2 (Eq. 5, given in the main text) are imposed upon the p_i and N_i, which for the sake of better comprehension are repeated here:

1) probabilities p_i are proportional to a specific power of the energies E_i:

$$p_i = \left(\frac{E_i}{E^0}\right)^{\nu-1} \tag{4}$$

where E^0 stands for the energy dissipated with probability 1.

2) for each choice of α the following sum is a positive constant:

$$\sum_{i=1}^{k} N_i E_i^{\alpha} = E_{const} \tag{5}$$

where $E > 0$; N_i 's are assumed large numbers.

This problem is solved by writing down the Lagrangian $\mathcal{L}\left(N_1,\ldots,N_k,\beta,\lambda\right)$, which consists of a linear combination of the logarithm of ***P*** with the relevant constraints,:

$$\mathcal{L} = \log N! + \sum_{i=1}^{k} N_i \left(\nu-1\right)\log\frac{E_i}{E^0} - \sum_{i=1}^{k} \log N_i! - \frac{1}{\lambda}\left(\sum_{i=1}^{k} N_i\, E_i^{\alpha} - E_{const}\right) - \mu\left(\sum_{i=1}^{k} N_i - N\right) \tag{S2}$$

After approximating the factorial for large N using Stirling's formula, setting the derivatives $\frac{\partial\mathcal{L}}{\partial N_i} = 0$ ($i = 0,\ldots,k$) yields the equations:

$$\left(\nu-1\right)\log\frac{E_i}{E^0} - \log N_i - \frac{E_i^{\alpha}}{\lambda} - \mu = 0 \tag{S3}$$

Or

$$\log N_i = \log\left(e^{-\mu}\left(\frac{E_i}{E^0}\right)^{(\nu-1)} e^{\frac{E_i^{\alpha}}{\lambda}}\right) \tag{S3a}$$

Which, after setting $\eta = e^{-\mu}\left(\frac{1}{E^0}\right)^{\nu-1}$ (S4) , leads to the expressions:

$$\hat{N}_i = \eta E_i^{\nu-1} e^{-\frac{E_i^{\alpha}}{\lambda}} \tag{S5}$$

And

$$N = \eta \sum_{i}^{k} E_i^{\nu-1} e^{-\frac{E_i^{\alpha}}{\lambda}} \tag{S6}$$

The last equation permits us to update the discrete probability distribution π_i under assumptions 1 and 2:

$$\pi_i = \frac{\hat{N}_i}{N} = \frac{E_i^{\nu-1} e^{-\frac{E_i^{\alpha}}{\lambda}}}{\sum_{i}^{k} E_i^{\nu-1} e^{-\frac{E_i^{\alpha}}{\lambda}}} \tag{S7}$$

That is, $\frac{\hat{N}_i}{N}$ can be interpreted as discrete probability distribution associated with the events of energy dissipation under the system constraints 1 and 2. Alternatively, we can write Eq. S7 as:

$$\frac{\hat{N}_i}{N} = \Delta E\left[\sum\nolimits_{i=0}^{k} E_i^{\nu-1} e^{-\frac{E_i^{\alpha}}{\lambda}} \Delta E\right]^{-1} E_i^{\nu-1} e^{-\frac{E_i^{\alpha}}{\lambda}} \tag{S8}$$

Which after set $k \to \infty$ the size of the interval $\Delta E \to 0$ and the sum in the bracketed coefficient can be replaced by a definite integral:

$$\int_0^{\infty} E_i^{\nu-1} e^{-\frac{E_i^{\alpha}}{\lambda}} dE = \frac{\lambda^{\frac{\nu}{\alpha}}\, \Gamma(\nu/\alpha)}{\alpha} \tag{S9}$$

Thus, Eq. S8 becomes:

$$\frac{\hat{N}_i}{N} = \Delta E\left[\frac{\alpha}{\lambda^{\nu/\alpha}\, \Gamma(\nu/\alpha)} E_i^{\nu-1} e^{-\frac{E_i^{\alpha}}{\lambda}}\right] \tag{S10}$$

Assuming that the energies E_i dissipated to reach the states i of the system are virtually a continuum, the right term between brackets becomes a probability density function:

$$f\left(E|\alpha,\beta,\nu\right) = \frac{\alpha}{\lambda^{\nu/\alpha}\Gamma(\nu/\alpha)} E^{\nu-1} e^{-\frac{E^{\alpha}}{\lambda}} \tag{S11}$$

Which after set $\lambda = \beta^{\alpha}$ leads to the generalized gamma distribution (GG) with parametrization given by Stacy (29):

$$f\left(E|\alpha,\beta,\nu\right) = \frac{\alpha}{\beta^{\nu}\, \Gamma(\nu/\alpha)} E^{\,\nu-1} e^{-\left(\frac{E}{\beta}\right)^{\alpha}} \tag{S12}$$

The form commonly used in practice is obtained by the parametrization: $\delta = \nu/\alpha$, which give rise to Eq. 9 from the main text:

$$f\left(E|\alpha,\beta,\delta\right) = \frac{\alpha}{\beta\,\Gamma(\delta)}\left(\frac{E}{\beta}\right)^{\alpha\delta-1} e^{-\left(\frac{E}{\beta}\right)^{\alpha}} \tag{9}$$

B. Properties of the Generalized Gamma (GG) Distribution

The properties of the GG distribution can easily derived using Wolfram Mathematica (https://reference.wolfram.com/language/ref/GammaDistribution.html).

The cumulative distribution function (CDF) of the GG distribution is given by the expression:

$$F\left(E\middle|\alpha,\mu,\beta,\delta\right)=\begin{cases}\gamma\left(\delta,\left(\frac{E-\mu}{\beta}\right)^{\alpha}\right) & \delta>0 \text{ and } E>\mu>0\\ 1-\gamma\left(\delta,\left(\frac{E-\mu}{\beta}\right)^{\alpha}\right) & \delta\le 0 \text{ and } E>\mu>0\end{cases} \tag{S13}$$

Where $\gamma(.)$ denotes the lower incomplete gamma function.

The kurtosis of the GG distribution is:

$$\frac{-3\Gamma\left(\frac{1}{\alpha}+\delta\right)^4+6\Gamma(\delta)\Gamma\left(\frac{1}{\alpha}+\delta\right)^2\Gamma\left(\frac{2}{\alpha}+\delta\right)-4\Gamma(\delta)^2\Gamma\left(\frac{1}{\alpha}+\delta\right)\Gamma\left(\frac{3}{\alpha}+\delta\right)+\Gamma(\delta)^3\Gamma\left(\frac{4}{\alpha}+\delta\right)}{\left(\Gamma\left(\frac{1}{\alpha}+\delta\right)^2-\Gamma(\delta)\Gamma\left(\frac{2}{\alpha}+\delta\right)\right)^2} \tag{S14}$$

The Skewness is:

$$\frac{2\Gamma\left(\frac{1}{\alpha}+\delta\right)^3-3\Gamma(\delta)\Gamma\left(\frac{1}{\alpha}+\delta\right)\Gamma\left(\frac{2}{\alpha}+\delta\right)+\Gamma(\delta)^2\Gamma\left(\frac{3}{\alpha}+\delta\right)}{\left(-\Gamma\left(\frac{1}{\alpha}+\delta\right)^2+\Gamma(\delta)\Gamma\left(\frac{2}{\alpha}+\delta\right)\right)^{3/2}} \tag{S15}$$

B.2 Moments of the Generalized Gamma Distribution

The 1st, 2nd, 3rd, and 4th moments of the GG distribution with location parameter μ are, respectively:

$\frac{\mu\Gamma(\delta)+\beta\Gamma\left(\frac{1}{\alpha}+\delta\right)}{\Gamma(\delta)}$
$\frac{\mu^2\Gamma(\delta)+2\beta\mu\Gamma\left(\frac{1}{\alpha}+\delta\right)+\beta^2\Gamma\left(\frac{2}{\alpha}+\delta\right)}{\Gamma(\delta)}$
$\frac{\mu^3\Gamma(\delta)+3\beta\mu^2\Gamma\left(\frac{1}{\alpha}+\delta\right)+3\beta^2\mu\Gamma\left(\frac{2}{\alpha}+\delta\right)+\beta^3\Gamma\left(\frac{3}{\alpha}+\delta\right)}{\Gamma(\delta)}$
$\frac{\mu^4\Gamma(\delta)+4\beta\mu^3\Gamma\left(\frac{1}{\alpha}+\delta\right)+6\beta^2\mu^2\Gamma\left(\frac{2}{\alpha}+\delta\right)+4\beta^3\mu\Gamma\left(\frac{3}{\alpha}+\delta\right)+\beta^4\Gamma\left(\frac{4}{\alpha}+\delta\right)}{\Gamma(\delta)}$

B.1 Gibb’s Entropy

To deduce the Gibb’s entropy as given in Eq 13 (from the main text), we used the definition of entropy, as the expectation of the logarithm of GG distribution, then expanded the logarithm into a sum and replaced the expectation by its values. That is, the logarithm of Eq. 9 is:

$$\ln\left(\frac{\alpha}{\beta\,\Gamma(\delta)}\left(\frac{E}{\beta}\right)^{\alpha\delta-1} e^{-\left(\frac{E}{\beta}\right)^{\alpha}}\right)=\ln\frac{\alpha}{\beta\,\Gamma(\delta)}+(\alpha\delta-1)\ln\frac{E}{\beta}-\left(\frac{E}{\beta}\right)^{\alpha}$$

Then, the Gibb’s entropy is given by the expression:

$$\Delta S=-k\int_0^{\infty} f\left(E\middle|\alpha,\beta,\delta\right)\ln\left(\frac{\alpha}{\beta\,\Gamma(\delta)}\left(\frac{E}{\beta}\right)^{\alpha\delta-1} e^{-\left(\frac{E}{\beta}\right)^{\alpha}}\right)dE=$$

$$-k\int_0^{\infty} f\left(E\middle|\alpha,\beta,\delta\right)\left(\ln\frac{\alpha}{\beta\,\Gamma(\delta)}+(\alpha\delta-1)\ln\frac{E}{\beta}-\left(\frac{E}{\beta}\right)^{\alpha}\right)dE$$

The expected value of $\left(\frac{E}{"\beta}"\right)^{\alpha}$:

$$\int_0^{\infty}\frac{\alpha}{\beta\,\Gamma(\delta)}\left(\frac{E}{\beta}\right)^{\alpha\delta-1} e^{-\left(\frac{E}{\beta}\right)^{\alpha}}\left(\frac{E}{\beta}\right)^{\alpha} dE=\delta$$

The expected values of $\ln\left(\frac{E}{\beta}\right)^{\alpha\delta-1}$:

$$\int_0^{\infty}\frac{\alpha}{\beta\,\Gamma(\delta)}\left(\frac{E}{\beta}\right)^{\alpha\delta-1} e^{-\left(\frac{E}{\beta}\right)^{\alpha}}\ln\left(\frac{E}{\beta}\right)^{\alpha\delta-1} dE=\frac{(\alpha\delta-1)\psi(\delta)}{\alpha}$$

where is $\psi(\delta)=\frac{d\ln\Gamma(\delta)}{d\delta}$ stands for the digamma function.

Thus, the Gibb’s entropy is:

$$\Delta S=-k\left(\ln\frac{\alpha}{\beta\,\Gamma(\delta)}+\left(\delta-\frac{1}{\alpha}\right)\psi(\delta)-\delta\right)$$

Which basically is Eq 15 from the main text:

$$S=k_B\left(\ln\frac{\beta\,\Gamma(\delta)}{\alpha}+\psi(\delta)\left(\frac{1}{\alpha}-\delta\right)+\delta\right)\qquad(15)$$

C. Material and Methods used to compute results presented in Table 1

C.1. Methylome data

C.1.1 Human dataset

All the methylome data sets were taken from Gene Expression Omnibus (GEO) data base.

a. Bi-seq data sets from Naive Human Pluripotent Cells has GEO accession: GSM2041690, GSM2041691, and GSM2041692.

b. Human cancer types and corresponding normal tissue with GEO accessions: GSE52271 and GSE56763.

1. Brain white matter (GSM1279516)
2. Brain Glioma (GSM1279532)
3. Breast normal (GSM1279517)
4. Breast cancer (GSM1279514)
5. Breast metastasis (GSM1279513)
6. Colon normal (GSM1279519)
7. Colon cancer (GSM1279521)
8. Colon metastasis (GSM1279520)
9. Lung normal (GSM1279527)
10. Lung cancer (GSM1279524)
11. Blood B-cells CD19, normal (GSM1279518)

The Blood B-cells CD19 sample was used as reference in the computation of information divergences: Hellinger (*HD*) and *J*-divergences (*JD*).

C.1.2 Arabidopsis thaliana dataset

The *Arabidopsis thaliana* methylome datasets of *msh1* memory and non-memory (normal phenotype) sibling plants were derived from the *msh1* mutant. As described in reference (35), an *MSH1*-RNAi transgene positive plant was self-pollinated and the transgene was segregated in the subsequent

generation. Of transgene null plants, 20% plants displayed delay in flowering, smaller size, and pale green leaves, termed 'memory' phenotype. Memory plants were self-pollinated for six generations and plants from each generation were DNA-sampled for bisulfite sequencing and methylome analysis. The dataset generation 2-6 can be accessed with GEO accession number GSE129303 and GSE118874.

The RData files with *J*-information-divergence and with the best fitted models and corresponding goodness-of-fit used in all the computation reported in the main text are available at PSU GitLab: https://git.psu.edu/genomath/datasets.

C.2. Methylation analysis

Methylation analysis was accomplished with MethylT R package (0.3.2.2), which is available at GitHub: https://github.com/genomaths/MethylIT. The R scripts with the methylation analysis are available below, in section D. MethylIT tutorials and function descriptions are available at https://genomaths.github.io/methylit/. The RData with the main results derived from the R scripts pipeline are available/public at PSU GitLab: https://git.psu.edu/genomath/datasets.

The best fitted PDF model parameters used to estimate Gibb entropy and Helmholtz free energy for each chromosome can be retrieved as shown in section D3.

D. R Script for the Analysis of cancer data set

This data set was downloaded from the Gene Expression Omnibus (GEO) to a local folder and read into R

D.1 Reading data sets

```
library(MethylIT)


folder <- "~/Cancer/GSE52271/"
files = list.files(path = folder, pattern = "txt.gz")
files = paste0(folder, files)

folder <- "/data/HumanMethy/Cancer/GSE56763/"
files = c(files, paste0(folder, list.files(path = folder,  pattern = "txt.gz")))

# [1] "~/Cancer/GSE52271/GSM1279516_CpGcontext.Brain_W.txt.gz"
# [2] "~/Cancer/GSE52271/GSM1279517_CpGcontext.Breast.txt.gz"
# [3] "~/Cancer/GSE52271/GSM1279518_CpGcontext.CD19.txt.gz"
# [4] "~/Cancer/GSE52271/GSM1279519_CpGcontext.Colon.txt.gz"
```

```
# [5] "~/Cancer/GSE52271/GSM1279520_CpGcontext.Colon_M.txt.gz"
# [6] "~/Cancer/GSE52271/GSM1279521_CpGcontext.Colon_P.txt.gz"
# [7] "~/Cancer/GSE52271/GSM1279522_CpGcontext.H1437.txt.gz"
# [8] "~/Cancer/GSE52271/GSM1279523_CpGcontext.H157.txt.gz"
# [9] "~/Cancer/GSE52271/GSM1279524_CpGcontext.H1672.txt.gz"
# [10] "~/Cancer/GSE52271/GSM1279527_CpGcontext.Lung.txt.gz"
# [11] "~/Cancer/GSE52271/GSM1279532_CpGcontext.U87MG.txt.gz"
# [12] "~/Cancer/GSE56763/GSM1279513_CpGcontext.468LN.txt.gz"
# [13] "~/Cancer/GSE56763/GSM1279514_CpGcontext.468PT.txt.gz"

sn = c("Brain", "Breast", "CD19", "Colon", "Colon.M", "ColonCancer",
       "Adenocarcinoma", "SquamousCancer", "LungCancer", "Lung", "Glioma",
       "BreastMeta", "BreastCancer")

LR. = readCounts2GRangesList(filenames = files, sample.id = sn,
                             columns = c(seqnames = 1, start = 2,
                                         mC = 3, uC = 4 ),
                             pattern = "^[^MY]")

LR <- c(LR, LR.)
names(LR[-6])

save(LR, file = "~/Cancer/RData/LR_cancer.R")
```

D.2 Hellinger divergence

Estimation of Hellinger divergence

```
load("~/Cancer/RData/LR_cancer.R")

HD = estimateDivergence(ref = LR$CD19,
                        indiv = LR[-6],
                        Bayesian = TRUE,
                        min.coverage = 8,
                        high.coverage = 500,
                        min.meth = 3,
                        min.umeth = 0,
                        percentile = 0.999,
                        JD = TRUE,
                        num.cores = 60L,
                        verbose = FALSE)

save(HD, file = "~/Cancer/RData/hd_cancer.RData")
```

For the sake of reducing computational work, we are interested only in J divergences from chromosomes, 7, 9, 17, and 22.

```
chrs <- c("7", "9", "17", "22")
nams <- names(HD)
# [1] "hesc_1"         "hesc_2"       "hesc_3"         "Brain"          "Breast"
# [6] "Colon"          "Colon.M"      "ColonCancer"    "Adenocarcinoma" "SquamousCancer"
# [11] "LungCancer"     "Lung"          "Glioma"          "BreastMeta"      "BreastCancer"

jd_chr <- function(jd, chr) lapply(jd, function(x) {
  seqlevels(x, pruning.mode = "coarse") <- chr
  x <- x[, 10]
  return(x)
  }, keep.attr = TRUE
)
```

```
jd <- vector("list", 4)
for (k in seq_along(chrs)) {

  cat("\n *** ========== Processing chromosome: ", chrs[k], " ...\n")
  jd[[ k ]] <- jd_chr(jd = HD, chr = chrs[ k ])
}

names(jd) <- paste0("chr", chrs)

save(jd, file = "~/Cancer/RData/jd_cancer_datasets.RData", compress = "xz")
```

D.3 Nonlinear regression for JD

The last file can be downloaded from GitLab using the following script:

```
## ===== Download methylation data from GitLab ========
url <- paste0("https://git.psu.edu/genomath/datasets/-/raw/",
              "main/cancer_data/jd_cancer_datasets.RData")
temp <- tempfile(fileext = ".RData")
download.file(url = url, destfile = temp)
load(temp)
file.remove(temp); rm(temp, url)

}
```

The estimation of the best fitted model requires, depending on the computer power, for considerable computation time. We do not need to apply the next ‘loop’:

```
chrs <- c("7", "9", "17", "22")
d <- c( "Weibull2P", "Weibull3P",
        "Gamma2P", "Gamma3P", "GGamma3P")

jd_chr <- function(hd, chr) lapply(hd, function(x) {
  seqlevels(x, pruning.mode = "coarse") <- chr
  return(x)
}, keep.attr = TRUE
)

j <- 1
gof <- vector("list", 4)
for (k in chrs) {

  jd <- jd_chr(hd = HD, chr = k)

  cat("\n *** ========== Processing chromosome: ", k, " ...\n")
  gof <- gofReport(
                HD = jd,
                model = d,
                column = 10,
                output = "best.model",
                confl_model = TRUE,
                alt_models = TRUE,
                um.cores = 40L,
                verbose = TRUE
  )
  save(gof, file = paste0(folder,"jdiv_gof_per-chr", k,"_gwf_cancer_05-16-22.RData"))
  gofs[[j]] <- gof
  j = j + 1
```

```
}
names(gofs) <- paste0("chr", chrs)
```

A way to reduce the computational time can be to split the above computation in several different jobs.

```
## ===== Download methylation data from GitLab ========
url <- paste0("https://git.psu.edu/genomath/datasets/-/raw/",
            "main/cancer_data/gofs/script4pjob_split.R")

temp <- tempfile(fileext = ".R")
download.file(url = url, destfile = temp)

pjob_split(filename = temp,
           args = NULL,
           num.jobs = 4L,
           verbose = TRUE)

# file.remove(temp); rm(temp, url)
```

The R script '*script4pjob_split.R*' is passed to function 'pjob_splits', which returns a gof report for chromosomes: "7", "9", "17", and "22" in folder `"/home"`: `paste0("~/gof_jd_", chroms[i], ".RData")`. Notice that depending on the samples size, the distribution of parallel computation could take from seconds to 1-2 minutes.

The results from the nonlinear fit for all chromosomes can be downloaded from GitLab

```
## ===== Download methylation data from GitLab ========
url <- paste0("https://git.psu.edu/genomath/datasets/-/raw/",
              "main/cancer_data/gofs/jdiv_gof_per_chr_1-X__cancer_03-21-2023.RData")
temp <- tempfile(fileext = ".RData")
download.file(url = url, destfile = temp)
load(temp)
file.remove(temp); rm(temp, url)
#> [1] TRUE

gofs[[1]]
#> ProbDistrList object
#> -------
#> Summary GoF table (6 first rows from: x$stats)
#>          w2p_AIC w2p_R.Cross.val w3p_AIC w3p_R.Cross.val   g2p_AIC
#> hesc_1  -5889619       0.9920121     Inf               0  -5303789
#> hesc_2  -5920634       0.9920897     Inf               0  -5393333
#> hesc_3  -5884296       0.9919699     Inf               0  -5317527
#> Brain   -9967148       0.9993754     Inf               0  -8720896
#> Breast -15356304       0.9998766     Inf               0 -10478553
#> Colon  -16997188       0.9999729     Inf               0 -10546798
#>        g2p_R.Cross.val g3p_AIC g3p_R.Cross.val  gg3p_AIC gg3p_R.Cross.val
#> hesc_1       0.9870140     Inf               0  -9122345        0.9987446
#> hesc_2       0.9877016     Inf               0  -9335335        0.9989045
#> hesc_3       0.9871222     Inf               0  -9155473        0.9987695
#> Brain        0.9985995     Inf               0  -9951556        0.9993525
#> Breast       0.9984338     Inf               0 -16183433        0.9999151
#> Colon        0.9990071     Inf               0 -16957708        0.9999709
#>        bestModel
#> hesc_1      gg3p
#> hesc_2      gg3p
#> hesc_3      gg3p
#> Brain        w2p
#> Breast      gg3p
#> Colon        w2p
```

```
#> ...
#> -------
#> cdfMODELlist  object of length:  15
#> $hesc_1
#>          Estimate   Std. Error   t value Pr(>|t|))      Adj.R.Square
#> alpha 32.78779270 0.2402385275  136.4802         0 0.999998476103597
#> scale  1.25769335 0.0001411931 8907.6139         0
#> psi    0.02868718 0.0002105403  136.2550         0
#>                     rho       R.Cross.val               DEV               AIC
#> alpha 0.999998476097108 0.998744630938867 0.212630635607947 -9122345.24732909
#> scale
#> psi
#>                     BIC     COV.alpha     COV.scale       COV.psi COV.mu
#> alpha -9122295.99718835  5.771455e-02 -3.253028e-05 -5.057851e-05     NA
#> scale                   -3.253028e-05  1.993548e-08  2.846371e-08     NA
#> psi                     -5.057851e-05  2.846371e-08  4.432722e-08     NA
#>             N    model
#> alpha 1643823 GGamma3P
#> scale 1643823
#> psi   1643823
#>
#> ...
#> <14 more cdfMODEL elements>
#> -------
```

Just to ordering the models by type of cancer:

```
stage <- c(
        "Brain", "Glioma", "Breast",
        "BreastCancer", "BreastMeta", "Colon",
        "ColonCancer", "Colon.M",
        "Lung", "LungCancer",
        "Adenocarcinoma", "SquamousCancer",
        "hesc_1", "hesc_2", "hesc_3")

gofs <- lapply(gofs, function(x) {
    x$nlms <- x$nlms[stage]
    class(x$nlms) <- "cdfMODELlist"
    return(x)
}, keep.attr = TRUE)

nams <- paste0("chr", names(gofs))

bestfit <- lapply(seq_along(gofs), function(j) {
    nlms <- gofs[[j]]$nlms
    nms <- names(nlms)
    chr <- lapply(seq_along(gofs[[j]]$nlms), function(k) {
            m <- gofs[[j]]$nlms[[k]]
            cbind(m[, c(1:2)], RCV = round(as.numeric(m$R.Cross.val),3),
                AIC =  round(as.numeric(m$AIC)), model = m$model)
            })
    names(chr) <- nms
  do.call(rbind, chr)
})
names(bestfit) <- nams
bestfit$chr1
#>                             Estimate   Std. Error   RCV       AIC     model
#> Brain.shape              0.609012284 3.157552e-05 0.999  -9967148 Weibull2P
#> Brain.scale              0.045499250 2.443285e-06    NA        NA
#> Glioma.shape             0.449959806 2.987186e-05 0.999  -9316192 Weibull2P
#> Glioma.scale             0.278392930 2.770091e-05    NA        NA
```

```
#> Breast.alpha          0.474907352 4.072208e-05 1.000 -16183433  GGamma3P
#> Breast.scale          0.032665085 1.261942e-05    NA        NA
#> Breast.psi            1.260125246 1.775193e-04    NA        NA
#> BreastCancer.alpha    0.267796747 1.775177e-04 0.998 -10134135  GGamma3P
#> BreastCancer.scale    0.009100222 6.432492e-05    NA        NA
#> BreastCancer.psi      2.267961429 2.809435e-03    NA        NA
#> BreastMeta.alpha     22.260052394 2.012522e+00 0.997  -8987578  GGamma3P
#> BreastMeta.scale      4.567744246 1.026616e-02    NA        NA
#> BreastMeta.psi        0.013123173 1.186526e-03    NA        NA
#> Colon.shape           0.559407369 5.385569e-06 1.000 -16997188 Weibull2P
#> Colon.scale           0.056669588 6.334668e-07    NA        NA
#> ColonCancer.alpha     0.428083269 7.298609e-05 1.000 -14475771  GGamma3P
#> ColonCancer.scale     0.043693575 4.026960e-05    NA        NA
#> ColonCancer.psi       1.333108880 3.990829e-04    NA        NA
#> Colon.M.alpha         0.485673809 1.113183e-04 0.999 -12190711  GGamma3P
#> Colon.M.scale         0.139319612 1.319452e-04    NA        NA
#> Colon.M.psi           1.022261334 3.862188e-04    NA        NA
#> Lung.alpha            0.689680081 7.538683e-05 1.000 -13472488  GGamma3P
#> Lung.scale            0.066835005 1.702831e-05    NA        NA
#> Lung.psi              0.809405531 1.301653e-04    NA        NA
#> LungCancer.alpha      0.305767012 9.556865e-05 1.000 -11676412  GGamma3P
#> LungCancer.scale      0.009607601 2.661881e-05    NA        NA
#> LungCancer.psi        1.954238729 1.128058e-03    NA        NA
#> Adenocarcinoma.alpha  1.253561436 8.449787e-04 0.996  -7747318  GGamma3P
#> Adenocarcinoma.scale  3.059737438 1.042037e-03    NA        NA
#> Adenocarcinoma.psi    0.241456490 1.895214e-04    NA        NA
#> SquamousCancer.alpha 49.757767411 1.260034e+00 0.996  -7816286  GGamma3P
#> SquamousCancer.scale  4.309435926 1.314563e-03    NA        NA
#> SquamousCancer.psi    0.006094858 1.543437e-04    NA        NA
#> hesc_1.alpha         32.787792699 2.402385e-01 0.999  -9122345  GGamma3P
#> hesc_1.scale          1.257693353 1.411931e-04    NA        NA
#> hesc_1.psi            0.028687177 2.105403e-04    NA        NA
#> hesc_2.alpha         33.122072876 2.514906e-01 0.999  -9335335  GGamma3P
#> hesc_2.scale          1.218895512 1.420745e-04    NA        NA
#> hesc_2.psi            0.026573326 2.020459e-04    NA        NA
#> hesc_3.alpha         32.768808631 2.424480e-01 0.999  -9155473  GGamma3P
#> hesc_3.scale          1.221890510 1.390190e-04    NA        NA
#> hesc_3.psi            0.028179917 2.088263e-04    NA        NA
```

D.4 Gibb Entropy

```
sapply(gofs, gibb_entropy)
#>                          1            2           3          4          5
#> Brain          -16.3355642 -16.49118426 -16.6538723 -16.785479 -16.476018
#> Glioma          -1.5442046  -1.52339519  -2.2880404  -2.655461  -1.257910
#> Breast         -14.1290223 -14.64086802 -14.7690211 -15.049374 -14.622104
#> BreastCancer    -1.0363262  -0.09129165   0.5007795   1.944996   2.889619
#> BreastMeta       2.6140133   4.74670048   4.6488231   6.787960   6.069440
#> Colon          -14.5025746 -14.68442360 -14.7705834 -14.476865 -14.458761
#> ColonCancer     -9.9343788 -10.45620418 -10.8676130  -9.574800  -9.469298
#> Colon.M         -6.6516737  -6.16434396  -6.3959542  -5.529075  -4.800270
#> Lung           -16.5117766 -16.78433325 -16.8648907 -17.187394 -16.897568
#> LungCancer      -8.3778963  -9.62496985  -9.9014508  -7.903006  -6.785098
#> Adenocarcinoma   0.1872563   0.56082150   1.4312356   4.472751   3.701478
#> SquamousCancer   2.9199165   5.30011990   4.7709688   6.066446   6.246835
#> hesc_1           1.9888086   1.96176388   1.9458953   1.797189   1.863725
#> hesc_2           1.6571984   1.62271724   1.6099627   1.410320   1.486011
```

```
#> hesc_3           1.7331941   1.70407089   1.6892069   1.526246   1.595393
#>                          6            7           8           9          10
#> Brain           -16.5340823 -16.507387 -16.3832803 -16.1304274 -16.2615985
#> Glioma           -2.1459063   3.596377  -0.8226069  -2.0063424   0.4740653
#> Breast          -14.6230164 -14.558528 -14.3732419 -14.0775605 -14.1950307
#> BreastCancer     -0.7726241   2.387586   1.6330317  -1.2706304   2.3902042
#> BreastMeta        3.5553205   4.728174   6.6801875   3.2372107   5.6472080
#> Colon           -14.8660006 -14.782326 -14.5231867 -14.3794233 -14.7107939
#> ColonCancer     -10.6463744 -10.087572  -9.2724758 -10.3264956 -10.3940179
#> Colon.M          -6.2186059  -5.177822  -4.2311049  -6.4419314  -5.9263336
#> Lung            -16.8167909 -16.734519 -16.8031429 -16.5538102 -16.6229292
#> LungCancer       -6.8374316  -8.026692  -6.2222930  -8.5201006  -6.3909609
#> Adenocarcinoma    0.1201331   1.303370   3.6064467   0.3017814   1.3711074
#> SquamousCancer    5.6714732   5.102156   7.1205625   3.8576902   5.6566730
#> hesc_1            1.9489704   1.981120   1.9041033   1.9883106   1.9583115
#> hesc_2            1.6127520   1.645167   1.5272730   1.6405272   1.5998964
#> hesc_3            1.6921349   1.724381   1.6359978   1.7281326   1.6951670
#>                         11          12          13          14           15
#> Brain           -16.0151180 -16.407945 -16.557895 -16.1188821 -16.39174436
#> Glioma            0.0523540  -1.377244  -1.842551  -1.4460662  -1.91841376
#> Breast          -13.8757123 -14.334960 -15.045866 -14.2376767 -14.49316501
#> BreastCancer      2.1908193   1.254028   3.115025   0.2905565   0.05424745
#> BreastMeta        5.2190604   3.423550   6.286685   4.6710809   3.00268213
#> Colon           -14.1783864 -14.735250 -14.561701 -14.5783329 -15.02817602
#> ColonCancer      -9.7015206 -10.519394  -8.891433 -10.0970865 -10.52376855
#> Colon.M          -5.2097191  -6.517423  -4.503624  -6.1110090  -6.53398368
#> Lung            -16.5501787 -16.650989 -17.002043 -16.6558993 -16.73877998
#> LungCancer       -6.3097483  -6.992510  -8.997379  -8.8025183  -9.00271670
#> Adenocarcinoma    0.8592734   0.582212   4.920410   0.4542331   5.26820108
#> SquamousCancer    5.9033337   3.708725   4.683043   4.3722655   4.17981506
#> hesc_1            1.9145300   1.973459   1.877212   1.9544276   1.98034733
#> hesc_2            1.5353898   1.640089   1.510099   1.6064813   1.64826566
#> hesc_3            1.6457089   1.717524   1.611840   1.6944045   1.72467838
#>                         16          17          18          19           20
#> Brain           -15.8683556 -15.958911 -16.423874 -16.0967461 -15.72324461
#> Glioma           -0.6531279  -2.325970  -1.690764   3.9283356   0.09950097
#> Breast          -13.4187094 -13.606113 -14.583807 -13.1025337 -13.46521050
#> BreastCancer      2.1238321  -4.081463   2.195851  -3.6383356   0.27717364
#> BreastMeta        3.5482391   2.136208   6.895916   2.6100009   4.85955579
#> Colon           -14.5014864 -14.416785 -14.595553 -14.1099367 -13.93562267
#> ColonCancer      -9.6061776 -10.711638  -8.682585  -9.8684426  -8.37946306
#> Colon.M          -5.7350391  -7.776171  -5.409516  -6.1502166  -4.43153684
#> Lung            -16.1034491 -15.969389 -16.895288 -16.1049299 -16.07064476
#> LungCancer       -7.5492531  -7.807797  -9.591348  -3.2789986  -5.39093344
#> Adenocarcinoma    0.9527716  -1.685461   4.412641  -0.7734491   0.62042913
#> SquamousCancer    3.2563133  -0.415287   6.818712   0.7607637   5.79503053
#> hesc_1            2.0983444   2.077587   1.900620   2.0933868   1.97219378
#> hesc_2            1.7647760   1.787076   1.527041   1.7953792   1.59387541
#> hesc_3            1.8428109   1.833182   1.633424   1.8472489   1.70356216
#>                         21          22           X
#> Brain           -15.9895402 -15.641848 -17.6887467
#> Glioma           -0.2292746  -0.970184   0.9399383
#> Breast          -13.4723396 -12.911931 -15.3171304
#> BreastCancer      1.0614490  -1.465153   5.6817322
#> BreastMeta        5.7674271   2.882476   7.4960491
#> Colon           -14.3234547 -13.965082 -15.2493349
#> ColonCancer      -8.7963116 -10.036097  -8.9263229
#> Colon.M          -4.4376154  -7.686259  -2.4120123
#> Lung            -16.2438457 -15.622140 -16.5972587
#> LungCancer       -6.2088584  -9.854450  -3.8995965
#> Adenocarcinoma    1.0814634  -1.242498   4.5424373
```

```
#> SquamousCancer   3.6514052   1.059672   7.8390716
#> hesc_1           1.9929083   2.146656   0.5211911
#> hesc_2           1.6232656   1.839961   0.0479705
#> hesc_3           1.7266344   1.897204   0.2139369
```

The entropy units are: $J \times K^{-1} \times mol^{-1}$

D.5 Helmholtz free energy

```
sapply(gofs, helmholtz_free_energy, kj = TRUE)
#>                          1           2          3          4          5
#> Brain           5.06647523  5.11474080  5.1651985  5.2060164  5.1100368
#> Glioma          0.47893504  0.47248102  0.7096357  0.8235911  0.3901408
#> Breast          4.38211628  4.54086522  4.5806119  4.6675632  4.5350456
#> BreastCancer    0.32141656  0.02831411 -0.1553168 -0.6032407 -0.8962153
#> BreastMeta     -0.81073623 -1.47218915 -1.4418325 -2.1052858 -1.8824369
#> Colon           4.49797351  4.55437398  4.5810965  4.4899996  4.4843846
#> ColonCancer     3.08114757  3.24299173  3.3705902  2.9696242  2.9369028
#> Colon.M         2.06301661  1.91187128  1.9837052  1.7148426  1.4888037
#> Lung            5.12112753  5.20566096  5.2306458  5.3306704  5.2407807
#> LungCancer      2.59840453  2.98518440  3.0709350  2.4511172  2.1043981
#> Adenocarcinoma -0.05807754 -0.17393879 -0.4438977 -1.3872237 -1.1480134
#> SquamousCancer -0.90561211 -1.64383219 -1.4797160 -1.8815081 -1.9374559
#> hesc_1         -0.61682899 -0.60844107 -0.6035194 -0.5573983 -0.5780344
#> hesc_2         -0.51398007 -0.50328575 -0.4993299 -0.4374106 -0.4608864
#> hesc_3         -0.53755015 -0.52851759 -0.5239075 -0.4733652 -0.4948110
#>                          6          7          8           9         10
#> Brain           5.12804562  5.1197662  5.0812744  5.00285207  5.0435348
#> Glioma          0.66555283 -1.1154162  0.2551315  0.62226709 -0.1470314
#> Breast          4.53532854  4.5153273  4.4578610  4.36615538  4.4025888
#> BreastCancer    0.23962937 -0.7405097 -0.5064848  0.39408602 -0.7413218
#> BreastMeta     -1.10268266 -1.4664431 -2.0718601 -1.00402089 -1.7514816
#> Colon           4.61069009  4.5847383  4.5043664  4.45977814  4.5625527
#> ColonCancer     3.30197303  3.1286605  2.8758584  3.20276260  3.2237047
#> Colon.M         1.92870063  1.6059016  1.3122772  1.99796503  1.8380524
#> Lung            5.21572769  5.1902109  5.2114948  5.13416422  5.1556015
#> LungCancer      2.12062941  2.4894785  1.9298442  2.64250921  1.9821565
#> Adenocarcinoma -0.03725927 -0.4042403 -1.1185394 -0.09359752 -0.4252490
#> SquamousCancer -1.75900742 -1.5824335 -2.2084425 -1.19646262 -1.7544171
#> hesc_1         -0.60447316 -0.6144444 -0.5905576 -0.61667455 -0.6073703
#> hesc_2         -0.50019504 -0.5102487 -0.4736837 -0.50880952 -0.4962079
#> hesc_3         -0.52481564 -0.5348168 -0.5074047 -0.53598032 -0.5257561
#>                         11          12         13          14          15
#> Brain           4.96708885  5.0889240  5.1354311  4.99927129  5.08389951
#> Glioma         -0.01623759  0.4271522  0.5714673  0.44849742  0.59499603
#> Breast          4.30355218  4.4459878  4.6664754  4.41581544  4.49505513
#> BreastCancer   -0.67948260 -0.3889367 -0.9661249 -0.09011611 -0.01682485
#> BreastMeta     -1.61869157 -1.0618139 -1.9498154 -1.44873574 -0.93128186
#> Colon           4.39742655  4.5701377  4.5163117  4.52146993  4.66098879
#> ColonCancer     3.00892661  3.2625901  2.7576778  3.13161137  3.26394682
#> Colon.M         1.61579439  2.0213789  1.3967991  1.89532945  2.02651504
#> Lung            5.13303794  5.1643043  5.2731835  5.16582717  5.19153261
#> LungCancer      1.95696845  2.1687268  2.7905371  2.73010104  2.79219258
#> Adenocarcinoma -0.26650366 -0.1805731 -1.5260653 -0.14088039 -1.63393257
#> SquamousCancer -1.83091894 -1.1502610 -1.4524458 -1.35605815 -1.29636964
#> hesc_1         -0.59379149 -0.6120683 -0.5822173 -0.60616573 -0.61420472
#> hesc_2         -0.47620114 -0.5086735 -0.4683573 -0.49825018 -0.51120959
#> hesc_3         -0.51041660 -0.5326900 -0.4999122 -0.52551957 -0.53490900
#>                        16          17         18          19          20
```

```
#> Brain           4.9215705  4.9496563  5.0938644  4.9924058  4.87656432
#> Glioma          0.2025676  0.7213996  0.5243905 -1.2183733 -0.03086023
#> Breast          4.1618127  4.2199361  4.5231677  4.0637508  4.17623504
#> BreastCancer   -0.6587065  1.2658658 -0.6810430  1.1284298 -0.08596540
#> BreastMeta     -1.1004864 -0.6625449 -2.1387682 -0.8094918 -1.50719123
#> Colon           4.4976360  4.4713659  4.5268107  4.3761969  4.32213337
#> ColonCancer     2.9793560  3.3222146  2.6929039  3.0606975  2.59889047
#> Colon.M         1.7787224  2.4117793  1.6777614  1.9074897  1.37444115
#> Lung            4.9944847  4.9529060  5.2400736  4.9949440  4.98431047
#> LungCancer      2.3414009  2.4215882  2.9747567  1.0169814  1.67199801
#> Adenocarcinoma -0.2955021  0.5227458 -1.3685805  0.2398852 -0.19242610
#> SquamousCancer -1.0099456  0.1288013 -2.1148235 -0.2359509 -1.79732872
#> hesc_1         -0.6508015 -0.6443637 -0.5894774 -0.6492639 -0.61167590
#> hesc_2         -0.5473453 -0.5542617 -0.4736116 -0.5568369 -0.49434046
#> hesc_3         -0.5715478 -0.5685615 -0.5066063 -0.5729242 -0.52835980
#>                         21         22           X
#> Brain           4.95915590  4.8513192  5.48616480
#> Glioma          0.07110951  0.3009026 -0.29152187
#> Breast          4.17844613  4.0046353  4.75060800
#> BreastCancer   -0.32920840  0.4544172 -1.76218925
#> BreastMeta     -1.78876751 -0.8940000 -2.32489961
#> Colon           4.44241946  4.3312703  4.72958123
#> ColonCancer     2.72817605  3.1126954  2.76849906
#> Colon.M         1.37632643  2.3838932  0.74808562
#> Lung            5.03802873  4.8452066  5.14763980
#> LungCancer      1.92567743  3.0563578  1.20945986
#> Adenocarcinoma -0.33541589  0.3853606 -1.40883692
#> SquamousCancer -1.13248331 -0.3286571 -2.43128804
#> hesc_1         -0.61810050 -0.6657855 -0.16164741
#> hesc_2         -0.50345582 -0.5706638 -0.01487805
#> hesc_3         -0.53551566 -0.5884179 -0.06635253
```

The energy units are: $kJ \times mol^{-1}$

E. R Script for the Analysis of Arabidopsis data set

The R script example given here is limited to the 3rd generation, but it can be extended to all generations.

```
library(MethylIT)
library(MethylIT)
library(ggplot2)
library(ggpmisc)
library(dplyr)
```

For the sake of brevity the analysis is applied here only to the wildtype 3rd generation control and to memory line 3rd generation. The same R script was applied to all set of samples.

E.1 Download files from GEO

If read count datasets are available at GEO database, then MethylIT function getGEOSuppFiles can be used to download read count datasets from GEO.

Users can always download manually by themselves and then read them into R with function readCounts2GRangesList

Wildtype control samples:

```
wt3_files = getGEOSuppFiles(GEO = c("GSM3704281", "GSM3704282", "GSM3704283",
                                    "GSM3704284", "GSM3704285"),
                            verbose = FALSE)
```

Memory line 3rd generation:

```
mm3_files = getGEOSuppFiles(GEO = c("GSM3704257","GSM3704258", "GSM3704259",
                                    "GSM3704260","GSM3704261"),
                            verbose = FALSE)
```

E.2 Reading datasets

Reading wildtype control samples

```
sn = c("wt3_1", "wt3_2", "wt3_3", "wt3_4", "wt3_5")

wt3 = readCounts2GRangesList(filenames = wt3_files, sample.id = sn,
                             columns = c(seqnames = 1, start = 2,
                                         mC = 4, uC = 5 ),
                             verbose = FALSE)
```

Reading memory-line 3rd generation dataset

```
sn = c("m3_1", "m3_2", "m3_3", "m3_4", "m3_5")

mm3 = readCounts2GRangesList(filenames = mm3_files, sample.id = sn,
                             columns = c(seqnames = 1, start = 2,
                                         mC = 4, uC = 5 ),
                             verbose = FALSE)
```

E.3 The reference sample

```
ref <- poolFromGRlist(LR = wt3, stat = "sum",
                      columns = 1:2,
                      num.cores = 5L,
                      verbose = TRUE)
```

E.4 J-Divergence

```
ptm <- proc.time()
idiv <- estimateDivergence(ref = ref,
                           indiv = wt3,
                           Bayesian = TRUE,
                           JD = TRUE,
                           min.coverage = 5,
                           high.coverage = 500,
                           percentile = 0.999,
                           num.cores = 3L,
                           verbose = FALSE)

cat((proc.time() - ptm)[3]/60, "minutes.", date()) # in min
```

Results of this computation are available on PSU GitLab at https://git.psu.edu/genomath/datasets/-/tree/main/at_mutants.

E.5 Estimation of the best fitted probability distribution model

Depending on your computation capability, this step takes considerable computational time.

The RData files with information divergence values can be downloaded from GitLab using the following script:

```
## ===== Download methylation data from GitLab ========

url <- paste0("https://git.psu.edu/genomath/datasets/-/raw/main/at_mutants/",
              "/idiv/idiv_memory-col0-control-gen3_all-contexts_sample-",
              1:5,".RData")
temp <- tempfile(fileext = ".RData")

idiv <- vector(mode = "list", length = 5)
names(idiv) <- c("wt3_1", "wt3_2", "wt3_3", "wt3_4", "wt3_5")
for (k in 1:5) {
  download.file(url = url[k], destfile = temp)
  load(temp)
  file.remove(temp)
  idiv[[k]] <- jdiv
}
rm(temp, url)
```

The best fitted probability distribution model can be found applying function gofReport:

```
jdiv <- lapply(idiv, function(x) {
  x <- split(x, as.factor(seqnames(x)))
  x<- structure(as.list(x), class = "InfDiv")
  return(x)
})
jdiv

d <- c("Weibull2P", "Weibull3P", "Gamma2P", "Gamma3P","GGamma3P","GGamma4P")
gof_jd <- lapply(
  jdiv,
  gofReport,
  model = d,
  column = 10L,
  num.cores = 6L,
  alt_models = TRUE,
  r.cv = TRUE,
  output = "all",
  verbose = FALSE)
```

The same can be done for the memory line dataset.

E.6 Thermodynamic state variables

Results can be downloaded from PSU GitLab at https://git.psu.edu/genomath/datasets/-/tree/main/at_mutants.

The Gibb entropy can be estimated by applying function gibb_entropy

Wildtype entropy and Helmholtz free energy

```
url <- paste0("https://git.psu.edu/genomath/datasets/-/raw/main/at_mutants/",
              "/gofs/gof-jd-by-chr_memory-col0-control-gen3_all-contexts.RData")
temp <- tempfile(fileext = ".RData")
download.file(url = url, destfile = temp)
load(temp)
file.remove(temp)
#> [1] TRUE

## Entropy
t(sapply(gof_jd, gibb_entropy))
#>                1         2         3         4         5
#> wt3_1 -12.09485 -13.09161 -12.85370 -12.87477 -12.39782
#> wt3_2 -12.23864 -13.20160 -12.82698 -12.95472 -12.44664
#> wt3_3 -12.58199 -13.61125 -13.31159 -13.40339 -12.87161
#> wt3_4 -12.19005 -13.28911 -12.88366 -13.00763 -12.53417
#> wt3_5 -13.00950 -14.07444 -13.80581 -13.83110 -13.33332
```

The Gibb entropy can be estimated applying function helmholtz_free_energy

```
t(sapply(gof_jd, helmholtz_free_energy))
#>              1        2        3        4        5
#> wt3_1 3751.217 4060.363 3986.574 3993.111 3845.185
#> wt3_2 3795.814 4094.477 3978.287 4017.906 3860.326
#> wt3_3 3902.303 4221.530 4128.590 4157.063 3992.129
#> wt3_4 3780.745 4121.616 3995.866 4034.315 3887.474
#> wt3_5 4034.897 4365.187 4281.872 4289.717 4135.330
```

Memory line entropy and Helmholtz free energy

```
url <- paste0("https://git.psu.edu/genomath/datasets/-/raw/main/at_mutants/",
              "/gofs/gof-jd-by-chr_memory-gen3_all-contexts.RData")
temp <- tempfile(fileext = ".RData")
download.file(url = url, destfile = temp)
load(temp)
file.remove(temp)
#> [1] TRUE

## Entropy
t(sapply(gof_jd, gibb_entropy))
#>               1         2         3         4          5
#> m3_1  -9.504246 -10.59310 -10.36580 -10.37016  -9.850248
#> m3_2  -9.617158 -10.69061 -10.53673 -10.52800 -10.013634
#> m3_3  -9.391835 -10.47471 -10.26938 -10.26390  -9.839075
#> m3_4 -10.335803 -11.40691 -11.29229 -11.31029 -10.824549
#> m3_5  -9.687667 -10.73626 -10.53089 -10.52618 -10.083295
```

The Gibb entropy:

```
t(sapply(gof_jd, helmholtz_free_energy))
#>             1        2        3        4        5
#> m3_1 2947.742 3285.450 3214.954 3216.304 3055.054
#> m3_2 2982.762 3315.694 3267.967 3265.259 3105.729
#> m3_3 2912.878 3248.730 3185.049 3183.348 3051.589
#> m3_4 3205.649 3537.854 3502.303 3507.888 3357.234
#> m3_5 3004.630 3329.852 3266.155 3264.696 3127.334
```

F. R Script for the Analysis of Entropy fluctuations

The libraries required and auxiliary functions for the analysis are loaded as

```
library(MethylIT)
library(ggplot2)
library(ggpmisc)
library(dplyr)

## ----- Auxiliary functions ------
lm_eqn <- function(x, y){
    m <- lm(y ~ x + 0)
    eq <- substitute(italic(y) == b %.% italic(x)*","~~italic(R[abj])^2~"="~r2,
         list(
              b = format(unname(coef(m)[1]), digits = 3),
              r2 = format(summary(m)$adj.r.squared, digits = 3)))
    as.character(as.expression(eq));
}

lm_eqn2 <- function(x, y){
    m <- lm(y ~ x)
    b = coef(m)[1]
    a = coef(m)[2]

    if (b > 0)
        eq <- substitute(italic(y) == a %.% italic(x) + b*","~~italic(R[abj])^2~"="~r2,
            list(a = format(unname(a), digits = 3),
                b = format(unname(b), digits = 3),
                r2 = format(summary(m)$adj.r.squared, digits = 3)))
    else {
        b <- abs(b)
        eq <- substitute(italic(y) == a %.% italic(x) - b*","~~italic(R[abj])^2~"="~r2,
            list(a = format(unname(a), digits = 3),
                b = format(unname(b), digits = 3),
                r2 = format(summary(m)$adj.r.squared, digits = 3)))
    }
    as.character(as.expression(eq));
}
```

F.1 Arabidopsis dataset

The *Arabidopsis thaliana* methylome datasets of *msh1* memory and non-memory (normal phenotype) sibling plants were derived from the *msh1* mutant. As described in reference (35), an MSH1-RNAi transgene positive plant was self-pollinated and the transgene was segregated in the subsequent generation. Of transgene null plants, 20% plants displayed delay in flowering, smaller size, and pale green leaves, termed a ‘memory’ phenotype. Memory plants were self-pollinated for six generations and plant DNA samples from each generation were bisulfite sequenced.

The dataset generation 2-6 can be accessed with GEOaccession number GSE129303 and GSE118874.The RData files with J-information-divergence and with the best fitted models and

corresponding goodness-of-fit used in all the computation reported in the main text are available at PSU GitLab: https://git.psu.edu/genomath/datasets.

```
## ===================== Memory GOFs ======================
boltz_fact <- function(s) {
    nms <- names(s)
    r <- lapply(seq(s), function(k) {
        r <- data.frame(boltzman_factor(s[[k]], only.sum = FALSE))
        r$abs_ent <- abs(r$ent)
        r$sample <- rep(nms[k], nrow(r))
        r$chr <- rownames(r)
        rownames(r) <- NULL
        return(r)
    })
    do.call(rbind,r)
}


## ===== Download methylation data from GitLab ========
boltz_fact <- function(s) {
    nms <- names(s)
    r <- lapply(seq(s), function(k) {
        r <- data.frame(boltzman_factor(s[[k]], only.sum = FALSE))
        r$abs_ent <- abs(r$ent)
        r$sample <- rep(nms[k], nrow(r))
        r$chr <- paste0("chr", rownames(r))
        rownames(r) <- NULL
        return(r)
    })
    do.call(rbind,r)
}


## ===== Download methylation data from GitLab ========
samples <- c("memory-col0-control-gen3",
             "non-memory-gen1",
             "memory-gen1",
             "memory-gen2",
             "memory-gen3",
             "memory-gen4",
             "memory-gen5",
             "memory-gen6",
             "col0-control-met1",
             "met1")

url <- paste0("https://git.psu.edu/genomath/datasets/-/raw/main/at_mutants/",
              "gofs/gof-jd-by-chr_", samples,"_all-contexts.RData")

nms <- c("ctrl", "nm", "mm1", "mm3", "mm4", "mm5", "mm6", "ctrl_met1", "met1")
bf <- c()
for (k in seq(samples)) {
    temp <- tempfile(fileext = ".RData")
    download.file(url = url[k], destfile = temp)
    load(temp)
    file.remove(temp)
    bf <- rbind(bf, boltz_fact(gof_jd))
}
rm(temp, url)
```

```
## To distinguish met1 wildtype control from memmory control
bf$sample[201:220] <- gsub("wt", "ctrl_met1",bf$sample[201:220])

df <- bf %>% reframe(means = mean(exp_sum),
                     n = n(), sd = sd(exp_sum),
                     se = sd / sqrt(n),
                     sample = sample, .by = chr)
df$chr <- factor(df$chr,
                  levels = paste0("chr", 1:5))
df$species <- "Arabidopsis"

dt.0 <- bf %>% group_by(chr,sample) %>% summarise(means = mean(exp_sum))
dt.0$species <- "Arabidopsis"
```

F.2 Plotting linear regression for Arabidopsis dataset

```
bf. <- bf[ !grepl("met1", bf$sample), ]
bf.0 <- bf[ grepl("met1", bf$sample), ]
bf.1 <- bf[ grepl("ctrl_met1", bf$sample), ]
bf.2 <- bf.0[ !grepl("ctrl_", bf.0$sample), ] ## Only met1 mutant

lm_bf <- lm(abs_ent ~ nu, data = bf.)
summary(lm_bf)
#>
#> Call:
#> lm(formula = abs_ent ~ nu, data = bf.)
#>
#> Residuals:
#>       Min        1Q    Median        3Q       Max
#> -0.056783 -0.020092 -0.004458  0.018324  0.085323
#>
#> Coefficients:
#>             Estimate Std. Error t value Pr(>|t|)
#> (Intercept)  2.34582    0.01019  230.28   <2e-16 ***
#> nu          -7.65431    0.08357  -91.59   <2e-16 ***
#> ---
#> Signif. codes:  0 '***' 0.001 '**' 0.01 '*' 0.05 '.' 0.1 ' ' 1
#>
#> Residual standard error: 0.02813 on 198 degrees of freedom
#> Multiple R-squared:  0.9769, Adjusted R-squared:  0.9768
#> F-statistic:  8389 on 1 and 198 DF,  p-value: < 2.2e-16
```

A regression analysis only met1 mutant

```
lm_met1 <- lm(abs_ent ~ nu, data = bf.2)
summary(lm_met1)
#>
#> Call:
#> lm(formula = abs_ent ~ nu, data = bf.2)
#>
#> Residuals:
#>       Min        1Q    Median        3Q       Max
#> -0.012960 -0.008742 -0.005859  0.006890  0.025279
#>
#> Coefficients:
#>             Estimate Std. Error t value Pr(>|t|)
#> (Intercept) -0.59953    0.05404  -11.09 5.33e-08 ***
#> nu           1.26404    0.08463   14.94 1.45e-09 ***
#> ---
#> Signif. codes:  0 '***' 0.001 '**' 0.01 '*' 0.05 '.' 0.1 ' ' 1
```

```
#>
#> Residual standard error: 0.01336 on 13 degrees of freedom
#> Multiple R-squared:  0.9449, Adjusted R-squared:  0.9407
#> F-statistic: 223.1 on 1 and 13 DF,  p-value: 1.455e-09
```

F.2.1 The graphic $\frac{|S|}{k_B}$ vs ν

```
p <- ggplot(bf., aes(x = nu, y = abs_ent)) +
  geom_point(data = bf.) +
  geom_smooth(data = bf., formula = y ~ x, method=lm , se = TRUE) +
  theme_light(base_family = "serif") +
  theme(text = element_text(size = 20, family = "serif")) +
  geom_text(x = 0.11, y = 1.18, label = lm_eqn2(x = bf.$nu, y = bf.$abs_ent),
            parse = TRUE, family = "serif",
            size = 4)

p0 <- ggplot(bf.0, aes(x = nu, y = abs_ent)) +
  geom_smooth(data = bf.2, formula = y ~ x,
            method=lm , se = TRUE, fullrange = F) +
  geom_point(color="red") +
  geom_smooth(data = bf.1, formula = y ~ x,
             method=lm , se = TRUE, fullrange = F) +
  geom_point(data = bf.1) +
  theme_light(base_family = "serif") +
  theme(axis.title.x = element_blank(),
        axis.title.y = element_blank(),
        text = element_text(size = 10, family = "serif")) +
  geom_text(x = 0.45, y = 0.59, label = lm_eqn2(x=bf.1$nu, y=bf.1$abs_ent),
            parse = TRUE, family = "serif",
            size = 3) +
  geom_text(x = 0.45, y = 0.3, label = lm_eqn2(x=bf.2$nu, y=bf.2$abs_ent),
            parse = TRUE, family = "serif",
            size = 3, color = "red")
p + annotation_custom(grob = ggplotGrob(p0),
                      xmin = 0.125, xmax = 0.175,
                      ymin = 1.41, ymax = 1.82 )
```

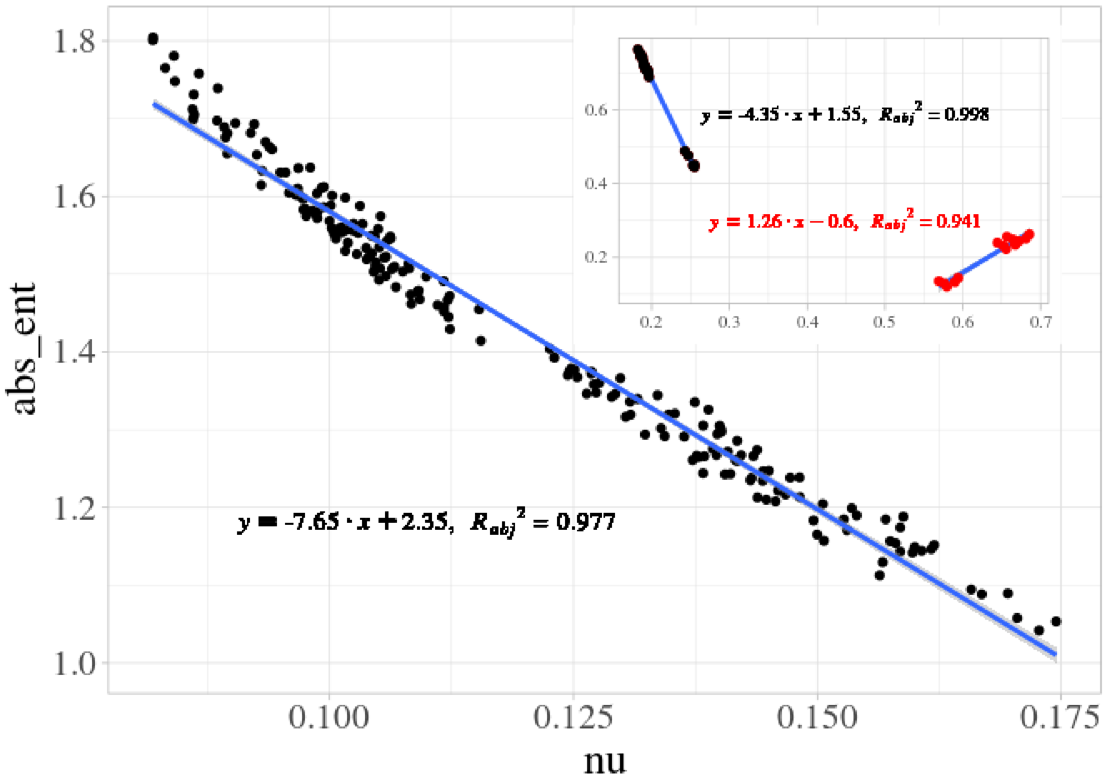

F2.2 The graphic $e^{-\frac{|S|}{k_B}}$ vs ν

```
p <- ggplot(bf., aes(x = nu, y = exp_ent)) +
  geom_point(data = bf.) +
  geom_smooth(data = bf., formula = y ~ x + 0, method=lm , se = TRUE) +
  theme_light(base_family = "serif") +
  theme(text = element_text(size = 20, family = "serif")) +
  geom_text(x = 0.14, y = 0.22, label = lm_eqn(x=bf.$nu, y=bf.$exp_ent),
            parse = TRUE, family = "serif",
            size = 4)

p0 <- ggplot(bf.0, aes(x = nu, y = exp_ent)) +
  geom_point(color="red") +
  geom_smooth(data = bf.1, formula = y ~ x,
              method=lm , se = TRUE, fullrange = F) +
  geom_smooth(data = bf.2, formula = y ~ x,
              method=lm , se = TRUE, fullrange = F) +
  geom_point(data = bf.1) +
  theme_light(base_family = "serif") +
  theme(axis.title.x = element_blank(),
        axis.title.y = element_blank(),
        text = element_text(size = 10, family = "serif")) +
  geom_text(x = 0.48, y = 0.52, label = lm_eqn(x=bf.1$nu, y=bf.1$exp_ent),
            parse = TRUE, family = "serif",
            size = 3, color = "blue") +
  geom_text(x = 0.48, y = 0.7, label = lm_eqn2(x=bf.2$nu, y=bf.2$exp_ent),
            parse = TRUE, family = "serif",
            size = 3, color = "red")

p + annotation_custom(grob = ggplotGrob(p0),
```

```
                         xmin = 0.078, xmax = 0.125,
                         ymin = 0.26, ymax = 0.36 )
```

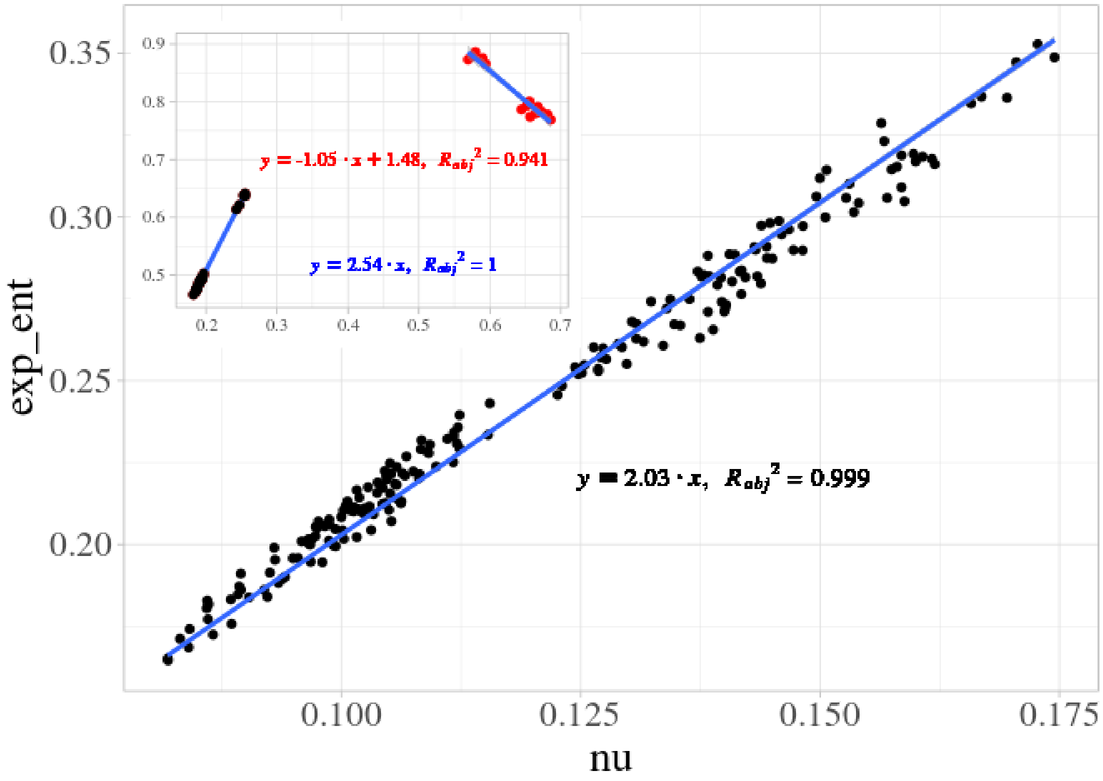

Regression analysis for *met1* mutant

```
lm_met1 <- lm(exp_ent ~ nu, data = bf.2)
summary(lm_met1)
#> 
#> Call:
#> lm(formula = exp_ent ~ nu, data = bf.2)
#> 
#> Residuals:
#>       Min        1Q    Median        3Q       Max 
#> -0.020439 -0.005888  0.004886  0.006880  0.010852 
#> 
#> Coefficients:
#>             Estimate Std. Error t value Pr(>|t|)    
#> (Intercept)  1.48208    0.04467   33.17 5.97e-14 ***
#> nu          -1.04680    0.06996  -14.96 1.42e-09 ***
#> ---
#> Signif. codes:  0 '***' 0.001 '**' 0.01 '*' 0.05 '.' 0.1 ' ' 1
#> 
#> Residual standard error: 0.01104 on 13 degrees of freedom
#> Multiple R-squared:  0.9451, Adjusted R-squared:  0.9409 
#> F-statistic: 223.9 on 1 and 13 DF,  p-value: 1.423e-09
```

F2.3 The graphic $e^{-\frac{|S|}{k_B}}$ vs $e^{-\nu}$

```
p <- ggplot(bf.0, aes(x = exp_nu, y = exp_ent)) +
  geom_point(data = bf.) +
  geom_smooth(data = bf., formula = y ~ x, method=lm , se = TRUE) +
  theme_light(base_family = "serif") +
  theme(text = element_text(size = 20, family = "serif")) +
  geom_text(x = 0.87, y = 0.18,
            label = lm_eqn(x=bf.$exp_nu, y=bf.$exp_ent),
            parse = TRUE, family = "serif",
            size = 4)

p0 <- ggplot(bf.0, aes(x = exp_nu, y = exp_ent)) +
  geom_point(color="red") +
  geom_smooth(data = bf.1, formula = y ~ x,
              method=lm , se = TRUE, fullrange = F) +
  geom_smooth(data = bf.2, formula = y ~ x,
              method=lm , se = TRUE, fullrange = F) +
  geom_point(data = bf.1) +
  theme_light(base_family = "serif") +
  theme(axis.title.x = element_blank(),
        axis.title.y = element_blank(),
        text = element_text(size = 10, family = "serif")) +
  geom_text(x = 0.65, y = 0.52, label = lm_eqn2(x=bf.1$exp_nu, y=bf.1$exp_ent),
            parse = TRUE, family = "serif",
            size = 3) +
  geom_text(x = 0.65, y = 0.8, label = lm_eqn2(x=bf.2$exp_nu, y=bf.2$exp_ent),
            parse = TRUE, family = "serif",
            size = 3, color = "red")

p + annotation_custom(grob = ggplotGrob(p0),
                      xmin = 0.88, xmax = 0.924,
```

```
                          ymin = 0.26, ymax = 0.36 )
```

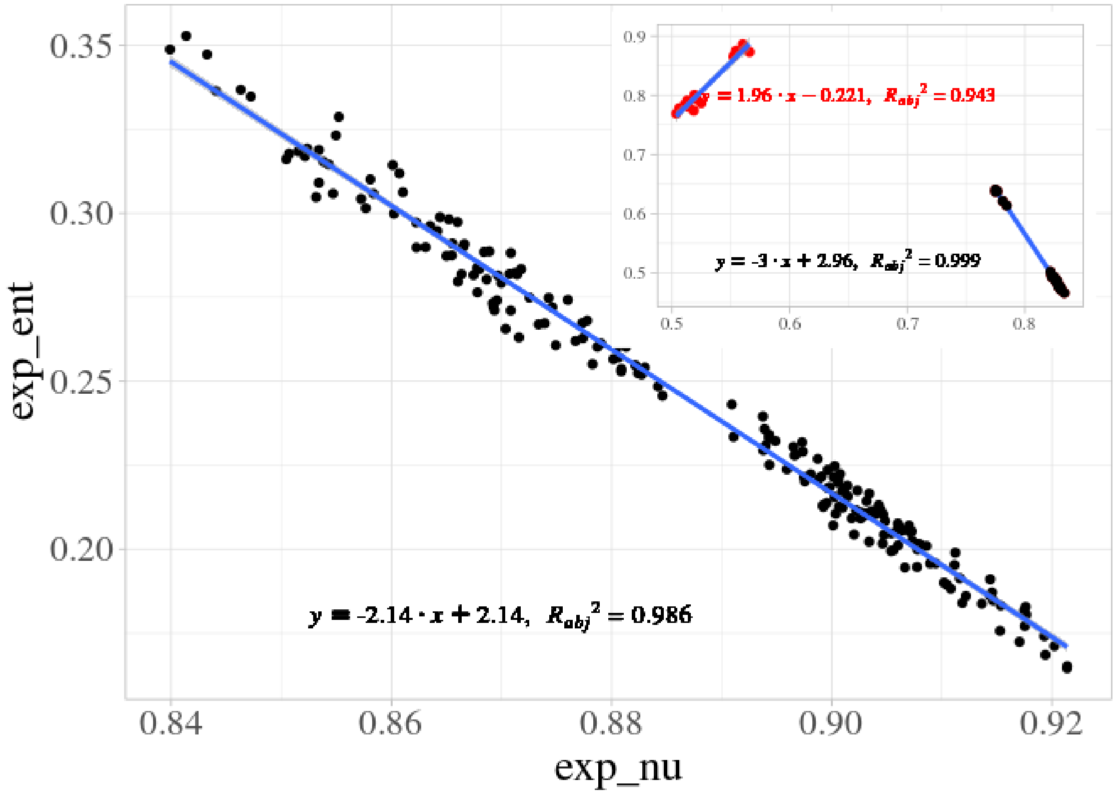

F.3 Cancer dataset

F.3.1 All tissues

```
## ===== Download methylation data from GitLab ========
url <- paste0("https://git.psu.edu/genomath/datasets/-/raw/",
              "main/cancer_data/gofs/jdiv_gof_per_chr_1-X__cancer_03-21-2023.RData")
temp <- tempfile(fileext = ".RData")
download.file(url = url, destfile = temp)
load(temp)
file.remove(temp); rm(temp, url)
#> [1] TRUE

## Auxiliary function to select states
bfs <- function(s, status = "all") {
     nms <- names(s)
     r <- lapply(seq(s), function(k) {
          r <- data.frame(boltzman_factor(s[[k]], only.sum = FALSE))
          r$cancer <- c(0,0,0,0,0,0,1,1,1,1,1,0,1,1,1)
          r$abs_ent <- abs(r$ent)
          r$chr <- rep(nms[k], nrow(r))
          r$sample <- rownames(r)
          if (status != "all")
               r <- r[ r$cancer == status,] ## remove cancer samples,
          rownames(r)
          return(r)
```

```
    })
    do.call(rbind,r)
}
```

Summarize by chromosome

```
bf1 <- bfs(gofs, status = "all")

dt <- bf1 %>% reframe(means = mean(exp_sum),
                    n = n(), sd = sd(exp_sum),
                    se = sd / sqrt(n), .by = chr)
dt$chr <- factor(dt$chr,
                 levels = c(paste(1:22), "X"))
dt
```

Summarize by sample by chromosome

```
dt. <- bf1 %>% reframe(
    means = mean(exp_sum),
    .by = c(sample, chr))
dt.$sample <- factor(dt$sample,
                    levels = c(
                            "Brain", "Glioma",
                            "Breast", "BreastCancer", "BreastMeta",
                            "Colon", "ColonCancer", "Colon.M",
                            "Lung", "LungCancer",
                            "Adenocarcinoma", "SquamousCancer",
                            "hesc_1", "hesc_2", "hesc_3"
                            ), ordered = TRUE)

dt.
```

F.3.2 Healthy tissue

Summary by chromosome

```
bf2 <- bfs(gofs, status = 0)
bf2 <- bfs(gofs, status = 0)
dt2 <- bf2 %>% group_by(chr) %>% summarise(means = mean(exp_sum),
                                           n = n(), sd = sd(exp_sum),
                                           se = sd / sqrt(n))
dt2$chr <- factor(dt2$chr,
                 levels = c(paste(1:22), "X"))
dt2
```

F.3.3 Cancer tissue

Summary by chromosome

```
bf3 <- bfs(gofs, status = 1)

dt3 <- bf3 %>% group_by(chr) %>% summarise(means = mean(exp_sum),
                                            n = n(), sd = sd(exp_sum),
                                            se = sd / sqrt(n))
dt3$chr <- factor(dt3$chr,
                 levels = c(paste(1:22), "X"))
```

```
dt3
#> # A tibble: 23 × 5
#>    chr   means     n    sd      se
#>    <fct> <dbl> <int> <dbl>   <dbl>
#> 1     1 1.198878 8 0.1312248 0.04639498
#> 2    10 1.109021 8 0.2248144 0.07948389
#> 3    11 1.137610 8 0.2408828 0.08516494
#> 4    12 1.165628 8 0.1405562 0.04969412
#> 5    13 1.052438 8 0.1937275 0.06849300
#> 6    14 1.165422 8 0.2006640 0.07094545
#> 7    15 1.127637 8 0.1606376 0.05679397
#> 8    16 1.154843 8 0.1589614 0.05620134
#> 9    17 1.197140 8 0.1009219 0.03568129
#> 10   18 1.059132 8 0.2457526 0.08688666
#> 11   19 1.200559 8 0.1038117 0.03670298
#> 12    2 1.150663 8 0.2077028 0.07343403
#> 13   20 1.178116 8 0.2388392 0.08444242
#> 14   21 1.178698 8 0.2138940 0.07562296
#> 15   22 1.208435 8 0.1197313 0.04233140
#> 16    3 1.135175 8 0.1745833 0.06172453
#> 17    4 1.059208 8 0.2111453 0.07465115
#> 18    5 1.059918 8 0.2120054 0.07495523
#> 19    6 1.170266 8 0.1969962 0.06964868
#> 20    7 1.082611 8 0.1537687 0.05436545
#> 21    8 1.089663 8 0.2554717 0.09032289
#> 22    9 1.187836 8 0.1614177 0.05706976
#> 23    X 1.099844 8 0.3172274 0.11215682
```

Removing stem cells from the subgroup of healthy tissue

```
bf4 <- bf2[ !grepl("hesc", bf2$sample), ] ## Without stem cells

lm_bf4 <- lm(abs_ent ~ nu, data = bf4)
summary(lm_bf4)
#>
#> Call:
#> lm(formula = abs_ent ~ nu, data = bf4)
#>
#> Residuals:
#>       Min        1Q    Median        3Q       Max
#> -0.052490 -0.015786 -0.001593  0.012451  0.061780
#>
#> Coefficients:
#>             Estimate Std. Error t value Pr(>|t|)
#> (Intercept)   2.4869     0.0116  214.43   <2e-16 ***
#> nu           -7.7395     0.1386  -55.85   <2e-16 ***
#> ---
#> Signif. codes:  0 '***' 0.001 '**' 0.01 '*' 0.05 '.' 0.1 ' ' 1
#>
#> Residual standard error: 0.02312 on 90 degrees of freedom
#> Multiple R-squared:  0.972,  Adjusted R-squared:  0.9716
#> F-statistic:  3119 on 1 and 90 DF,  p-value: < 2.2e-16
```

Only stem cells

```
bf5 <- bf2[ grepl("hesc", bf2$sample), ] ## Only stem cells
bf5
#>           exp_ent    exp_nu        nu         ent         Z     sigma   exp_sum
#> hesc_1  0.7872584 0.5486820 0.6002362 0.239198693 0.7598479 0.4912344 1.335940
```

```
#> hesc_2   0.8192917 0.5701410 0.5618716 0.199315150 0.7328403 0.4408452 1.389433
#> hesc_3   0.8118373 0.5612454 0.5775970 0.208455338 0.7676309 0.4578465 1.373083
#> hesc_11  0.7898234 0.5513420 0.5954000 0.235945962 0.7541076 0.4851237 1.341165
#> hesc_21  0.8226964 0.5729535 0.5569508 0.195168024 0.7255086 0.4351289 1.395650
#> hesc_31  0.8146859 0.5639276 0.5728295 0.204952619 0.7612925 0.4520799 1.378613
#> hesc_12  0.7913322 0.5506060 0.5967358 0.234037405 0.7592457 0.4861968 1.341938
#> hesc_22  0.8239594 0.5723185 0.5580596 0.193634002 0.7289467 0.4360628 1.396278
#> hesc_32  0.8161437 0.5632287 0.5740694 0.203164889 0.7660845 0.4530571 1.379372
#> hesc_13  0.8056127 0.5662815 0.5686640 0.216152187 0.7062931 0.4542194 1.371894
#> hesc_23  0.8439834 0.5880711 0.5309075 0.169622460 0.6787042 0.4066140 1.432054
#> hesc_33  0.8322976 0.5788015 0.5467957 0.183565201 0.7126246 0.4230465 1.411099
#> hesc_14  0.7991916 0.5627105 0.5749900 0.224154615 0.7065281 0.4630888 1.361902
#> hesc_24  0.8363350 0.5842536 0.5374202 0.178726075 0.6812828 0.4149824 1.420589
#> hesc_34  0.8254046 0.5751934 0.5530489 0.191881615 0.7135424 0.4314339 1.400598
#> hesc_15  0.7910396 0.5506747 0.5966110 0.234407256 0.7552130 0.4865615 1.341714
#> hesc_25  0.8236830 0.5722119 0.5582459 0.193969483 0.7271924 0.4365492 1.395895
#> hesc_35  0.8158563 0.5632476 0.5740359 0.203517051 0.7626649 0.4534429 1.379104
#> hesc_16  0.7879868 0.5498443 0.5981201 0.238273965 0.7567202 0.4886624 1.337831
#> hesc_26  0.8204780 0.5714696 0.5595440 0.197868162 0.7279150 0.4383475 1.391948
#> hesc_36  0.8126982 0.5624440 0.5754638 0.207395395 0.7638705 0.4553988 1.375142
#> hesc_17  0.7953198 0.5614329 0.5772629 0.229010988 0.7068271 0.4663717 1.356753
#> hesc_27  0.8321948 0.5830700 0.5394480 0.183688725 0.6807082 0.4178971 1.415265
#> hesc_37  0.8213834 0.5739510 0.5552113 0.196765312 0.7134801 0.4345027 1.395334
#> hesc_18  0.7873056 0.5530941 0.5922271 0.239138804 0.7371583 0.4831627 1.340400
#> hesc_28  0.8209360 0.5745895 0.5540995 0.197310074 0.7113568 0.4333647 1.395526
#> hesc_38  0.8123317 0.5656260 0.5698222 0.207846577 0.7446657 0.4502883 1.377958
#> hesc_19  0.7901514 0.5562096 0.5866101 0.235530741 0.7252497 0.4768761 1.346361
#> hesc_29  0.8249576 0.5777351 0.5486399 0.192423306 0.6997218 0.4275752 1.402693
#> hesc_39  0.8155588 0.5687363 0.5643384 0.203881732 0.7324922 0.4443704 1.384295
#> hesc_110 0.7943231 0.5618931 0.5764437 0.230265034 0.6961856 0.4668430 1.356216
#> hesc_210 0.8313828 0.5833415 0.5389824 0.184664946 0.6732827 0.4183920 1.414724
#> hesc_310 0.8204246 0.5743546 0.5545083 0.197933281 0.7035265 0.4349720 1.394779
#> hesc_111 0.7887132 0.5490466 0.5995719 0.237352561 0.7537142 0.4910064 1.337760
#> hesc_211 0.8209794 0.5704782 0.5612802 0.197257308 0.7265181 0.4407840 1.391458
#> hesc_311 0.8133688 0.5616106 0.5769466 0.206570649 0.7613680 0.4576416 1.374979
#> hesc_112 0.7978962 0.5598856 0.5800228 0.225776715 0.7248559 0.4674523 1.357782
#> hesc_212 0.8339155 0.5816479 0.5418899 0.181623191 0.6963025 0.4187982 1.415563
#> hesc_312 0.8237734 0.5724439 0.5578405 0.193859792 0.7313551 0.4354799 1.396217
#> hesc_113 0.7905206 0.5532696 0.5919099 0.235063615 0.7352220 0.4827589 1.343790
#> hesc_213 0.8243045 0.5746941 0.5539173 0.193215293 0.7095709 0.4331609 1.398999
#> hesc_313 0.8156336 0.5657964 0.5695210 0.203790025 0.7428104 0.4499109 1.381430
#> hesc_114 0.7880600 0.5487275 0.6001533 0.238181037 0.7579694 0.4913026 1.336788
#> hesc_214 0.8201723 0.5701894 0.5617867 0.198240793 0.7310068 0.4409142 1.390362
#> hesc_314 0.8126692 0.5612949 0.5775088 0.207431131 0.7656776 0.4579098 1.373964
#> hesc_115 0.7769550 0.5456906 0.6057031 0.252372820 0.7411421 0.5020337 1.322646
#> hesc_215 0.8087594 0.5669861 0.5674205 0.212253762 0.7169016 0.4509181 1.375746
#> hesc_315 0.8012044 0.5582237 0.5829956 0.221639207 0.7491558 0.4680742 1.359428
#> hesc_116 0.7788971 0.5351637 0.6251826 0.249876316 0.8128248 0.5203154 1.314061
#> hesc_216 0.8065932 0.5563790 0.5863056 0.214935851 0.7861472 0.4676944 1.362972
#> hesc_316 0.8021328 0.5477474 0.6019410 0.220481138 0.8220587 0.4851865 1.349880
#> hesc_117 0.7956530 0.5606245 0.5787039 0.228592076 0.7142401 0.4672667 1.356278
#> hesc_217 0.8322181 0.5823623 0.5406626 0.183660769 0.6867469 0.4186190 1.414580
#> hesc_317 0.8216377 0.5731754 0.5565635 0.196455708 0.7206481 0.4353152 1.394813
#> hesc_118 0.7774184 0.5360088 0.6236047 0.251776558 0.7939261 0.5204377 1.313427
#> hesc_218 0.8057881 0.5569957 0.5851977 0.215934487 0.7703822 0.4681137 1.362784
#> hesc_318 0.8007768 0.5485310 0.6005114 0.222172975 0.8037128 0.4853677 1.349308
#> hesc_119 0.7888332 0.5605233 0.5788845 0.237200390 0.6859367 0.4720294 1.349356
#> hesc_219 0.8255552 0.5818174 0.5415987 0.191699149 0.6657045 0.4231998 1.407373
#> hesc_319 0.8147358 0.5729424 0.5569701 0.204891433 0.6937243 0.4398961 1.387678
#> hesc_120 0.7868704 0.5585177 0.5824689 0.239691773 0.7050064 0.4742911 1.345388
#> hesc_220 0.8226422 0.5799764 0.5447678 0.195233975 0.6812109 0.4252713 1.402619
```

```
#> hesc_320 0.8124780 0.5709904 0.5603829 0.207666387 0.7122243 0.4420199 1.383468
#> hesc_121 0.7724535 0.5400118 0.6161642 0.258183420 0.7801759 0.5120667 1.312465
#> hesc_221 0.8014791 0.5614000 0.5773216 0.221296386 0.7549133 0.4598054 1.362879
#> hesc_321 0.7959800 0.5525867 0.5931449 0.228181234 0.7886040 0.4774607 1.348567
#> hesc_122 0.9392394 0.6410682 0.4446195 0.062684877 0.5077998 0.3270849 1.580308
#> hesc_222 0.9942471 0.6610424 0.4139373 0.005769525 0.5110981 0.2934973 1.655289
#> hesc_322 0.9745975 0.6524122 0.4270787 0.025730695 0.5167165 0.3046494 1.627010
#>          cancer     abs_ent chr sample
#> hesc_1        0 0.239198693   1 hesc_1
#> hesc_2        0 0.199315150   1 hesc_2
#> hesc_3        0 0.208455338   1 hesc_3
#> hesc_11       0 0.235945962   2 hesc_1
#> hesc_21       0 0.195168024   2 hesc_2
#> hesc_31       0 0.204952619   2 hesc_3
#> hesc_12       0 0.234037405   3 hesc_1
#> hesc_22       0 0.193634002   3 hesc_2
#> hesc_32       0 0.203164889   3 hesc_3
#> hesc_13       0 0.216152187   4 hesc_1
#> hesc_23       0 0.169622460   4 hesc_2
#> hesc_33       0 0.183565201   4 hesc_3
#> hesc_14       0 0.224154615   5 hesc_1
#> hesc_24       0 0.178726075   5 hesc_2
#> hesc_34       0 0.191881615   5 hesc_3
#> hesc_15       0 0.234407256   6 hesc_1
#> hesc_25       0 0.193969483   6 hesc_2
#> hesc_35       0 0.203517051   6 hesc_3
#> hesc_16       0 0.238273965   7 hesc_1
#> hesc_26       0 0.197868162   7 hesc_2
#> hesc_36       0 0.207395395   7 hesc_3
#> hesc_17       0 0.229010988   8 hesc_1
#> hesc_27       0 0.183688725   8 hesc_2
#> hesc_37       0 0.196765312   8 hesc_3
#> hesc_18       0 0.239138804   9 hesc_1
#> hesc_28       0 0.197310074   9 hesc_2
#> hesc_38       0 0.207846577   9 hesc_3
#> hesc_19       0 0.235530741  10 hesc_1
#> hesc_29       0 0.192423306  10 hesc_2
#> hesc_39       0 0.203881732  10 hesc_3
#> hesc_110      0 0.230265034  11 hesc_1
#> hesc_210      0 0.184664946  11 hesc_2
#> hesc_310      0 0.197933281  11 hesc_3
#> hesc_111      0 0.237352561  12 hesc_1
#> hesc_211      0 0.197257308  12 hesc_2
#> hesc_311      0 0.206570649  12 hesc_3
#> hesc_112      0 0.225776715  13 hesc_1
#> hesc_212      0 0.181623191  13 hesc_2
#> hesc_312      0 0.193859792  13 hesc_3
#> hesc_113      0 0.235063615  14 hesc_1
#> hesc_213      0 0.193215293  14 hesc_2
#> hesc_313      0 0.203790025  14 hesc_3
#> hesc_114      0 0.238181037  15 hesc_1
#> hesc_214      0 0.198240793  15 hesc_2
#> hesc_314      0 0.207431131  15 hesc_3
#> hesc_115      0 0.252372820  16 hesc_1
#> hesc_215      0 0.212253762  16 hesc_2
#> hesc_315      0 0.221639207  16 hesc_3
#> hesc_116      0 0.249876316  17 hesc_1
#> hesc_216      0 0.214935851  17 hesc_2
#> hesc_316      0 0.220481138  17 hesc_3
#> hesc_117      0 0.228592076  18 hesc_1
#> hesc_217      0 0.183660769  18 hesc_2
```

```
#> hesc_317      0 0.196455708  18 hesc_3
#> hesc_118      0 0.251776558  19 hesc_1
#> hesc_218      0 0.215934487  19 hesc_2
#> hesc_318      0 0.222172975  19 hesc_3
#> hesc_119      0 0.237200390  20 hesc_1
#> hesc_219      0 0.191699149  20 hesc_2
#> hesc_319      0 0.204891433  20 hesc_3
#> hesc_120      0 0.239691773  21 hesc_1
#> hesc_220      0 0.195233975  21 hesc_2
#> hesc_320      0 0.207666387  21 hesc_3
#> hesc_121      0 0.258183420  22 hesc_1
#> hesc_221      0 0.221296386  22 hesc_2
#> hesc_321      0 0.228181234  22 hesc_3
#> hesc_122      0 0.062684877   X hesc_1
#> hesc_222      0 0.005769525   X hesc_2
#> hesc_322      0 0.025730695   X hesc_3
```

F.4 Plotting linear regression for Cancer dataset

K-Means clustering is applyed to grouping the cancer sample into two subgroups

```
km <- kmeans(bf3[, c("ent", "nu")], 2, iter.max = 100, nstart = 100)
cutpoint <- min(max( bf3$nu[ km$cluster == 1] ), max( bf3$nu[ km$cluster == 2] ))

bf3. <- bf3[ bf3$nu <= cutpoint, ]
bf3.0 <- bf3[ bf3$nu > cutpoint, ]

cutpoint
#> [1] 0.6981619
```

F.4.1 The graphic $\frac{|S|}{k_B}$ vs ν

```
ggplot(bf1, aes(x = nu, y = abs_ent)) +
    theme_light(base_family = "serif") +
    theme(text = element_text(size = 10, family = "serif")) +
    geom_point(color="red") +
    geom_smooth(data = bf3., method=lm , color = "coral", se = TRUE) +
   geom_text(x = 0.4, y = 1.35, label = lm_eqn2(x=bf3.$nu, y=bf3.$abs_ent),
            parse = TRUE, family = "serif", size = 4, color = "coral") +

    geom_smooth(data = bf3.0, method=lm , color = "red", se = TRUE) +

    geom_text(x = 1.1, y = 1.1, label = lm_eqn2(x=bf3.0$nu, y=bf3.0$abs_ent),
            parse = TRUE, family = "serif", size = 4, color = "red") +
     geom_vline(xintercept=cutpoint, linetype="dashed", color = "red") +

    geom_text(x = 0.4, y = 1.9, label = lm_eqn2(x=bf2$nu, y=bf2$abs_ent),
            parse = TRUE, family = "serif",
            color = "blue", size = 4) +
    geom_smooth(data = bf4, method=lm , se = TRUE) +
    geom_point(data = bf4, color = "blue") +

    geom_text(x = 0.3, y = -0.07, label = lm_eqn2(x=bf5$nu, y=bf5$abs_ent),
            parse = TRUE, family = "serif",
```

```
           color = "magenta",  size = 4) +
    geom_point(data = bf5, color = "magenta") +
    geom_smooth(data = bf5, method=lm , se = TRUE, color = "magenta")
```

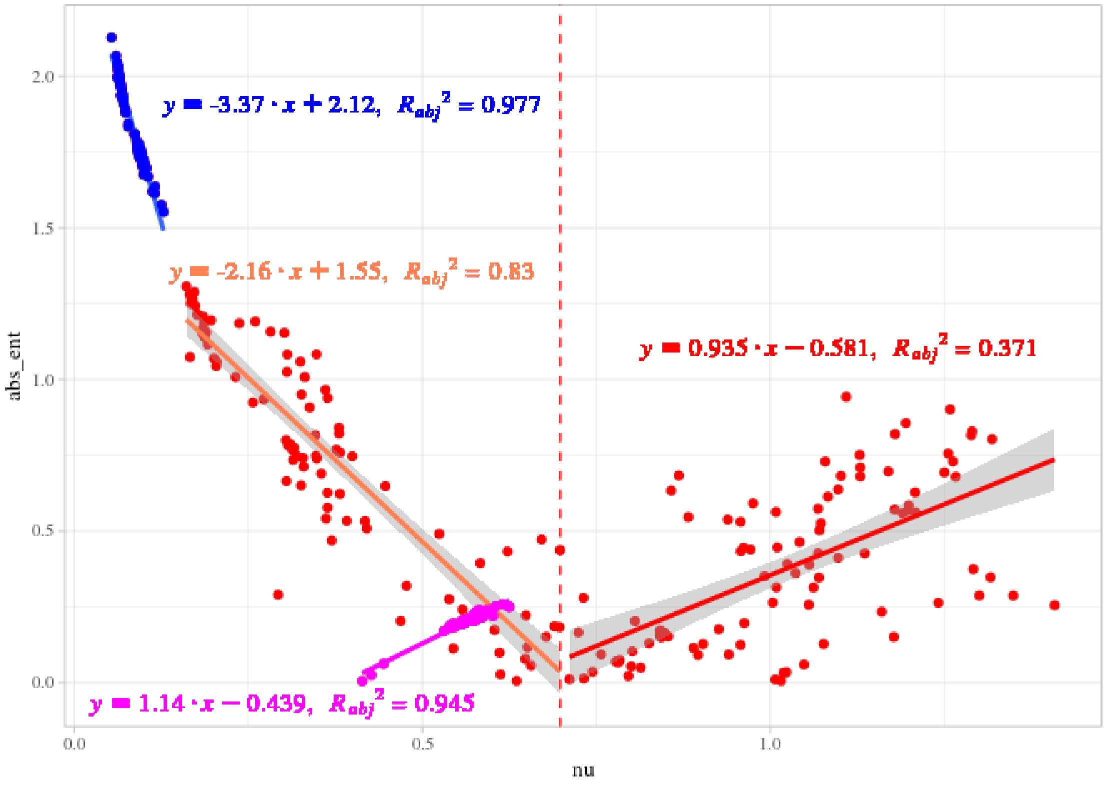

F4.2 The graphic $e^{-\frac{|S|}{k_B}}$ $vs\ \nu$

```
ggplot(bf1, aes(x = nu, y = exp_ent)) +
    theme_light(base_family = "serif") +
    theme(text = element_text(size = 10, family = "serif")) +
    geom_point(color="red") +

    geom_text(x = 0.4, y = 0.24, label = lm_eqn2(x=bf3.$nu, y=bf3.$exp_ent),
            parse = TRUE, family = "serif", size = 4, color = "coral") +
    geom_smooth(data = bf3., method=lm , color = "coral", se = TRUE) +

    geom_smooth(data = bf3.0, method=lm , color = "red", se = TRUE) +
    geom_vline(xintercept = cutpoint, linetype="dashed", color = "red") +
    geom_text(x = 1.1, y = 0.33, label = lm_eqn2(x=bf3.0$nu, y=bf3.0$exp_ent),
            parse = TRUE, family = "serif",
            color = "red",  size = 4) +

    geom_smooth(data = bf4, method=lm , se = TRUE) +
    geom_point(data = bf4, color = "blue") +
    geom_text(x = 0.4, y = 0.15, label = lm_eqn2(x=bf4$nu, y=bf4$exp_ent),
            parse = TRUE, family = "serif",
            color = "blue",  size = 4) +

    geom_point(data = bf5, color = "magenta") +
    geom_smooth(data = bf5, method=lm , se = TRUE, color = "magenta") +
    geom_text(x = 0.3, y = 1., label = lm_eqn2(x=bf5$nu, y=bf5$exp_ent),
```

```
            parse = TRUE, family = "serif",
            color = "magenta",  size = 4)
```

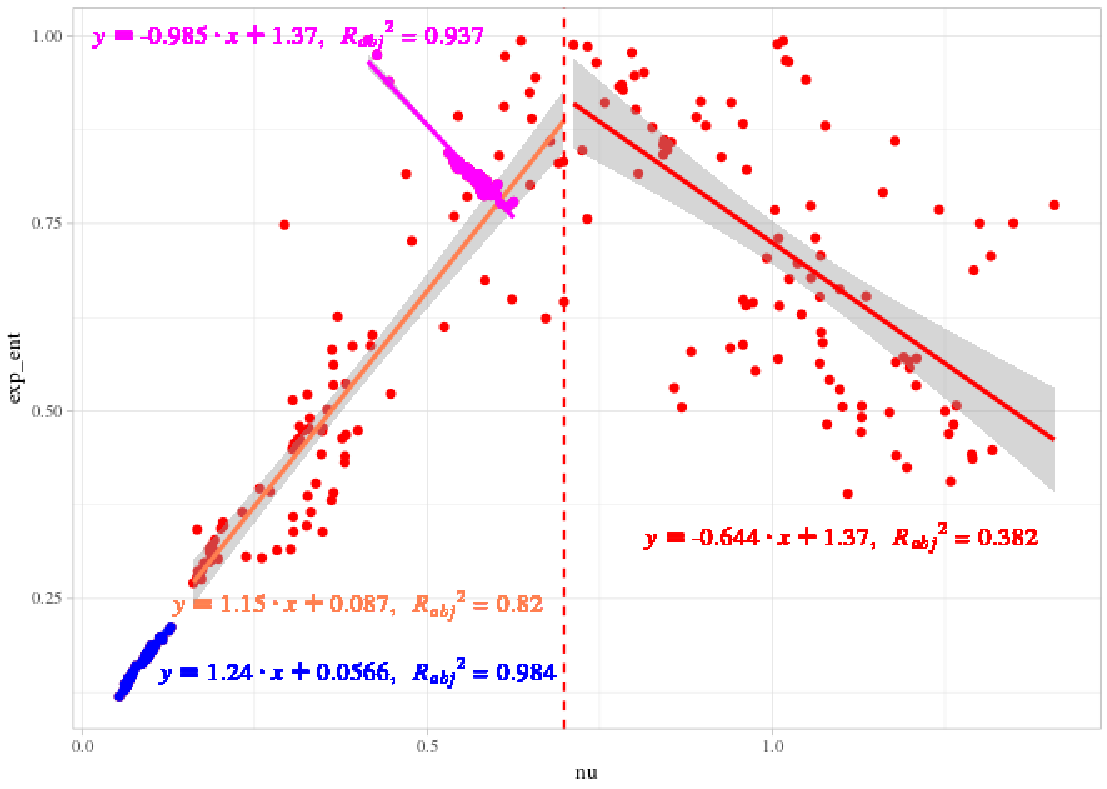

Regression analysis for healthy tissue without embryonic stem cells

```
bf4 <- bf2[ !grepl("hesc", bf2$sample), ] ## Without stem cells

lm_bf4 <- lm(exp_ent ~ nu, data = bf4)
summary(lm_bf4)
#>
#> Call:
#> lm(formula = exp_ent ~ nu, data = bf4)
#>
#> Residuals:
#>        Min         1Q     Median         3Q        Max
#> -0.0063548 -0.0020033 -0.0003749  0.0019191  0.0070402
#>
#> Coefficients:
#>             Estimate Std. Error t value Pr(>|t|)
#> (Intercept) 0.056551   0.001388   40.75   <2e-16 ***
#> nu          1.241676   0.016585   74.87   <2e-16 ***
#> ---
#> Signif. codes:  0 '***' 0.001 '**' 0.01 '*' 0.05 '.' 0.1 ' ' 1
#>
#> Residual standard error: 0.002767 on 90 degrees of freedom
#> Multiple R-squared:  0.9842, Adjusted R-squared:  0.984
#> F-statistic:  5605 on 1 and 90 DF,  p-value: < 2.2e-16
```

F4.3 The graphic $e^{-\frac{|S|}{k_B}}$ *vs* $e^{-\nu}$

```
ggplot(bf1, aes(x = exp_nu, y = exp_ent)) +
    theme_light(base_family = "serif") +
    theme(text = element_text(size = 10, family = "serif")) +
    geom_point(color="red") +

    geom_text(x = 0.8, y = 0.65, label = lm_eqn2(x=bf3.$exp_nu, y=bf3.$exp_ent),
            parse = TRUE, family = "serif", size = 4, color = "coral") +
    geom_smooth(data = bf3., method=lm , color = "coral", se = TRUE) +

    geom_smooth(data = bf3.0, method=lm , color = "red", se = TRUE) +
    geom_vline(xintercept = exp(-cutpoint), linetype="dashed", color = "red") +
        geom_text(x = 0.37, y = 0.33, label = lm_eqn2(x=bf3.0$exp_nu, y=bf3.0$exp_ent),
            parse = TRUE, family = "serif",
            color = "red",  size = 4) +

    geom_smooth(data = bf4, method=lm , se = TRUE) +
    geom_point(data = bf4, color = "blue") +
    geom_text(x = 0.74, y = 0.15, label = lm_eqn2(x=bf4$exp_nu, y=bf4$exp_ent),
            parse = TRUE, family = "serif",
            color = "blue",  size = 4) +

    geom_point(data = bf5, color = "magenta") +
    geom_smooth(data = bf5, method=lm , se = TRUE, color = "magenta") +
    geom_text(x = 0.8, y = 0.8, label = lm_eqn2(x=bf5$exp_nu, y=bf5$exp_ent),
            parse = TRUE, family = "serif",
            color = "magenta",  size = 4)
```

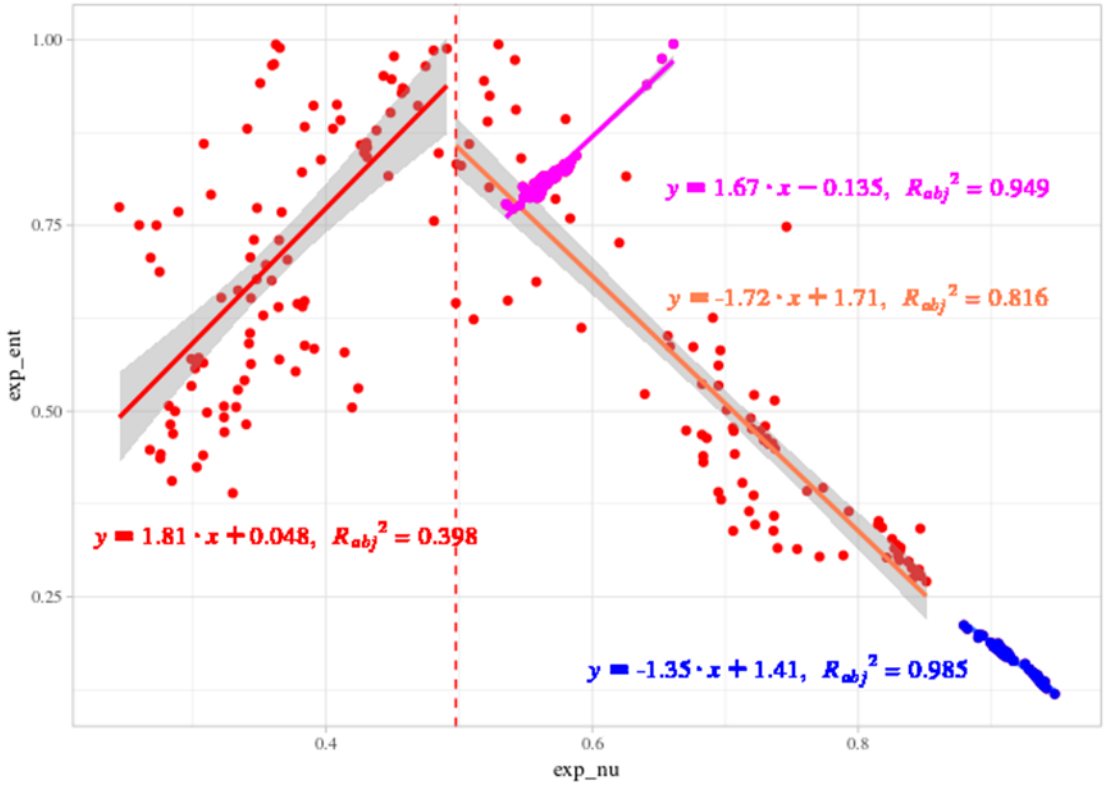

F.5 Bar and box plots

```
library(ggplot2)
library(ggpmisc)
```

F5.1 Summarize by species

```
dt$species <- "human"

dat <- rbind(df, dt)
st <- dat %>% group_by(species) %>%
    summarize(mean = round(mean(means), 3),
              n = n(), sd = round(sd(means), 3),
              se = round(sd / sqrt(n), 3))
colnames(st) <- c("species", " mean ", " sample.size ",
                  " standard.dev ", " standard.Err " )

chr <- c("chr1", "chr2", "chr3", "chr4", "chr5",
        paste(1:22), "X")

dat
```

F5.2 The barplot

```
p <- ggplot(dat, aes(x = chr, y = means, fill = species)) +
    scale_x_discrete(limits = chr) +
    scale_fill_manual("Species",
        values = c("Arabidopsis" = "deepskyblue", "human" = "dodgerblue3")) +

    geom_bar(stat="identity", position=position_dodge()) +
    geom_errorbar(aes(ymin = means - sd, ymax = means + sd), width =.2,
                  position=position_dodge(.9)) +

    geom_hline(yintercept = 1., linetype="dashed", color = "red") +
    xlab("Chromosome") + ylab("") +
    geom_text(aes(label = n), colour = "white", fontface = "bold",
              nudge_y = -0.5, family = "serif") +

    theme_light(base_family = "serif", base_size = 14) +
    theme(text = element_text(size = 16, family = "serif"),
          legend.margin=margin(4,4,4,4),
          legend.box.spacing = margin(0.5),
          legend.position="bottom") +
    annotate(geom = "table",
             x = 25,
             y = 1.8,
             label = list(st),
             size = 5,
             family = "serif")

p + scale_fill_brewer(palette="Paired")
```

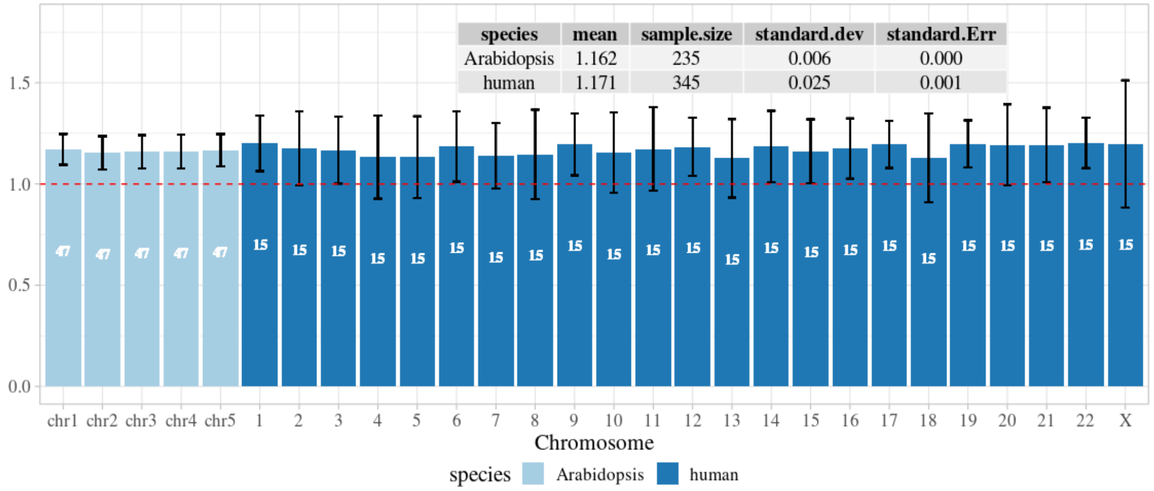

F5.3 The boxplot

Disruption of breast cancer and stem cells

```
st1. <- data.frame(dt.)[ is.element(dt.$sample, c("Brain", "Breast", "Colon", "Lung")), ]
st1. <- st1. %>% group_by(sample) %>%
    summarize(mean = round(mean(means),3),
              n = n(), sd = round(sd(means),3),
              se = round(sd / sqrt(n), 3))

ggplot(dt., aes(x = sample, y = means, fill = sample, color = sample, )) +

        geom_rect(xmin = 0.5, xmax = 2.5, ymin = -Inf, ymax = Inf,
                fill = "gray92", colour = "gray98", alpha = 0.05) +

        geom_rect(xmin = 5.5, xmax = 8.5, ymin = -Inf, ymax = Inf,
                fill = "gray92", colour = "gray98", alpha = 0.05) +

        geom_rect(xmin = 12.5, xmax = 15.5, ymin = -Inf, ymax = Inf,
                fill = "gray92", colour = "gray98", alpha = 0.05) +

        geom_rect(ymin = 1.06, ymax = 1.1,
                  xmin = -Inf, xmax = Inf, fill = 'lightskyblue',
                  color = "lightskyblue", alpha = 0.5) +
        geom_rect(ymin = 1.31, ymax = 1.43,
                  xmin = -Inf, xmax = Inf, fill = 'lightgreen', color = "lightgreen", alph
a = 0.06) +

        geom_boxplot(shape=21, alpha = 0.9, color = "red") +
        xlab("") + ylab("") +
        theme_light(base_family = "serif", base_size = 14) +
        theme(
            panel.background = element_rect(colour = "gray10"),
            text = element_text(size = 16, family = "serif"),
            axis.text.x = element_text(angle = 45, vjust = 1, hjust=1),
            legend.title=element_blank(),
            legend.margin=margin(4,4,4,4),
            legend.box.spacing = margin(0.5)) +
```

```
        annotate(geom = "table",
                 x = 2.5,
                 y = 1.7,
                 label = list(st1.),
                 size = 5,
                 family = "serif")
```

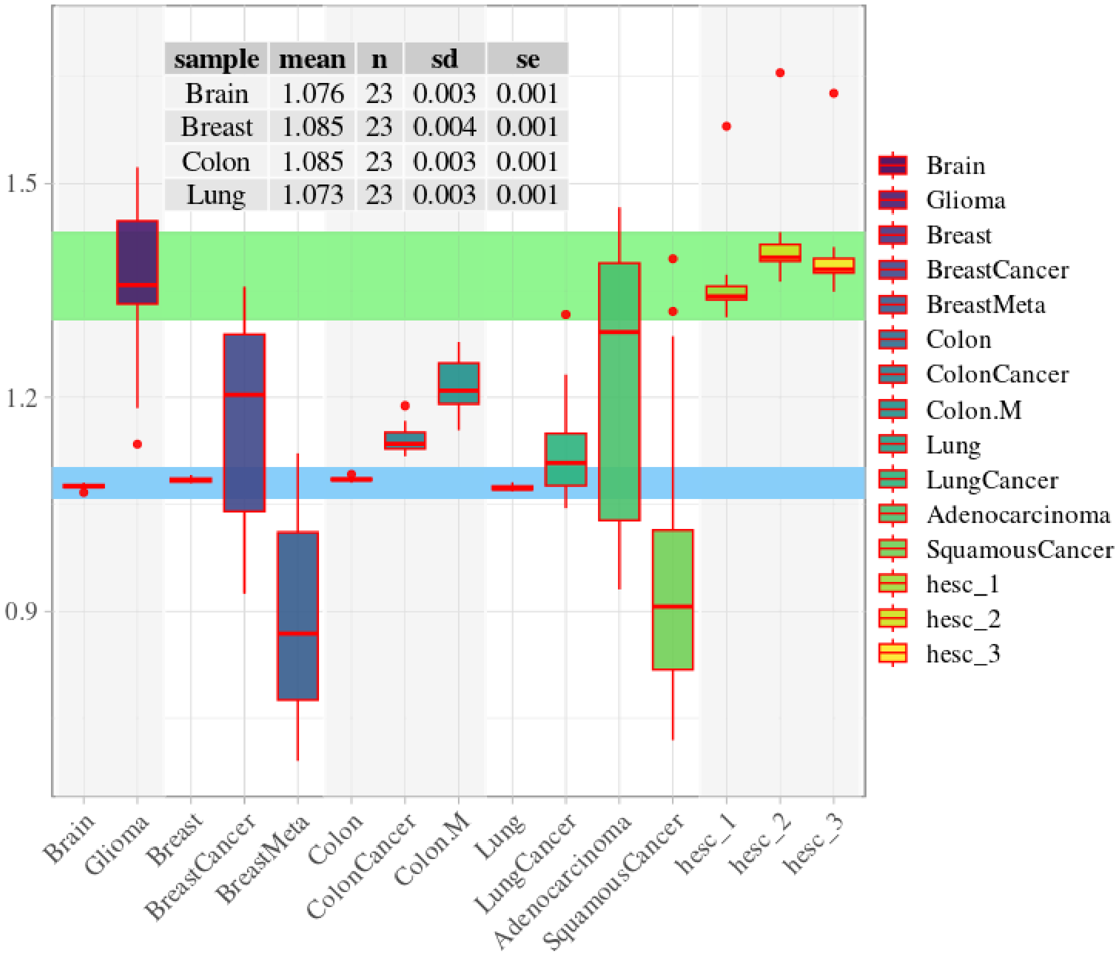

G Group differences in Arabidopsis memory line-based entropy

The group comparisons ‘control’ versus *msh1* memory lines are presented here. We are interested to learn whether the Gibb entropy (gent) of methylation variation, measured with respect to some reference state, coincides with observable phenotypic change. gent was estimated in *Arabidopsis thaliana* Col-0 (wildtype control, WT), the methyltransferase mutant *met1* (1), and first- and third-generation heritable epigenetic memory states (*nm1*, *mm1*, and *mm3*), which derive as epigenetically modified progeny from a parental line following suppression of *MSH1* expression.

```r
library(MethylIT)
#> Warning: replacing previous import 'lifecycle::last_warnings' by
#> 'rlang::last_warnings' when loading 'tibble'
#> Warning: replacing previous import 'lifecycle::last_warnings' by
#> 'rlang::last_warnings' when loading 'pillar'
library(lmerTest)
library(lme4)
library(reshape2)
```

Next, an auxiliary function to format the datasets into 'data.frames'

```r
gent <- function(x) {
    x <- t(sapply(x, gibb_entropy))
    colnames(x) <- paste0("chr", colnames(x))
    x = melt(x)
    colnames(x) <- c("sample", "chr", "entropy")
    x$chr <- factor(x$chr)
    return(x)
}
```

The RData files with nonlinear fit models can be downloaded from GitLab using the following script:

```r
## ===== Download methylation data from GitLab ========
samples <- c("memory-col0-control-gen3",
             "non-memory-gen1",
             "memory-gen1",
             "memory-gen3",
             "col0-control-met1",
             "met1")

url <- paste0("https://git.psu.edu/genomath/datasets/-/raw/main/at_mutants/",
           "gofs/gof-jd-by-chr_", samples,"_all-contexts.RData")

nms <- c("ctrl", "nm", "mm1", "mm3", "ctrl_met1", "met1")
gofs <- vector(mode = "list", length = 6)
names(gofs) <- nms
for (k in seq(samples)) {
    temp <- tempfile(fileext = ".RData")
    download.file(url = url[k], destfile = temp)
    load(temp)
    file.remove(temp)

    gent_wt <- gent(gof_jd)
    gent_wt$group <- nms[ k ]
    gent_wt$group <- factor(gent_wt$group)

    gofs[[k]] <- gent_wt
}
rm(temp, url)
gofs
#> $ctrl
#>    sample  chr   entropy group
#> 1   wt3_1 chr1 -12.09485  ctrl
#> 2   wt3_2 chr1 -12.23864  ctrl
#> 3   wt3_3 chr1 -12.58199  ctrl
#> 4   wt3_4 chr1 -12.19005  ctrl
#> 5   wt3_5 chr1 -13.00950  ctrl
#> 6   wt3_1 chr2 -13.09161  ctrl
#> 7   wt3_2 chr2 -13.20160  ctrl
```

```
#> 8   wt3_3 chr2 -13.61125  ctrl
#> 9   wt3_4 chr2 -13.28911  ctrl
#> 10  wt3_5 chr2 -14.07444  ctrl
#> 11  wt3_1 chr3 -12.85370  ctrl
#> 12  wt3_2 chr3 -12.82698  ctrl
#> 13  wt3_3 chr3 -13.31159  ctrl
#> 14  wt3_4 chr3 -12.88366  ctrl
#> 15  wt3_5 chr3 -13.80581  ctrl
#> 16  wt3_1 chr4 -12.87477  ctrl
#> 17  wt3_2 chr4 -12.95472  ctrl
#> 18  wt3_3 chr4 -13.40339  ctrl
#> 19  wt3_4 chr4 -13.00763  ctrl
#> 20  wt3_5 chr4 -13.83110  ctrl
#> 21  wt3_1 chr5 -12.39782  ctrl
#> 22  wt3_2 chr5 -12.44664  ctrl
#> 23  wt3_3 chr5 -12.87161  ctrl
#> 24  wt3_4 chr5 -12.53417  ctrl
#> 25  wt3_5 chr5 -13.33332  ctrl
#> 
#> $nm
#>    sample  chr   entropy group
#> 1   nm1_1 chr1 -10.51669    nm
#> 2   nm1_2 chr1 -10.34410    nm
#> 3   nm1_3 chr1 -13.42377    nm
#> 4   nm1_4 chr1 -10.33168    nm
#> 5   nm1_5 chr1 -14.45793    nm
#> 6   nm1_1 chr2 -11.67142    nm
#> 7   nm1_2 chr2 -11.46055    nm
#> 8   nm1_3 chr2 -14.23364    nm
#> 9   nm1_4 chr2 -11.42840    nm
#> 10  nm1_5 chr2 -14.97243    nm
#> 11  nm1_1 chr3 -11.42988    nm
#> 12  nm1_2 chr3 -11.19313    nm
#> 13  nm1_3 chr3 -14.12629    nm
#> 14  nm1_4 chr3 -11.16044    nm
#> 15  nm1_5 chr3 -15.00178    nm
#> 16  nm1_1 chr4 -11.44687    nm
#> 17  nm1_2 chr4 -11.20523    nm
#> 18  nm1_3 chr4 -14.17518    nm
#> 19  nm1_4 chr4 -11.19233    nm
#> 20  nm1_5 chr4 -14.80361    nm
#> 21  nm1_1 chr5 -10.97023    nm
#> 22  nm1_2 chr5 -10.75758    nm
#> 23  nm1_3 chr5 -13.76125    nm
#> 24  nm1_4 chr5 -10.73998    nm
#> 25  nm1_5 chr5 -14.61423    nm
#> 
#> $mm1
#>    sample  chr    entropy group
#> 1    m1_1 chr1 -12.452386   mm1
#> 2    m1_2 chr1 -13.169982   mm1
#> 3    m1_3 chr1 -10.484701   mm1
#> 4    m1_4 chr1 -10.087015   mm1
#> 5    m1_5 chr1  -9.969216   mm1
#> 6    m1_1 chr2 -13.384541   mm1
#> 7    m1_2 chr2 -14.111095   mm1
#> 8    m1_3 chr2 -11.578029   mm1
#> 9    m1_4 chr2 -11.177138   mm1
#> 10   m1_5 chr2 -11.103592   mm1
#> 11   m1_1 chr3 -13.152839   mm1
#> 12   m1_2 chr3 -13.933727   mm1
```

```
#> 13   m1_3 chr3 -11.390940   mm1
#> 14   m1_4 chr3 -10.971958   mm1
#> 15   m1_5 chr3 -10.818339   mm1
#> 16   m1_1 chr4 -13.134270   mm1
#> 17   m1_2 chr4 -13.978335   mm1
#> 18   m1_3 chr4 -11.368858   mm1
#> 19   m1_4 chr4 -10.981584   mm1
#> 20   m1_5 chr4 -10.851584   mm1
#> 21   m1_1 chr5 -12.807018   mm1
#> 22   m1_2 chr5 -13.578820   mm1
#> 23   m1_3 chr5 -10.946872   mm1
#> 24   m1_4 chr5 -10.484795   mm1
#> 25   m1_5 chr5 -10.297509   mm1
#>
#> $mm3
#>    sample  chr    entropy group
#> 1    m3_1 chr1  -9.504246   mm3
#> 2    m3_2 chr1  -9.617158   mm3
#> 3    m3_3 chr1  -9.391835   mm3
#> 4    m3_4 chr1 -10.335803   mm3
#> 5    m3_5 chr1  -9.687667   mm3
#> 6    m3_1 chr2 -10.593100   mm3
#> 7    m3_2 chr2 -10.690615   mm3
#> 8    m3_3 chr2 -10.474706   mm3
#> 9    m3_4 chr2 -11.406912   mm3
#> 10   m3_5 chr2 -10.736263   mm3
#> 11   m3_1 chr3 -10.365805   mm3
#> 12   m3_2 chr3 -10.536732   mm3
#> 13   m3_3 chr3 -10.269382   mm3
#> 14   m3_4 chr3 -11.292288   mm3
#> 15   m3_5 chr3 -10.530887   mm3
#> 16   m3_1 chr4 -10.370156   mm3
#> 17   m3_2 chr4 -10.527998   mm3
#> 18   m3_3 chr4 -10.263898   mm3
#> 19   m3_4 chr4 -11.310295   mm3
#> 20   m3_5 chr4 -10.526185   mm3
#> 21   m3_1 chr5  -9.850248   mm3
#> 22   m3_2 chr5 -10.013634   mm3
#> 23   m3_3 chr5  -9.839075   mm3
#> 24   m3_4 chr5 -10.824549   mm3
#> 25   m3_5 chr5 -10.083295   mm3
#>
#> $ctrl_met1
#>    sample  chr   entropy     group
#> 1    wt_1 chr1 -3.751485 ctrl_met1
#> 2    wt_2 chr1 -5.876468 ctrl_met1
#> 3    wt_3 chr1 -5.869031 ctrl_met1
#> 4    wt_4 chr1 -5.993948 ctrl_met1
#> 5    wt_1 chr2 -4.060561 ctrl_met1
#> 6    wt_2 chr2 -6.242450 ctrl_met1
#> 7    wt_3 chr2 -6.215879 ctrl_met1
#> 8    wt_4 chr2 -6.346747 ctrl_met1
#> 9    wt_1 chr3 -3.957873 ctrl_met1
#> 10   wt_2 chr3 -6.163961 ctrl_met1
#> 11   wt_3 chr3 -6.070257 ctrl_met1
#> 12   wt_4 chr3 -6.177509 ctrl_met1
#> 13   wt_1 chr4 -3.738039 ctrl_met1
#> 14   wt_2 chr4 -5.959253 ctrl_met1
#> 15   wt_3 chr4 -5.895751 ctrl_met1
#> 16   wt_4 chr4 -5.994613 ctrl_met1
#> 17   wt_1 chr5 -3.700360 ctrl_met1
```

```
#> 18   wt_2 chr5 -5.810828 ctrl_met1
#> 19   wt_3 chr5 -5.727038 ctrl_met1
#> 20   wt_4 chr5 -5.889276 ctrl_met1
#>
#> $met1
#>    sample  chr  entropy group
#> 1  met1_1 chr1 2.183079  met1
#> 2  met1_2 chr1 1.199392  met1
#> 3  met1_3 chr1 2.032322  met1
#> 4  met1_1 chr2 2.128942  met1
#> 5  met1_2 chr2 1.126149  met1
#> 6  met1_3 chr2 1.993428  met1
#> 7  met1_1 chr3 2.065479  met1
#> 8  met1_2 chr3 1.072355  met1
#> 9  met1_3 chr3 1.923155  met1
#> 10 met1_1 chr4 1.980010  met1
#> 11 met1_2 chr4 1.004305  met1
#> 12 met1_3 chr4 1.847710  met1
#> 13 met1_1 chr5 2.085467  met1
#> 14 met1_2 chr5 1.107616  met1
#> 15 met1_3 chr5 1.945829  met1
```

G.1 Linear and generalized linear models for memory line 1rt generation

```
    gent_wt_mm1 <- rbind(gofs$ctrl, gofs$mm1)
lm_wt_mm1 <- lmer(formula = entropy ~ group + (1|chr),
                  data = gent_wt_mm1)
summary(lm_wt_mm1)
#> Linear mixed model fit by REML. t-tests use Satterthwaite's method [
#> lmerModLmerTest]
#> Formula: entropy ~ group + (1 | chr)
#>    Data: gent_wt_mm1
#>
#> REML criterion at convergence: 144.9
#>
#> Scaled residuals:
#>     Min      1Q  Median      3Q     Max
#> -2.0750 -0.6688  0.1694  0.6279  1.6312
#>
#> Random effects:
#>  Groups   Name        Variance Std.Dev.
#>  chr      (Intercept) 0.07153  0.2675
#>  Residual             1.00269  1.0013
#> Number of obs: 50, groups:  chr, 5
#>
#> Fixed effects:
#>             Estimate Std. Error       df t value Pr(>|t|)
#> (Intercept) -12.9888     0.2333   9.7303 -55.682 1.63e-13 ***
#> groupmm1      1.1402     0.2832  44.0000   4.026 0.000221 ***
#> ---
#> Signif. codes:  0 '***' 0.001 '**' 0.01 '*' 0.05 '.' 0.1 ' ' 1
#>
#> Correlation of Fixed Effects:
#>          (Intr)
#> groupmm1 -0.607
anova(lm_wt_mm1)
#> Type III Analysis of Variance Table with Satterthwaite's method
#>       Sum Sq Mean Sq NumDF DenDF F value    Pr(>F)
#> group  16.25   16.25     1    44  16.207 0.0002207 ***
```

```
#> ---
#> Signif. codes:  0 '***' 0.001 '**' 0.01 '*' 0.05 '.' 0.1 ' ' 1
```

G.2 Linear and generalized linear models for non-memory line

```
gent_wt_nm <- rbind(gofs$ctrl, gofs$nm)
lm_wt_nm <- lmer(formula = entropy ~ group + (1|chr),
                 data = gent_wt_nm)
#> boundary (singular) fit: see ?isSingular
summary(lm_wt_nm)
#> Linear mixed model fit by REML. t-tests use Satterthwaite's method [
#> lmerModLmerTest]
#> Formula: entropy ~ group + (1 | chr)
#>    Data: gent_wt_nm
#>
#> REML criterion at convergence: 165.3
#>
#> Scaled residuals:
#>      Min       1Q   Median       3Q      Max
#> -2.07391 -0.60705  0.09134  0.73194  1.61570
#>
#> Random effects:
#>  Groups   Name        Variance  Std.Dev.
#>  chr      (Intercept) 5.322e-15 7.295e-08
#>  Residual             1.602e+00 1.266e+00
#> Number of obs: 50, groups:  chr, 5
#>
#> Fixed effects:
#>             Estimate Std. Error       df t value Pr(>|t|)
#> (Intercept) -12.9888     0.2531  48.0000  -51.31   <2e-16 ***
#> groupnm       0.6121     0.3580  48.0000    1.71   0.0938 .
#> ---
#> Signif. codes:  0 '***' 0.001 '**' 0.01 '*' 0.05 '.' 0.1 ' ' 1
#>
#> Correlation of Fixed Effects:
#>         (Intr)
#> groupnm -0.707
#> optimizer (nloptwrap) convergence code: 0 (OK)
#> boundary (singular) fit: see ?isSingular
anova(lm_wt_nm)
#> Type III Analysis of Variance Table with Satterthwaite's method
#>       Sum Sq Mean Sq NumDF DenDF F value Pr(>F)
#> group 4.6826  4.6826     1    48  2.9228 0.0938 .
#> ---
#> Signif. codes:  0 '***' 0.001 '**' 0.01 '*' 0.05 '.' 0.1 ' ' 1
```

The linear mixed model cannot be fitted. Alternatively, if the linear mixed model fails, then t-test for paired samples can be applied if the difference x - y follow normal distribution. Otherwise, Wilcoxon signed rank test can be applied.

```
t.test(gent_wt_nm$entropy[ gent_wt_nm$group == "ctrl"],
       gent_wt_nm$entropy[ gent_wt_nm$group == "nm"],
       paired = TRUE,
       alternative = "greater")
#>
#>  Paired t-test
#>
#> data:  gent_wt_nm$entropy[gent_wt_nm$group == "ctrl"] and gent_wt_nm$entropy[gent_wt_nm
$group == "nm"]
```

```
#> t = -2.2885, df = 24, p-value = 0.9844
#> alternative hypothesis: true difference in means is greater than 0
#> 95 percent confidence interval:
#>  -1.069629       Inf
#> sample estimates:
#> mean of the differences
#>              -0.6120537
```

Shapiro-Wilk normality test indicates that the Paired t-test result is not valid since the differences ‘d’ does not follow normal distribution.

```
d = gent_wt_nm$entropy[ gent_wt_nm$group == "ctrl" ] -
    gent_wt_nm$entropy[ gent_wt_nm$group == "nm" ]
shapiro.test(d)
#>
#>  Shapiro-Wilk normality test
#>
#> data:  d
#> W = 0.75012, p-value = 3.727e-05
```

Alternatively, the Wilcoxon signed rank test, which does not depend on the normality hypothesis, can be applied.

```
wilcox.test(gent_wt_nm$entropy[ gent_wt_nm$group == "nm" ],
       gent_wt_nm$entropy[ gent_wt_nm$group == "ctrl" ],
       paired = TRUE,
       alternative = "greater")
#>
#>  Wilcoxon signed rank exact test
#>
#> data:  gent_wt_nm$entropy[gent_wt_nm$group == "nm"] and gent_wt_nm$entropy[gent_wt_nm$g
roup == "ctrl"]
#> V = 266, p-value = 0.002088
#> alternative hypothesis: true location shift is greater than 0
```

G.3 Linear and generalized linear models for memory line 3rd generation

```
gent_wt_mm3 <- rbind(gofs$ctrl, gofs$mm3)
lm_wt_mm3 <- lmer(formula = entropy ~ group + (1|chr),
                  data = gent_wt_mm3)
summary(lm_wt_mm3)
#> Linear mixed model fit by REML. t-tests use Satterthwaite's method [
#> lmerModLmerTest]
#> Formula: entropy ~ group + (1 | chr)
#>    Data: gent_wt_mm3
#>
#> REML criterion at convergence: 59.4
#>
#> Scaled residuals:
#>     Min      1Q  Median      3Q     Max
#> -1.9931 -0.4040  0.3778  0.7427  1.0794
#>
#> Random effects:
#>  Groups   Name        Variance Std.Dev.
#>  chr      (Intercept) 0.1666   0.4081
#>  Residual             0.1429   0.3780
#> Number of obs: 50, groups:  chr, 5
#>
#> Fixed effects:
```

```
#>             Estimate Std. Error       df t value Pr(>|t|)
#> (Intercept) -12.9888     0.1976   4.6543  -65.74 4.34e-08 ***
#> groupmm3      2.6271     0.1069  44.0000   24.57  < 2e-16 ***
#> ---
#> Signif. codes:  0 '***' 0.001 '**' 0.01 '*' 0.05 '.' 0.1 ' ' 1
#>
#> Correlation of Fixed Effects:
#>          (Intr)
#> groupmm3 -0.271
anova(lm_wt_mm3)
#> Type III Analysis of Variance Table with Satterthwaite's method
#>       Sum Sq Mean Sq NumDF DenDF F value    Pr(>F)
#> group  86.27   86.27     1    44  603.82 < 2.2e-16 ***
#> ---
#> Signif. codes:  0 '***' 0.001 '**' 0.01 '*' 0.05 '.' 0.1 ' ' 1
```

G.4 Linear and nonlinear models for met1

```
gent_wt_met1 <- rbind(gofs$ctrl_met1, gofs$met1)
glm_wt_met1 <- lmer(formula = entropy ~ group + (1|chr),
                     data = gent_wt_met1)
#> boundary (singular) fit: see ?isSingular
summary(glm_wt_met1)
#> Linear mixed model fit by REML. t-tests use Satterthwaite's method [
#> lmerModLmerTest]
#> Formula: entropy ~ group + (1 | chr)
#>    Data: gent_wt_met1
#>
#> REML criterion at convergence: 84.7
#>
#> Scaled residuals:
#>     Min      1Q  Median      3Q     Max
#> -1.0916 -0.7395 -0.4954  0.4192  2.2111
#>
#> Random effects:
#>  Groups   Name        Variance Std.Dev.
#>  chr      (Intercept) 0.000    0.0000
#>  Residual             0.642    0.8013
#> Number of obs: 35, groups:  chr, 5
#>
#> Fixed effects:
#>             Estimate Std. Error      df t value Pr(>|t|)
#> (Intercept)  -5.4721     0.1792 33.0000  -30.54   <2e-16 ***
#> groupmet1     7.1851     0.2737 33.0000   26.25   <2e-16 ***
#> ---
#> Signif. codes:  0 '***' 0.001 '**' 0.01 '*' 0.05 '.' 0.1 ' ' 1
#>
#> Correlation of Fixed Effects:
#>           (Intr)
#> groupmet1 -0.655
#> optimizer (nloptwrap) convergence code: 0 (OK)
#> boundary (singular) fit: see ?isSingular
anova(glm_wt_met1)
#> Type III Analysis of Variance Table with Satterthwaite's method
#>       Sum Sq Mean Sq NumDF DenDF F value    Pr(>F)
#> group  442.5   442.5     1    33  689.22 < 2.2e-16 ***
#> ---
#> Signif. codes:  0 '***' 0.001 '**' 0.01 '*' 0.05 '.' 0.1 ' ' 1
```

The linear mixed cannot be fitted. Alternatively, the t-test paired samples can be applied if the difference *x* - *y* follows normal distribution. Since there are more samples in the control than in 'met1' group, the first three samples from each chromosome from control group are taken for the paired comparison.

```
wt <- gent_wt_met1[ gent_wt_met1$group == "ctrl_met1",]
idx <- is.element(wt$sample, c("wt_1","wt_2","wt_3"))

d = gent_wt_met1$entropy[ gent_wt_met1$group == "met1"] -
    wt$entropy[ idx]
shapiro.test(d)
#>
#>  Shapiro-Wilk normality test
#>
#> data:  d
#> W = 0.9175, p-value = 0.1765

t.test(gent_wt_met1$entropy[ gent_wt_met1$group == "met1"],
      wt$entropy[ idx ],
       paired = TRUE,
       alternative = "greater")
#>
#>  Paired t-test
#>
#> data:  gent_wt_met1$entropy[gent_wt_met1$group == "met1"] and wt$entropy[idx]
#> t = 31.479, df = 14, p-value = 1.073e-14
#> alternative hypothesis: true difference in means is greater than 0
#> 95 percent confidence interval:
#>  6.591631      Inf
#> sample estimates:
#> mean of the differences
#>               6.982298
```

G.5 Linear model with fixed effects for met1

The linear model with fixed effects suggests that the failing of the linear mixed model would originate from the fact that chromosomes effects (when considered as a fixed effects) are not statistically significant.

```
gent_wt_met1 <- rbind(gofs$ctrl_met1, gofs$met1)
lm_wt_met1 <- lm(formula = entropy ~ group + chr,
                     data = gent_wt_met1)
summary(lm_wt_met1)
#>
#> Call:
#> lm(formula = entropy ~ group + chr, data = gent_wt_met1)
#>
#> Residuals:
#>     Min      1Q  Median      3Q     Max
#> -0.7507 -0.5853 -0.4474  0.3320  1.7349
#>
#> Coefficients:
#>             Estimate Std. Error t value Pr(>|t|)
#> (Intercept) -5.37591    0.34399 -15.628 1.16e-15 ***
#> groupmet1    7.18508    0.28988  24.786  < 2e-16 ***
#> chrchr2     -0.22014    0.45364  -0.485    0.631
#> chrchr3     -0.17607    0.45364  -0.388    0.701
```

```
#> chrchr4      -0.09707    0.45364  -0.214    0.832
#> chrchr5       0.01251    0.45364   0.028    0.978
#> ---
#> Signif. codes:  0 '***' 0.001 '**' 0.01 '*' 0.05 '.' 0.1 ' ' 1
#>
#> Residual standard error: 0.8487 on 29 degrees of freedom
#> Multiple R-squared:  0.955,  Adjusted R-squared:  0.9472
#> F-statistic:   123 on 5 and 29 DF,  p-value: < 2.2e-16
anova(lm_wt_met1)
#> Analysis of Variance Table
#>
#> Response: entropy
#>           Df Sum Sq Mean Sq F value Pr(>F)
#> group      1 442.50  442.50 614.364 <2e-16 ***
#> chr        4   0.30    0.07   0.104 0.9802
#> Residuals 29  20.89    0.72
#> ---
#> Signif. codes:  0 '***' 0.001 '**' 0.01 '*' 0.05 '.' 0.1 ' '
```

G.6 Result table

```
gent <- function(s) {
    t(sapply(s, gibb_entropy))
}

## ===== Download methylation data from GitLab ========
samples <- c("memory-col0-control-gen3",
            "non-memory-gen1",
            "memory-gen1",
            "memory-gen3",
            "col0-control-met1",
            "met1")

url <- paste0("https://git.psu.edu/genomath/datasets/-/raw/main/at_mutants/",
            "gofs/gof-jd-by-chr_", samples,"_all-contexts.RData")

nms <- c("ctrl", "nm", "mm1", "mm3", "msh1", "ctrl_met1", "met1")
entropy <- c()
for (k in seq(samples)) {
    temp <- tempfile(fileext = ".RData")
    download.file(url = url[k], destfile = temp)
    load(temp)
    file.remove(temp)
    entropy <- rbind(entropy, gent(gof_jd))
}
rm(temp, url)
colnames(entropy) <- paste0("Chromosome ", 1:5)
entropy
#>         Chromosome 1 Chromosome 2 Chromosome 3 Chromosome 4 Chromosome 5
#> wt3_1     -12.094846   -13.091612   -12.853697   -12.874774   -12.397823
#> wt3_2     -12.238638   -13.201601   -12.826977   -12.954719   -12.446643
#> wt3_3     -12.581987   -13.611254   -13.311590   -13.403394   -12.871608
#> wt3_4     -12.190052   -13.289105   -12.883656   -13.007626   -12.534174
#> wt3_5     -13.009502   -14.074439   -13.805811   -13.831105   -13.333324
#> nm1_1     -10.516693   -11.671416   -11.429877   -11.446873   -10.970227
#> nm1_2     -10.344103   -11.460553   -11.193130   -11.205235   -10.757582
#> nm1_3     -13.423766   -14.233640   -14.126294   -14.175181   -13.761249
#> nm1_4     -10.331681   -11.428398   -11.160442   -11.192328   -10.739979
#> nm1_5     -14.457926   -14.972428   -15.001781   -14.803605   -14.614226
#> m1_1      -12.452386   -13.384541   -13.152839   -13.134270   -12.807018
```

```
#> m1_2     -13.169982  -14.111095  -13.933727  -13.978335  -13.578820
#> m1_3     -10.484701  -11.578029  -11.390940  -11.368858  -10.946872
#> m1_4     -10.087015  -11.177138  -10.971958  -10.981584  -10.484795
#> m1_5      -9.969216  -11.103592  -10.818339  -10.851584  -10.297509
#> m3_1      -9.504246  -10.593100  -10.365805  -10.370156   -9.850248
#> m3_2      -9.617158  -10.690615  -10.536732  -10.527998  -10.013634
#> m3_3      -9.391835  -10.474706  -10.269382  -10.263898   -9.839075
#> m3_4     -10.335803  -11.406912  -11.292288  -11.310295  -10.824549
#> m3_5      -9.687667  -10.736263  -10.530887  -10.526185  -10.083295
#> wt_1      -3.751485   -4.060561   -3.957873   -3.738039   -3.700360
#> wt_2      -5.876468   -6.242450   -6.163961   -5.959253   -5.810828
#> wt_3      -5.869031   -6.215879   -6.070257   -5.895751   -5.727038
#> wt_4      -5.993948   -6.346747   -6.177509   -5.994613   -5.889276
#> met1_1     2.183079    2.128942    2.065479    1.980010    2.085467
#> met1_2     1.199392    1.126149    1.072355    1.004305    1.107616
#> met1_3     2.032322    1.993428    1.923155    1.847710    1.945829
```
